# Angiotensin II type 1 receptor signaling promotes bladder cancer progression and its inhibition by Losartan

**DOI:** 10.1038/s41440-025-02535-y

**Published:** 2026-01-19

**Authors:** Ryoken Yamanaka, Kento Miura, Norimasa Yamasaki, Sawako Ogata, Megmi Nakamura, Toshiya Inaba, Anarkhuu Bold-Erdene, Uyanga Enkhbaatar, Fatemeh Beygom Mirkatouli, Shuka Miura, Naohisa Hosomi, Kohei Kobatake, Kenshiro Takemoto, Yuki Kohada, Ryo Tasaka, Tomoya Hatayama, Kazuma Yukihiro, Hiroyuki Shikuma, Kyosuke Iwane, Nobuyuki Hinata, Osamu Kaminuma

**Affiliations:** 1 Department of Disease Models, Research Institute for Radiation Biology and Medicine, Hiroshima University, Hiroshima, Japan; 2 Department of Urology, Graduate School of Biomedical and Health Sciences, Hiroshima University, Hiroshima, Japan

**Keywords:** Angiotensin II, Angiotensin II type 1 receptor, Bladder cancer, Losartan, Implemental hypertension

## Abstract

The renin-angiotensin system (RAS) plays a central role in regulating blood pressure and has recently been implicated in cancer biology. Although angiotensin II (AngII) receptor blockers (ARBs) have shown clinical benefit in bladder cancer, their mechanisms of action remain unclear. Here, we investigated the contribution of AngII type 1 receptor (AGTR1) to bladder cancer progression and assessed the therapeutic potential of the ARB losartan (LOS). In patients with primary non-muscle-invasive bladder cancer, intravesical recurrence following transurethral tumor resection correlated with AGTR1 expression levels. Public database analysis revealed that the expression of AGTR1 and its downstream kinases, extracellular signal-regulated kinase (ERK) 1 and ERK2, was associated with overall survival in bladder urothelial carcinoma. In AGTR1-overexpressing T24 bladder cancer cells, AngII promoted invasion and migration and upregulated neuronal nitric oxide synthase, without affecting proliferation. These effects were accompanied by rapid ERK phosphorylation alongside Akt dephosphorylation. RNA sequencing revealed that AGTR1 expression and AngII stimulation activated NF-κB, mTOR, and epithelial-mesenchymal transition (EMT) pathways. LOS suppressed these AngII-mediated responses, whereas the AngII-independent upregulation of EMT-related proteins and the enhancement of mitochondrial energy metabolism by AngII in AGTR1-overexpressing cells remained unaffected. In vivo, AGTR1 facilitated early tumor engraftment and promoted tumor progression, accompanied by reduced E-cadherin and elevated N-cadherin expression, with most of these changes suppressed by LOS treatment. In conclusion, our findings highlight the crucial role of AGTR1 in bladder cancer and support the repositioning of ARBs, such as LOS, as therapeutics for AGTR1-upregulated bladder cancer, while underscoring the importance of AGTR1 stratification for future clinical evaluation.

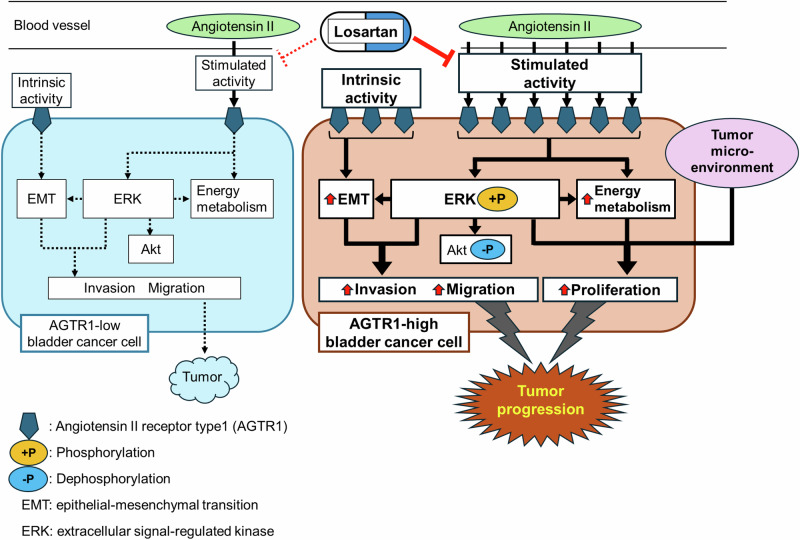

## Introduction

The renin-angiotensin system (RAS) plays a critical role in the regulation of blood pressure in humans [1]. Consequently, drugs targeting RAS, including angiotensin II type 1 receptor (AGTR1) blockers (ARBs), are widely used in the clinical management of hypertension. Like other components of metabolic syndrome, hypertension is recognized as a risk factor for the development and progression of various cancers [2]. Recently, the *AGTR1* gene, which encodes the AGTR1 protein, has gathered attention as a potential oncogene implicated in various cancer types, including liver cancer, breast cancer, ovarian cancer, and esophageal squamous cell carcinoma [3–8]. Several studies have suggested that ARBs may improve clinical outcomes in bladder cancer; however, since these observations are based on small cohorts or findings from in vitro experiments, the underlying mechanisms remain unclear [9–12].

Bladder cancer is the twelfth most common solid tumor, accounting for 500,000 new cases and 200,000 deaths annually worldwide [13, 14]. While treatments like transurethral resection of bladder tumor (TURBT) and Bacillus Calmette-Guérin (BCG) are effective for early-stage disease, advanced bladder cancer is typically managed with radical cystectomy and chemotherapy [15]. Recently, immune checkpoint inhibitors such as pembrolizumab and atezolizumab have shown promise, particularly in metastatic cases. However, these therapies are costly and can cause serious side effects [16]. Therefore, there is growing interest in developing safer and more affordable treatment options.

RAS and the use of ARBs have been shown to influence bladder function and mechanical strain [17]. While dysregulated AGTR1 signaling has been implicated in several cancers [3–8], its mechanistic role in bladder cancer and its potential as a therapeutic target of ARB treatment remain unclear. AGTR1 signaling activates multiple intracellular cascades, most notably extracellular signal-regulated kinase (ERK) and phosphoinositide 3-kinase (PI3K)/Akt pathways [18]. ERK signaling promotes cell proliferation, invasion, and migration, and consequently contributes to tumor progression through NF-κB activation and epithelial-mesenchymal transition (EMT) [19–21]. In contrast, PI3K/Akt signaling regulates cell survival and growth, partly through the mechanistic target of rapamycin 1 (mTORC1), and is associated with therapy resistance and metabolic reprogramming in cancer [22, 23]. These pathways exhibit reciprocal regulation and crosstalk, collectively driving malignant phenotypes.

In this study, we identified a correlation between AGTR1 expression and patient prognosis in bladder cancer. Ectopic expression of AGTR1 in bladder cancer cell lines conferred responsiveness to angiotensin II (AngII), enhancing invasion and migration capabilities, which were accompanied by activation of ERK and its related signaling pathways. These AGTR1/AngII-mediated oncogenic responses, as well as tumor progression in vivo, were effectively suppressed by the ARB losartan (LOS). ARBs may offer therapeutic benefit in bladder cancer patients exhibiting enhanced AGTR1 expression.

## Materials and methods

### Subjects

This study included patients who underwent TURBT for the first time at Hiroshima University Hospital between January 2016 and December 2018 and were diagnosed with non-muscle invasive bladder cancer. Patients with a follow-up period of at least 24 months were eligible for inclusion. Pathological diagnoses were performed based on the 1973 World Health Organization (WHO) classification. Exclusion criteria were as follows: cases with significant ablation artifacts, diagnosis of carcinoma in situ (CIS), and a history of upper tract urinary cancer (UTUC), due to challenges in accurate evaluation. After applying these criteria, 55 out of 138 patients were included in the final analysis. Relevant clinicopathological data, including age, sex, pathological T stage, tumor grade, pathological subtype, tumor number, and smoking history, history of hypertension, or use of antihypertensive medications, were extracted from medical records. Patient background characteristics and recurrence-free survival outcomes were also analyzed. This study was conducted following the Declaration of Helsinki and Good Clinical Practice guidelines. All experimental procedures were approved by the Ethics Committee of Hiroshima University Hospital (approval number E-326-2). All patients provided written informed consent before participation.

### Immunohistochemical staining

Immunohistochemical staining was conducted on tumor sections obtained by TURBT. Briefly, after the overnight incubation at 4 °C with anti-AGTR1 antibody (1:400; sc-515884; Santa Cruz Biotechnology, California, USA), the sections were washed with phosphate-buffered saline (PBS) containing 0.05% Tween-20. They were then incubated for 1 h at room temperature with anti-mouse HRP polymer (Vector Laboratories, California, USA), followed by a 30-min incubation with the VECTASTAIN Elite ABC kit (Vector Laboratories). Finally, the sections were treated with 3,3’-diaminobenzidine substrate buffer (FUJIFILM Wako Pure Chemical Corporation, Tokyo, Japan) for 5 min. AGTR1 expression in tumor lesions was evaluated using a conventional scoring system [24], based on both the proportion and intensity of positively stained cells. The percentage score was assigned as follows: 0 for 0–24%, 1 for 25–49%, 2 for 50–74%, and 3 for 75–100% of cells. The intensity score was graded as follows: 0 for none, 1 for weak, 2 for moderate, and 3 for strong staining. The total score, ranging from 0 to 6, was calculated by summing the percentage and intensity scores. Expression levels were classified as weak (total score 0–2) or strong (total score 3–6). Whenever feasible, three or more distinct tumor areas were assessed in each case.

### Cell culture

The human bladder cancer cell line T24 (RRID: CVCL_0554) was obtained from the Pathology Core of the Bladder Cancer SPORE at MD Anderson Cancer Center (Texas, USA). Cells were cultured in RPMI-1640 medium (Sigma-Aldrich, Missouri, USA) supplemented with 10% fetal bovine serum (FBS; Gibco, New York, USA) and 1% penicillin-streptomycin (FUJIFILM Wako Pure Chemical Corporation) at 37°C in a humidified atmosphere containing 5% CO2.

### Lentivirus infection

A human open-reading frame DNA sequence corresponding to the *AGTR1* gene (Darmacon, Idaho, USA) was cloned into a lentiviral plasmid, pCDH-MSCV-MCS-EF1-GFP-T2A-Puro (pCDH; RRID: Addgene_102325) to enable ectopic expression of AGTR1 in T24 cells. The empty pCDH vector was used as a negative control. The *AGTR1* sequence, fused with a C-terminal Flag-tag, was verified by sequencing prior to use. Lentiviral particles were generated by co-transfecting Lenti-X 293 T cells (Takara, Kyoto, Japan) with the AGTR1-expressing or control plasmid and three packaging plasmids: PMDLg./pRRE pRSV-Rev (RRID: Addgene_12253), and pVSV-G (RRID: Addgene_138479). Transfection was facilitated using Lipofectamine 2000 (Thermo Fisher Scientific, Massachusetts, USA). After 48 h, viral supernatants were harvested, filtered, and used to infect target cells overnight. Infection efficiency exceeded 90%, as confirmed by GFP expression via flow cytometry. The resulting control and AGTR1-overexpressing cells were subjected to subsequent in vitro analyses in the presence or absence of angiotensin II (AngII; Sigma-Aldrich), losartan (LOS; Tokyo Chemical Industry Co., Ltd., Tokyo, Japan), candesartan (Tokyo Chemical Industry Co., Ltd.), or telmisartan (FUJIFILM Wako Pure Chemical Corporation).

### Western blot analysis

Western blot analysis was performed as previously described [25]. Briefly, protein samples were electrophoresed on 5–20% precast polyacrylamide gels (SuperSep Ace, FUJIFILM Wako Pure Chemical Corporation) and transferred to nitrocellulose membranes (GE Healthcare Life Sciences, Illinois, USA) by electroblotting. Membranes were blocked with 5% nonfat dry milk in TBST buffer (10 mM Tris, pH 8.0, 150 mM NaCl, 0.05% Tween-20) for 30 min and incubated overnight at 4 °C with primary antibodies. The following primary antibodies we used: anti-AGTR1 (1:1000, sc-515884; Santa Cruz Biotechonology), -vimentin (1:1000, #5741; Cell Signaling Technology, Massachusetts, USA), -E-cadherin (1:10000, #20874-1-AP; Proteintech, Shanghai, China), -N-cadherin (1:2000, #22018-1-AP; Proteintech), -Flag (1:1000, #.8146; Cell Signaling), -eNOS (1:1000, #5880; Cell Signaling), -nNOS (1:1000, #4234; Cell Signaling), -P44/42 MAPK (1:1000, #4695; Cell Signaling), -phospho-p44/42 MAPK (1:2000, #4370 Cell Signaling), -Akt (1:1000, #4691; Cell Signaling), -phospho-Akt (1:1000, #4060 Cell Signaling), -STAT5 (1:1000, #9363; Cell Signaling), -phospho-STAT5 (1:1000, # 4322 Cell Signaling), -PI3K (1:1000, # 4292 Cell Signaling), phospho-PI3K (1:1000 # 4228 Cell Signaling) and -β-actin 1:2000, #A2228; Sigma-Aldrich). For ERK and Akt quantification, band intensities were measured using ImageJ software (https://imagej.nih.gov/ij/) to calculate the p‑ERK/ERK and p-Akt/Akt ratio. Cells were pre‑incubated with LOS (10 μM) for 1 h, followed by AngII stimulation for 5–60 min.

### Transwell invasion assay

Transwell invasion assays were performed using 8.0 μm pore size ThinCert membranes (Greiner Bio-One, Kremsmünster, Austria) and 24-well cell culture inserts (Corning, New York, USA). Serum-containing medium (10% FBS) was added to the lower chamber, while cells suspended in serum-free medium were seeded into the upper chamber. After 24 h of incubation, cells that had invaded through the underside of the membrane were fixed, stained using the Differential Quik Ⅲ Stain Kit (Polysciences, Warrington, PA, USA), and counted under a microscope.

### Wound healing assay

Wound healing assays were performed using ibidi culture inserts (ibidi culture-insert 2 well; ibidi GmbH, Munich, Germany). Cells were seeded into each insert at a density of 5.0 × 10^5^ cells/ml and allowed to adhere overnight. After removal of the inserts, cells were incubated in fresh medium for 6 h. Images of the same wound area were captured at the beginning and end of the incubation period using a light microscope at ×100 magnification. The migration activity of the cells was quantified by measuring the distance between the wound edges using ImageJ software (https://imagej.net/ij/). All experiments were conducted in triplicate under identical conditions.

### Cell proliferation assay

Cells were seeded into 96-well plates at a density of 1.0 × 10^4^ cells/ml and cultured for 48 to 72 h. Cell proliferation was assessed using the Premix WST-1 Cell Proliferation Assay System (Takara), and absorbance was measured at 450 nm using a microplate reader. Control wells containing untreated cells and blank wells containing medium without cells were included as experimental controls and blanks, respectively.

### Extracellular oxygen consumption rate and acidification assays

The oxygen consumption rate (OCR) and extracellular acidification rate (ECAR) were evaluated using the Seahorse XF Pro Analyzer (Agilent Technologies, Santa Clara, CA, USA), following the manufacturer’s protocols. Assays were performed using the Seahorse XF Mito Stress Test Kit (Agilent Technologies). Briefly, cells were seeded at a density of 1.5 × 10^4^ cells per well in a Seahorse XF pro M cell culture microplate and incubated overnight. Baseline measurements were recorded immediately following AngII treatment, with or without prior incubation with losartan (LOS) for 1 h. Subsequently, the following compounds were sequentially injected: oligomycin (an ATP synthase inhibitor), p-trifluoromethoxy carbonyl cyanide phenylhydrazone (FCCP; a mitochondrial uncoupler), and a combination of rotenone (Rote; a complex I inhibitor) and antimycin (AA; a complex III inhibitor). The data were analyzed using Seahorse Wave Pro Software (Agilent Technologies). Maximum OCR, measured after FCCP addition, and basal OCR, recorded before prior to oligomycin treatment, were expressed as pmol/minute/10^3^ cells. Maximum ECAR, measured after rotenone addition, and basal ECAR, recorded before oligomycin treatment, were expressed as mpH/minute/ 10^3^ cells.

### RNA sequencing (RNA-seq)

Total RNA was extracted from cells using the RNeasy Plus Mini Kit (Qiagen, Hannover, Germany). Subsequently, RNA-seq was performed by a commercial provider (Service ID: F25A420000153_HOMidxlR; BGI, Huada Gene, Wuhan, China). Briefly, mRNA was enriched from total RNA using oligo (dT) magnetic beads. Double-stranded cDNA was synthesized and amplified via PCR using specific primers. Sequencing was conducted using DNA Nanoball sequencing (DNB-seq), with the Homo sapiens reference genome assembly GCF_000001405.40 (GRCh38.p14) obtained from NCBI serving as the reference. Pathway enrichment analysis and gene set enrichment analysis (GSEA) were conducted using the Dr. TOM data mining system developed by BGI. The Hallmark gene sets from the Molecular Signatures Database (MSigDB) were used as the reference for GSEA. Differential gene expression was presented as log2 fold change, indicating either upregulation or downregulation.

### Tumor implantation

All animal experiments were performed in strict accordance with the “Guidelines for the Care and Use of Laboratory Animals” issued by the Hiroshima University Animal Experiment Committee (Permit No. 29-58). For the xenograft model, AGTR1-overexpressing T-24 cells and control virus-infected T-24 cells were prepared at a concentration of 5.0 × 10^6^ cells in a 1:1 mixture of 50 μl of Hank’s Balanced Salt Solution (Gibco) and 50 μl Matrigel (Corning, Corning, NY, USA). The cell suspensions were subcutaneously injected into 6-week-old male BALB/c-nu/nu mice (The Jackson Laboratory Japan, Yokohama, Japan). Estimated tumor volume was measured weekly using calipers and calculated using the formula: (width^2^ × length)/2. Once the estimated tumor volume stabilized at or above 50 mm³, LOS was administered via drinking water at a concentration of 0.6 g/l for 6 weeks, or withheld in control groups, following the previously described protocol [26, 27]. The tumor growth rate was calculated relative to the initiation of LOS treatment. At the end of the treatment period, mice were euthanized, and tumors were harvested, weighed, and stored at -80°C until subsequent analysis by Western blotting.

### In silico analysis

Transcriptomic data from TCGA were analyzed using the Gene Expression Profiling Interactive Analysis (GEPIA) platform (gepia.cancer-pku.cn), developed by Tang et al. [28]. A cohort of 400 patients with bladder urothelial carcinoma (BLCA) was stratified into AGTR1-high and -low expression groups based on the median expression value. Kaplan–Meier analysis was then performed to evaluate differences in overall survival between the two groups. Similarly, 402 BLCA patients were classified into high and low expression groups for ERK1 and ERK2, respectively, using the same median cutoff approach. Kaplan–Meier analysis was conducted to assess the association between ERK1/2 expression levels and overall survival outcomes.

### Statistical analysis

Data are expressed as means ± standard error of the mean (SEM). Multiple group comparisons were performed using one-way analysis of variance (ANOVA) followed by Dunnett’s multiple comparison test. Statistical analysis was conducted using JMP software (SAS Institute Inc.; RRID: SCR_008567). Welch’s *t*-test, log-rank test, or Pearson’s chi-squared test was applied for comparison between the two groups. Statistical significance was defined as a *p*-value less than 0.05. For GSEA, false discovery rate-adjusted *p*-values (*q*-values) were also calculated using the Dr. TOM platform, as previously described [29], to account for potential false positives.

## Results

### Correlation of AGTR1 expression with bladder cancer prognosis

**Fig. 1:**
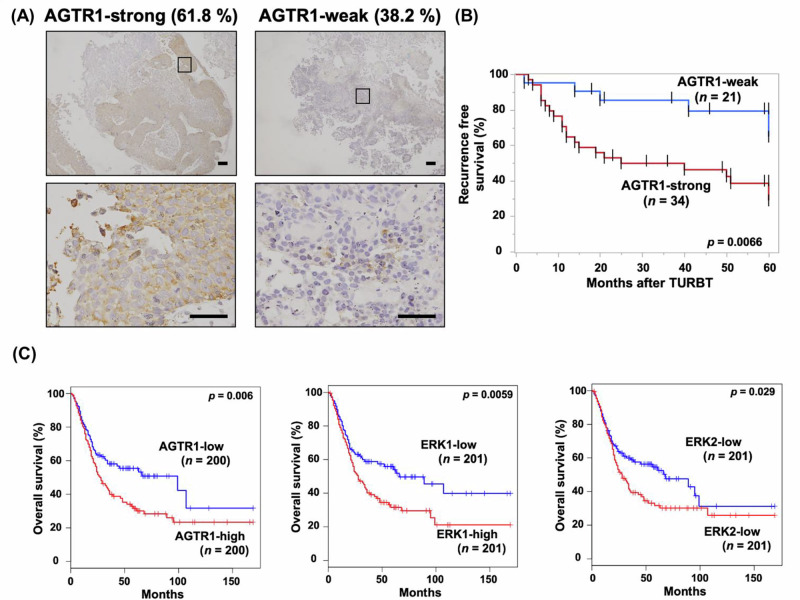
Correlation of angiotensin II type 1 receptor (AGTR1) expression with bladder cancer prognosis. **A** Representative immunohistochemical staining of AGTR1-strong and AGTR1-weak tumor specimens. Among 55 cases, 34 (61.8%) exhibited strong AGTR1 expression and 21 (38.2%) showed weak expression. Images are presented at original magnifications of ×40 (upper panels, scale bar: 250 μm), with corresponding high-magnification views (×400) of the boxed regions displayed below (scale bar: 100 μm). **B** Kaplan–Meier plots of recurrence-free survival for patients with AGTR1-strong versus AGTR1-weak tumors following transurethral resection of bladder tumor. **C** Kaplan–Meier plots of overall survival for bladder cancer patients with high versus low AGTR1 (left), ERK1 (middle), and ERK2 (right) mRNA expression levels, based on data from the Gene Expression Profiling Interactive Analysis database. *p* values were calculated using the log-rank test. ERK extracellular signal-regulated kinase

We analyzed AGTR1 expression in tumor specimens from patients with non-muscle invasive bladder cancer (NMIBC) who underwent TURBT at Hiroshima University Hospital. Using the immunohistochemical scoring system established by Shirotake et al. [24], 34 (61.8%) and 21 (38.2%) of 55 cases exhibited strong and weak AGTR1 expression, respectively. AGTR1 was predominantly localized to the cytoplasm and cell membrane of tumor cells (Fig. 1A). No significant associations were observed between AGTR1 expression levels and patient age, sex, pathological T stage, tumor grade, pathological atypia, smoking history, history of hypertension, or use of antihypertensive medications in our NMIBC cohort (Supplementary Table 1). Although patients with strong AGTR1 expression tended to present with multiple tumors at initial diagnosis, this trend did not reach statistical significance. Kaplan–Meier survival analysis revealed that patients with strong AGTR1 expression had significantly shorter recurrence-free survival compared to those with weak expression (Fig. 1B). Due to the limited cohort size, the influence of ARB use on clinical outcomes could not be conclusively determined.

To further evaluate the prognostic significance of AGTR1, we analyzed overall survival data from bladder cancer patients using the publicly available GEPIA database. Patients with elevated AGTR1 mRNA expression exhibited significantly reduced survival compared to those with lower expression levels (*p* = 0.006, Fig. 1C). AGTR1 signaling is known to activate mitogen-activated protein kinase (MAPK) pathways, including ERK [18]. In the same dataset, expression levels of ERK1 and ERK2 were also associated with overall survival (*p* = 0.0059 and *p* = 0.029, respectively; Fig. 1C). These findings suggest that AGTR1 may influence bladder cancer prognosis through its downstream signaling cascade.

### AGTR1 promotes AngII-mediated invasion and migration of bladder cancer cells in vitro

**Fig. 2:**
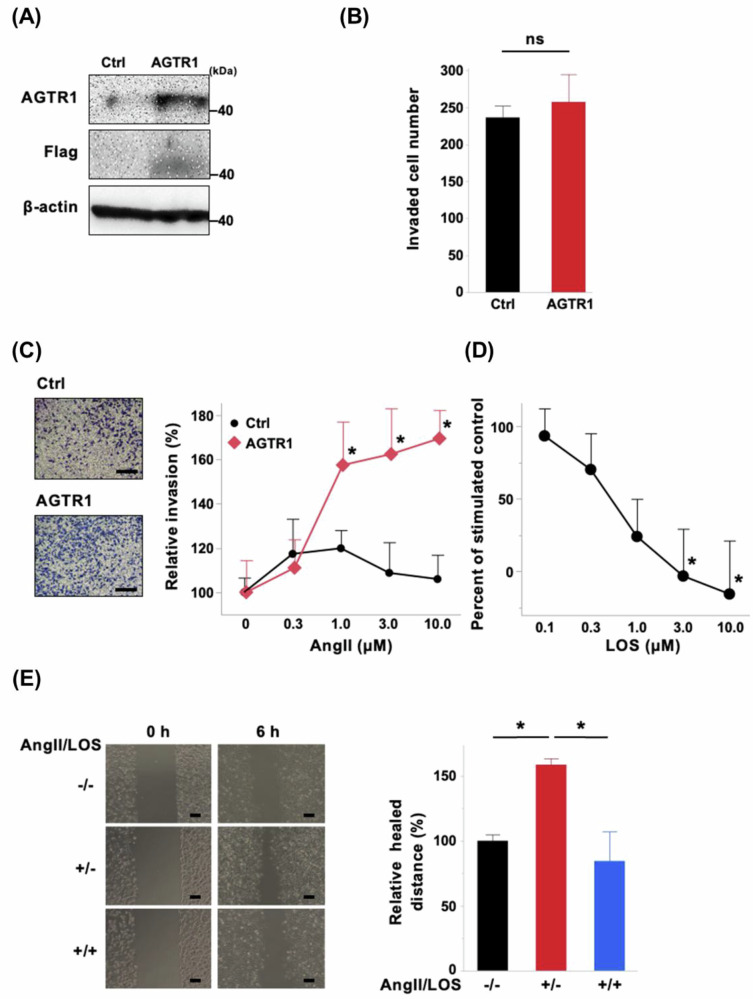
Contribution of angiotensin II (AngII) type 1 receptor (AGTR1)/AngII signaling to bladder cancer cell activity in vitro. **A** Western blot analysis of AGTR1*-*overexpressing (AGTR1) and control (Ctrl) T24 cells. Representative results from three independent experiments are shown. **B** Transwell invasion assay comparing AGTR1 and Ctrl cells under basal conditions. Data are presented as mean ± standard error of the mean (SEM) of invaded cell counts (*n* = 3). **C** Transwell invasion assay of AGTR1 and Ctrl cells treated with varying concentrations of AngII. Data are presented as mean ± SEM of relative invasion compared to unstimulated cells (*n* = 3). *p  <  0.05, compared to Ctrl by one-way analysis of variance (ANOVA) with Dunnett’s multiple comparison test. Representative Diff-Quick-stained transwell membranes treated with 10 μM AngII are shown in the left panels. Scale bar: 100 μm. **D** Transwell invasion assay of AGTR1 cells treated with 10 μM AngII and varying concentrations of losartan (LOS). Data are presented as mean ± SEM of percent invasion relative to AngII-stimulated control (*n* = 3). *p  <  0.05, compared with AngII-stimulated control by one-way ANOVA with Dunnett’s multiple comparison test. **E** Wound healing assay of AGTR1 cells treated with 10 μM AngII in the presence or absence of 3 μM LOS. Data are presented as mean ± SEM of the relative healed distance compared to unstimulated control (*n* = 3). *p  <  0.05, compared with AngII-stimulated control by one-way ANOVA with Dunnett’s multiple comparison test. (*n* = 3). Representative images of migrated cells are shown in the left panels. Scale bar: 100 μm. ns not significant

To elucidate the functional significance of AGTR1 in bladder cancer, we assessed its impact on cell invasion, migration, and proliferation by overexpressing AGTR1 in T24, a well-characterized bladder cancer cell line with low basal AGTR1 expression. Lentiviral transduction of an AGTR1-overexpression cassette successfully enhanced AGTR1 levels, as confirmed by immunoblotting for AGTR1 and Flag-tag (Fig. 2A). Under basal conditions, AGTR1*-*overexpressing cells did not exhibit significant changes in invasive activity compared to control cells (Fig. 2B). However, stimulation with AngII dose-dependently increased invasion in AGTR1-overexpressing cells, while control cells remained unaffected (Fig. 2C). This AngII-mediated invasion was effectively inhibited by the ARB LOS, with statistically significant suppression observed at concentrations above 3 μM, close to the sub-micromolar levels achieved in clinical use (Fig. 2D), as well as by other ARBs, candesartan and telmisartan (Supplementary Fig. 1), in a dose-dependent manner.

We further evaluated the migration activity of AGTR1-overexpressing T24 bladder cancer cells. Under basal conditions, no significant difference in migration was observed between AGTR1-overexpressing and control cells (Supplementary Fig. 2A). However, treatment with AngII significantly increased migration in AGTR1-overexpressing cells, an effect that was inhibited by LOS (Fig. 2E). Migration in control cells remained unaffected by AngII stimulation (Supplementary Fig. 2B).

In contrast, cell proliferation was not affected by AGTR1 overexpression, AngII stimulation, or LOS treatment (Supplementary Fig. 2C). Interestingly, telmisartan at 10 μM, a concentration comparable to that used for invasion assays, slightly but significantly inhibited the proliferation of AGTR1-overexpressing T24 cells; however, candesartan showed no such effect (Supplementary Fig. 3). These findings suggest that AGTR1 overexpression primarily enhances the invasive and migratory capabilities of T24 bladder cancer cells in response to AngII, rather than their proliferative activity.

### AGTR1 activates ERK signaling upon Angiotensin II stimulation in bladder cancer cells

**Fig. 3:**
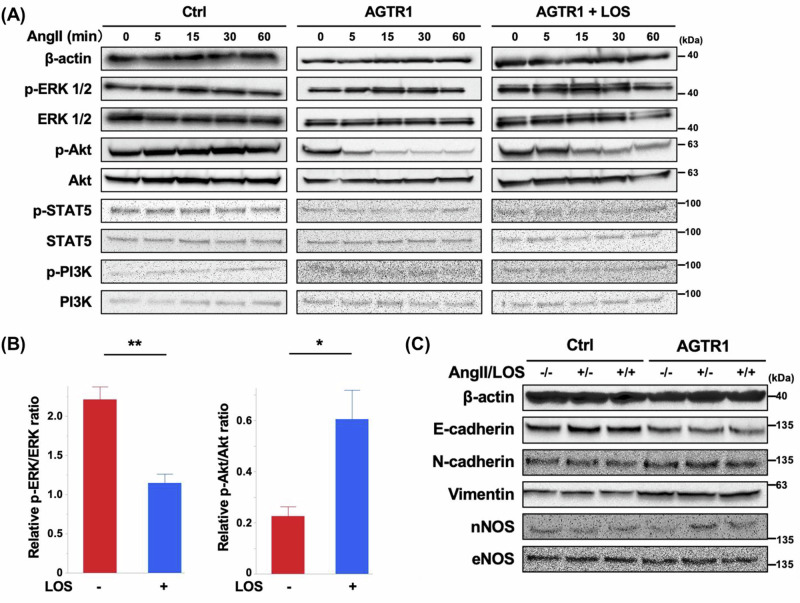
Contribution of angiotensin II (AngII) type 1 receptor (AGTR1) on protein phosphorylation and expression in bladder cancer cells. **A** Western blot analysis of control T24 cells (Ctrl) and AGTR1-overexpressing (AGTR1) T24 cells following stimulation with 10 μM AngII for 5–60 min, in the presence or absence of 10 μM losartan (LOS). Phosphorylation dynamics and total expression levels of extracellular signal-regulated kinase (ERK), Akt, signal transducer and activator of transcription (STAT5), and phosphoinositide 3-kinase (PI3K) were evaluated. β-actin was used as a loading control. Representative results from three independent experiments are shown. **B** Relative ratios of p-ERK/ERK (left) and p-Akt/Akt (right) were calculated at 15 min after AngII stimulation compared to pre-stimulation levels, in the presence and absence of 10 μM LOS (*n* = 3). **C** AGTR1 and Ctrl cells were treated with or without 10 μM AngII in the presence or absence of LOS for 24 h. Western blot analysis was performed to evaluate the expression of the indicated proteins. Representative results from three independent experiments are shown. nNOS neuronal nitric oxide synthase (NOS), eNOS endothelial neuronal nitric oxide synthase

AGTR1 has been implicated in cancer progression, and its activation has been associated with the ERK signaling pathway [4, 6, 7]. To investigate this mechanism in bladder cancer, we performed western blot analysis of phosphorylated proteins in AGTR1-overexpressing and control T24 cells. Upon AngII stimulation, AGTR1-overexpressing cells exhibited an increase in ERK phosphorylation and a concurrent decrease in Akt phosphorylation. This response peaked approximately at 15 min after AngII treatment, whereas control cells showed no such changes throughout the observation period (Fig. 3A). Although STAT5 has been proposed as a potential downstream target of AGTR1 signaling [30], AngII stimulation induced only minimal STAT5 phosphorylation in both AGTR1-overexpressing and control cells. As shown in Fig. 3A–B, the AngII-induced phosphorylation of ERK and dephosphorylation of Akt were significantly suppressed by LOS. However, no change in PI3K, an upstream regulator of Akt, was observed under any condition, with or without AngII stimulation or LOS treatment (Fig. 3A).

We next investigated the impact of AGTR1 expression and AngII stimulation on the expression of molecules associated with cancer progression in T24 cells. Consistent with the proposed role in promoting EMT [31], AGTR1 overexpression led to a modest increase in vimentin and N-cadherin levels, accompanied by a reduction in E-cadherin expression (Fig. 3C). These EMT-related changes were not further influenced by AngII stimulation or LOS treatment. We also assessed the expression of neuronal nitric oxide synthase (nNOS) and endothelial NOS (eNOS), which are known to promote angiogenesis and contribute to cancer cell proliferation and migration [32]. AGTR1 overexpression alone did not affect nNOS or eNOS levels. However, unlike the EMT-related changes, AngII stimulation selectively upregulated nNOS expression in AGTR1-overexpressing cells, an effect that was attenuated by LOS treatment (Fig. 3C). eNOS expression remained unchanged under all conditions. These findings suggest that AGTR1-overexpression predisposes bladder cancer cells to EMT-like changes and enhances their sensitivity to AngII, promoting ERK phosphorylation and nNOS induction.

### Gene expression alteration in AGTR1-overexpressing bladder cancer cells by AngII and LOS

**Fig. 4:**
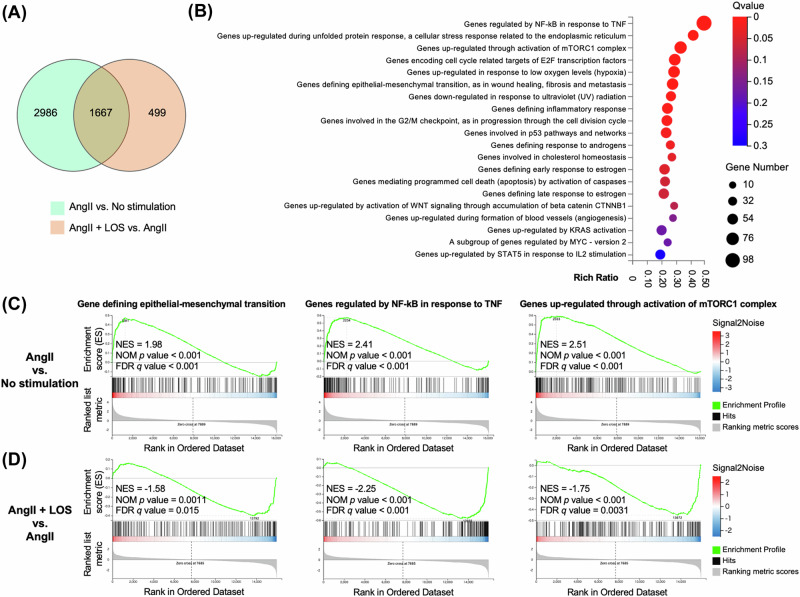
Gene expression change associated with angiotensin II (Ang II) type 1 receptor (AGTR1) and modulation by AngII and losartan (LOS). **A** Venn diagram illustrating the number of differentially expressed genes in AGTR1-overexpressing T24 cells (AGTR1) under two conditions: AngII stimulation versus (vs) no stimulation (AngII vs. No stimulation), and combined AngII and LOS treatment versus AngII alone (AngII + LOS vs. AngII), with a false discovery rate (FDR) *q* < 0.05). **B** Bubble plots showing the top 20 hallmark gene sets commonly enriched in both comparisons from panel **A**. Enrichment patterns for indicated gene sets comparing AngII vs. No stimulation (**C**) and AngII + LOS vs. AngII (**D**). The corresponding normalized enrichment scores (NES), nominal (NOM) *p* values, and FDR *q* values are also shown. TNF Tumor necrosis factor, mTORC1 mechanistic target of rapamycin1

To evaluate the molecular pathways associated with AGTR1-overexpression, RNA sequencing analysis was performed on four groups: control T24 cancer cells without drug treatment (Ctrl), AGTR1-overexpressing T24 cells without drug treatment (AGTR1), AGTR1 treated with AngII, and AGTR1 treated with both AngII and LOS. Three comparison groups were analyzed: (1) AGTR1 versus Ctrl, (2) AGTR1 treated with AngII versus untreated AGTR1 (No stimulation), and (3) AGTR1 treated with both AngII and LOS versus AngII alone. Gene set enrichment analysis identified 1667 commonly differentially expressed genes across two comparisons: AngII versus no stimulation and AngII and LOS versus AngII alone (Fig. 4A and Supplementary Table 2). These genes were classified according to the Hallmark gene sets, and the top 20 pathways were highlighted (Fig. 4B and Supplementary Table 3). Notably, pathways related to genes defining epithelial-mesenchymal transition (EMT), genes regulated by NF-κB in response to tumor necrosis factor (TNF), and genes upregulated through activation of the mTORC1 complex were significantly affected. Specifically, the administration of AngII markedly promoted these pathways in AGTR1-overexpressing T24 cells (Fig. 4C), whereas treatment with LOS significantly suppressed them (Fig. 4D). In particular, EMT was significantly associated with the AGTR1-overexpression itself. Although NF-κB and mTORC1-related pathways were also enriched in AGTR1-overexpressing cells, their association did not reach statistical significance (Supplementary Fig. 4 and Supplementary Tables 4 and 5).

### AngII/AGTR1 signal enhances mitochondrial energy metabolism in bladder cancer cells

**Fig. 5:**
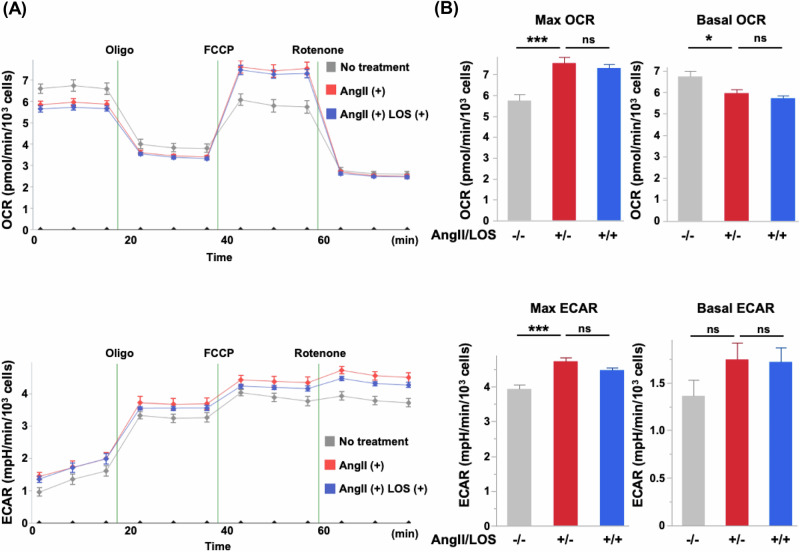
Contribution of angiotensin II (AngII) type 1 receptor (AGTR1)/AngII signal on cellular metabolic activity in bladder cancer cells. **A** Time course of oxygen consumption rate (OCR, upper panel) and extracellular acidification rates (ECAR, lower panel) in AGTR1-overexpressing T24 cells treated with (+) or without (−) 10 μM AngII in the presence or absence of 3 μM losartan (LOS). The timing of injecting oligomycin (Oligo), p-trifluoromethoxy carbonyl cyanide phenylhydrazone (FCCP), and rotenone is indicated. **B** Maximum (Max) and basal OCR (upper panels) and ECAR (lower panels) with or without AngII stimulation in the presence or absence of 3 μM LOS. Data are presented as mean ± standard error of the mean (SEM) of OCR and ECAR. *p  <  0.05, compared with AngII-stimulated control (+/−) by one-way analysis of variance (ANOVA) with Dunnett’s multiple comparison test. ns not significant

To further clarify the mechanisms by which LOS influences AGTR1-mediated responses in bladder cancer cells, we next analyzed the impact of AngII stimulation on mitochondrial energy metabolism in AGTR1-overexpressing T24 cells. AngII treatment increased the OCR, a marker of mitochondrial respiratory capacity, while slightly reducing basal OCR levels (Fig. 5A–B). In addition, the ECAR, indicative of glycolytic activity, was modestly elevated in AGTR1-overexpressing cells (Fig. 5A–B). In contrast, control cells showed no notable metabolic responses to AngII stimulation (Supplementary Fig. 5). Notably, these AngII-induced metabolic changes in AGTR1-overexpressing cells were not suppressed by LOS treatment (Fig. 5B). These findings suggest that AngII/AGTR1 signal promotes both respiratory capacity and glycolytic activity in bladder cancer cells, and that these metabolic enhancements are not effectively targeted by LOS, indicating a potential bypass of its inhibitory mechanism.

### AGTR1 encourages tumor progression in vivo

**Fig. 6:**
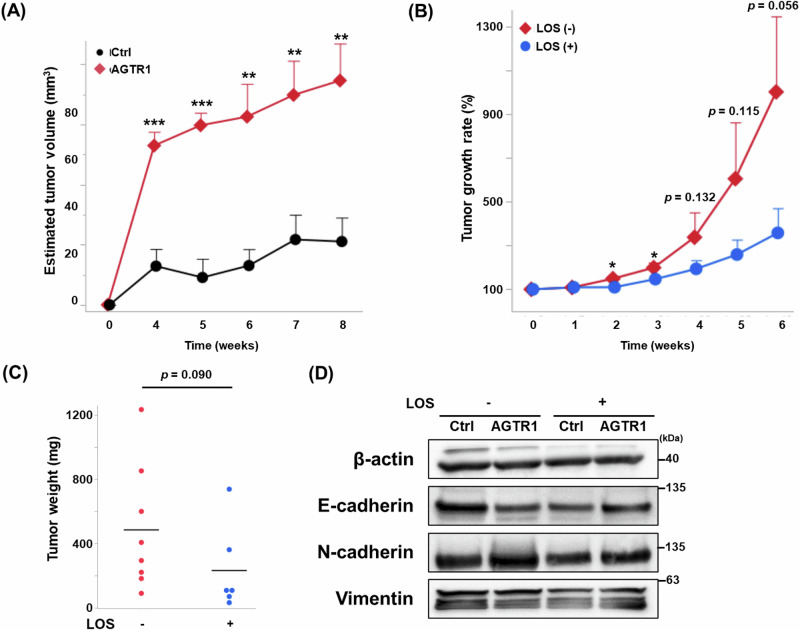
Effect of losartan (LOS) on angiotensin II (AngII) type 1 receptor (AGTR1)-mediated bladder cancer progression. **A** Estimated tumor volume of the xenograft model with AGTR1-overexpressing (AGTR1) and control (Ctrl) T24 cells. Data are presented as mean ± standard error of the mean (SEM) (*n* = 5). **p < 0.01 and ***p  <  0.001, compared with Ctrl by Welch’s *t*-test. **B** Tumor growth rate of AGTR1 treated with or without LOS. Data are presented as mean ± SEM of estimated tumor volume relative to the initiation of LOS treatment (*n* = 4–8). *p  <  0.05 and individual *p*-values are indicated, compared to LOS-untreated control by Welch’s *t*-test. **C** Weight of harvested tumors with or without LOS treatment (*n* = 6–8). The *p*-value determined by Welch’s *t*-test is indicated. **D** Western blot analysis of Ctrl and AGTR1 tumors with or without LOS. Representative results from three independent samples are shown. ns not significant

To investigate the role of AGTR1 in vivo, we established a xenograft model by subcutaneously transplanting AGTR1-overexpressing T24 bladder cancer cells (AGTR1) and control T24 cells (Ctrl) into BALB-c/nu/nu mice. Tumors became macroscopically detectable and stabilized approximately four weeks after transplantation. Over the 8-week observation period, the estimated tumor volume in the AGTR1 group was significantly larger than that in the Ctrl group (Fig. 6A). Subsequently, we evaluated the therapeutic effect of LOS on further tumor progression. Tumors in the LOS-treated group were significantly smaller, particularly at 2 and 3 weeks following the initiation of LOS administration (Fig. 6B). However, no statistically significant differences in estimated tumor volume or tumor weight were observed at the final sampling point (*p* = 0.056 or *p* = 0.090, respectively; Fig. 6B–C). As shown in Supplementary Fig. 6, two of the six LOS-treated mice developed large cyst-like changes within tumors, suggestive of necrosis, which complicated accurate in vivo tumor volume measurements; these two cases were excluded from Fig. 6B. Water intake, body weight, and other phenotypic parameters were not affected by LOS treatment (data not shown). To evaluate EMT-related markers, as well as the expression and phosphorylation of ERK and Akt, representative tumor samples were analyzed by western blot. AGTR1 overexpression was associated with reduced E-cadherin and elevated N-cadherin levels, while vimentin expression remained unchanged. Notably, LOS treatment partially restored E-cadherin expression and suppressed N-cadherin upregulation in AGTR1-overexpressing tumors (Fig. 6D). Together with RNA-seq data, these findings suggest that LOS administration may inhibit the EMT-related pathway in AGTR1-overexpressing bladder cancer cell lines, thereby potentially suppressing tumor progression. Consistent with its in vitro property, phosphorylation of ERK showed a decreasing trend in mice transplanted with AGTR1-overexpressing tumors and treated with LOS, compared with untreated controls. Notably, the expression but not phosphorylation of Akt was significantly suppressed by LOS (Supplementary Fig. 7).

## Discussion

In this study, we demonstrated that the prognosis of bladder cancer patients is associated with the expression level of AGTR1. Consistently, bladder cancer cells overexpressing AGTR1 exhibited enhanced AngII-induced invasion and migration in vitro, as well as accelerated tumor progression in vivo. These phenotypes and AGTR1-mediated activation of ERK signaling, EMT-, NF-κB-, and mTORC1-related pathways were suppressed by LOS. Our findings suggest the potential of ARBs, such as LOS, as novel therapeutic agents for bladder cancer.

We found that patients with high AGTR1 expression had significantly lower recurrence-free survival following TURBT. This association was further corroborated by analysis of the GEPIA public database, where stratification into AGTR1-high and -low expression groups revealed similar trends. These findings are consistent with recent results reported by Mei et al. [33], despite the use of a different stratification strategy in that study. Furthermore, we demonstrated that poor prognosis in BLCA patients is also linked to elevated expression of ERK, a downstream effector of AGTR1 signaling. The association of AGTR1 expression with promoting tumor progression has also been reported in other cancers, such as breast cancer [3–5, 33]. However, a few studies have suggested a favorable prognostic role of AGTR1 in certain cancer types [34], highlighting the complexity and context-dependence of AGTR1 signaling in cancer biology. Our present findings suggest that AGTR1 expression may serve as a useful biomarker for identifying patients who could benefit from ARB-based therapy, representing a potential contribution to personalized medicine.

In our in vitro model using AGTR1-overexpressing T24 bladder cancer cells, enhanced responsiveness to AngII stimulation promoted cancer cell invasion and migration. Treatment with three ARBs, LOS, telmisartan, and candesartan, suppressed AngII-induced invasion. Among these, telmisartan inhibited cell proliferation, a property not shared by LOS or candesartan, suggesting pharmacological differences that may influence mitochondrial metabolism. Telmisartan has uniquely been reported to suppress bladder cancer cell proliferation through activation of peroxisome proliferator-activated receptor γ [9], which regulates lipid metabolism, cell cycle, and anti-inflammatory pathways [35–37]. Although the role of this activity in bladder cancer remains controversial and warrants further investigation, such differences should be considered when selecting ARBs for potential cancer therapy.

We also revealed multiple regulatory roles of AGTR1 signaling. Specifically, AngII stimulation enhanced ERK phosphorylation while reducing Akt phosphorylation, indicating differential modulation of key signaling pathways. These findings are consistent with previous studies reporting ERK inactivation following AGTR1 downregulation in prostate and hepatocellular carcinoma cells [7, 38]. The ERK pathway is known to regulate both proliferation and senescence across various cell types, including cancer cells, and to contribute to the enhancement of cell invasion and migration abilities [19, 20]. In cancer, ERK signaling contributes to tumor progression by promoting the degradation of I-κB, an inhibitory protein that suppresses NF-κB activity. The resulting activation of NF-κB leads to the inhibition of apoptosis, acceleration of the cell cycle, increased production of inflammatory cytokines, and increased angiogenesis, processes that collectively drive tumor development and malignancy [21].

In contrast, the observed Akt dephosphorylation by AGTR1 stimulation appears contradictory to previous findings, at least in the cardiovascular system, where AGTR1 stimulation typically promotes Akt phosphorylation [22]. The PI3K/Akt pathway plays a crucial role in regulating cell survival and proliferation, both in normal and cancerous tissues. In the heart, this leads to angiogenesis and hypertrophy [22], while in cancer, it contributes to malignancy and resistance to therapy by regulating tumor proliferation, invasion, and migration [23, 39]. Therefore, inhibition of Akt signaling has been a major focus in cancer treatment strategies. AngII stimulation enhanced the invasion and migration of AGTR1-overexpressing T24 cells, but did not promote their proliferation, suggesting that ERK-driven proliferative signaling may have been counteracted by the downregulation of Akt activity. Several mechanisms have been proposed for Akt dephosphorylation, including PH-domain leucine-rich repeat protein phosphatase and the inactivation of its upstream kinases, such as phosphatidylinositol-3-kinase [40]. These mechanisms, particularly those affecting Ser473, detected by the antibody used in this study, may underlie the observed Akt suppression. Recent studies have highlighted reciprocal regulation between ERK and Akt pathways, where inhibition of one enhances the other [41]. Given AGTR1-driven ERK activation in our model, the observed Akt dephosphorylation likely represents a secondary, compensatory effect rather than a primary event. This interpretation supports our finding that AngII enhanced invasion and migration without promoting proliferation, indicating ERK dominance under these conditions. Given that mTORC1, a downstream effector of Akt signaling and a pathway implicated in cancer progression [18], was positively associated with AGTR1/AngII, the ERK-Akt interplay likely represents a key determinant of tumor cell phenotypes and merits exploration as a therapeutic target. In vivo, despite LOS-mediated downregulation of ERK phosphorylation, Akt did not exhibit a consistent trend with the in vitro results; instead, total Akt expression, but not its phosphorylation, was reduced. This variability suggests that ERK and Akt signaling may be shaped by multiple factors, including the tumor microenvironment, and that their activation is likely transient and highly responsive to AngII, as observed in vitro. Such context-dependent and dynamic regulation underscores the complexity of AGTR1-driven signaling and highlights the need for temporal analysis in future studies.

AGTR1-dependent tumor progression observed in vivo, despite the absence of proliferation enhancement in vitro, can be attributed, at least in part, to the activation of the EMT pathway [42]. Although nNOS, downstream of AGTR1, has been implicated in EMT through nitric oxide production in complex biological contexts, such as the vascular system [43, 44], our data revealed no direct association between eNOS or nNOS expression and EMT markers in vitro. Interestingly, EMT-related protein expression and transcriptomic alterations were observed in AGTR1-overexpressing bladder cancer cells even in the absence of AngII stimulation, suggesting a role for the intrinsic activity of AGTR1. Supporting this, Ekambaram et al. demonstrated that AGTR1 overexpression in breast cancer cells induces both ligand-dependent and -independent activation of NF-κB, through the CARMA1–Bcl10–MALT1 signalosome pathway [45]. Although not statistically significant, NF-κB signaling was enriched in AGTR1-overexpressing cells compared to Ctrl cells, even without AngII stimulation in our study as well. In hepatocellular carcinoma cells with substantial AGTR1 expression, Wang et al. reported that AGTR1 knockdown led to reduced proliferative capacity, G2-M phase arrest, increased p53 and p21 expression, and senescence-associated phenotypes [7]. However, their findings contrast with our results, which showed no significant impact of AGTR1 on the proliferative activity of bladder cancer cells in vitro. Moreover, while Wang et al. observed altered ERK phosphorylation following AGTR1 knockdown, our data indicate that ERK phosphorylation requires AngII stimulation and is not induced by AGTR1 overexpression alone. Although differences in cancer types and underlying molecular mechanisms warrant further examination, our findings propose that AGTR1-mediated tumor progression involves at least two distinct signaling pathways: one in which AngII stimulation induces ERK phosphorylation, and another in which AGTR1 intrinsically promotes EMT. The EMT activity is further enhanced by AngII stimulation, as indicated by RNA-seq analysis. This dual mechanism is further supported by the absence of AngII-dependent effects on in vitro proliferation and early tumor engraftment in vivo, alongside modest tumor suppression by LOS at later stages, suggesting that intrinsic AGTR1 activity may sustain tumor-promoting functions independently of AngII stimulation. These findings parallel the shorter recurrence-free survival in patients with high AGTR1 expression in our cohort.

We obtained additional intriguing insights into the role of AGTR1/AngII signaling cascade in cellular metabolism. In AGTR1 overexpressing bladder cancer cells, AngII stimulation led to the upregulation of both mitochondrial respiration and glycolytic activity. Notably, these responses were not suppressed by LOS treatment, despite being dependent on AGTR1, as confirmed by the lack of response in control cells exposed to AngII, as well as the negligible expression of alternative receptors, such as angiotensin II type 2 receptor or Mas (data not shown). Although the maximum OCR and ECAR were elevated by AGTR1/AngII signaling, the magnitude of this increase was relatively modest compared to non-cancerous cells. Specifically, OCR and ECAR increased by approximately 1.1- to 1.2-fold in T24 cells, whereas our previous study demonstrated nearly a five-fold increase in these metabolic parameters in T cells stimulated via the T cell receptor [46]. Given the inherently high metabolic demands of cancer cells, it is plausible that their energy production systems operate near maximal capacity, thereby limiting the impact of additional AngII stimulation. Thus, the enhanced invasion and migration activities observed upon AngII stimulation in our in vitro experiments are likely mediated primarily through ERK/NF‑κB/EMT signaling rather than through metabolism upregulation, consistent with the modest magnitude of OCR/ECAR changes. Nevertheless, even a small increase in mitochondrial and glycolytic activity, occurring in parallel with Akt suppression and ERK dominance, may provide ancillary support for tumor progression in vivo, particularly under conditions where bioenergetic systems operate near maximal capacity. Importantly, these metabolic changes were not reversed by LOS, possibly because competitive antagonism may not fully block AGTR1 under supraphysiological AngII concentrations, leaving residual receptor activity that could sustain bioenergetic adjustments through mechanisms, such as Ca^2+^-linked events, that are probably pharmacologically less sensitive and functionally buffered by the high basal metabolic tone of T24 cells.

Our study has two limitations: (1) AGTR1-overexpressing cells were engineered and may surpass patient expression levels, even among the ~60% high-expression cohort; (2) AngII concentrations exceeded physiological norms. To mitigate these, we performed time-course, dose–response, receptor-dependence, and in vivo validations. Thus, our model should be viewed as subtype-mimetic rather than universal. Future prospective studies stratified by AGTR1 expression are essential.

In summary, the expression of AGTR1 is associated with the prognosis of bladder cancer patients. The overexpression of AGTR1 enhances responsiveness to AngII, which is accompanied by a significant upregulation of ERK phosphorylation and a suppression of Akt phosphorylation. The upregulation of pathways mediated by EMT, NF-κB, and mTORC1 appears to contribute to tumor progression driven by AGTR1 overexpression under high concentrations of AngII. Both in vitro and in vivo experiments have demonstrated that LOS effectively inhibits the tumor’s latent potential. These findings suggest that LOS could serve as a promising therapeutic strategy for patients with tumors that exhibit high levels of AGTR1 expression. Future work should prioritize prospective trials stratified by AGTR1 expression to define the clinical utility of LOS-based therapy.

## Supplementary information

Supplementary information

Table S1

Table S2

Table S3

Table S4

Supplementary Figure 1

Supplementary Figure 2

Supplementary Figure 3

Supplementary Figure 4

Supplementary Figure 5

Supplementary Figure 6

Supplementary Figure 7

Supplementary legend
