# Supplementary material for: Angiotensin II type 1 receptor signaling promotes bladder cancer progression and its inhibition by Losartan
Source: Hypertens Res. 2026 Jan 19;49(4):1480–94. doi: 10.1038/s41440-025-02535-y (PMC13050642; doi:10.1038/s41440-025-02535-y)
Supplement: Supplementary file 1 — Supplementary information [file 41440_2025_2535_MOESM1_ESM.docx]

Table S1. Comparison of clinical characteristics between angiotensin II type 1 receptor-strong and -weak tumors from the patients who underwent transurethral resection of bladder tumor.

|  | Cases (% of each) | |  |
| --- | --- | --- | --- |
| AGTR1 | Strong | Weak |  |
|  | *n* = 34 | *n* = 21 | *p* value*^d^* |
| Age |  |  | 0.61 |
| < 75 | 22 (64.7) | 15 (71.4) |  |
| 75 ≦ | 12 (35.3) | 6 (28.6) |  |
| Sex |  |  | 0.3 |
| Male | 33 (97.1) | 19 (90.5) |  |
| Female | 1 (2.9) | 2 (9.5) |  |
| T stage*^a^* |  |  | 0.29 |
| T1 | 9 (26.5) | 3 (14.3) |  |
| Ta | 25 (73.5) | 18 (85.7) |  |
| Tumor grade*^b^* |  |  | 0.28 |
| G1/G2 | 23 (67.6) | 17 (81.0) |  |
| G3 | 11 (32.4) | 4 (19.0) |  |
| Histological variant*^c^* |  |  | 0.20 |
| No | 34 (100) | 20 (95.2) |  |
| Yes | 0 (0) | 1 (4.8) *^c^* | |
| Tumor number |  |  | 0.07 |
| Multiple | 20 (58.8) | 7 (33.3) |  |
| Single | 14 (41.2) | 14 (66.6) |  |
| Smoking | 29 (85.3) | 18 (85.7) | 0.97 |
| Hypertension | 18 (52.9) | 10 (47.6) | 0.70 |
| ARB*^e^* therapy | 11 (32.4) | 8 (38.1) | 0.67 |
| CCB*^f^* therapy | 13 (38.2) | 7 (33.3) | 0.71 |
| ARB and CCB combined | 6 (17.6) | 5 (23.8) | 0.58 |

*^a^*Classification defined by the Union for International Cancer Control

*^b^*Classification defined by the International Society of Urological Pathology

*^c^*Nested variant

*^d^*Evaluated by Pearson's chi-squared test.

*^e^*angiotensin II receptor blocker

*^f^*calcium channel blocker
