# Supplementary material for: Angiotensin II type 1 receptor signaling promotes bladder cancer progression and its inhibition by Losartan
Source: Hypertens Res. 2026 Jan 19;49(4):1480–94. doi: 10.1038/s41440-025-02535-y (PMC13050642; doi:10.1038/s41440-025-02535-y)
Supplement: Supplementary file 3 — Table S2 [file 41440_2025_2535_MOESM3_ESM.pdf]

Table S3: Top 20 commonly varied pathways identified across two comparisons: Angiotensin II (AngII) versus no stimulation, and AngII plus losartan versus AngII alone.

MSigDB H(hallmark) Term ID	MSigDB H(hallmark) Term Desc	MSigDB H(hallmark) Level1	MSigDB H(hallmark) Level2	Term Candidate Gene Num	Total Candidate Gene Num	Term Gene Num	Total Gene Num	Rich Ratio	P value	Q value	Gene ids	Gene Symbols
HALLMARK_TNFA_SIGNALING_VIA_NFKB	Genes regulated by NF-kB in response to TNF [GeneID=7124].	H	HALLMARK_TNFA_SIGNALING_VIA_NFKB	98	688	200	4378	0.49	8.41E-30	4.21E-28	10135,10209,10221,1026,10318,1051,10611,10769,10938,10957,11080,11182,1435,182,1827,1846,1942,1958,1960,2150,2152,22822,23258,23529,2355,23645,23764,24147,2643,2669,2683,2921,3164,3280,329,330,3371,3383,3491,3552,3553,3569,3604,3624,3726,374,388,3914,4609,4783,4790,4791,4792,4814,490,4929,4973,50486,5054,5055,51278,51561,5271,5328,5329,55332,5606,56937,5734,5743,597,604,6303,6364,6372,6385,6648,6776,687,6890,694,7050,7127,7185,7422,7538,8013,80176,8553,8613,8837,8848,8870,8877,9314,9334,960,969	NAMPT,EIF1,TRIB1,CDKN1A,TNIP1,CEBPB,PDLIM5,PLK2,EHD1,PNRC1,DNAJB4,SLC2A6,CSF1,JAG1,RCAN1,DUSP4,EFNA1,EGR1,EGR3,F2RL1,F3,PHLDA1,DENND5A,CLCF1,FOSL2,PPP1R15A,MAFF,FJX1,GCH1,GEM,B4GALT1,CXCL3,NR4A1,HES1,BIRC2,BIRC3,TNC,ICAM1,CCN1,IL1A,IL1B,IL6,TNFRSF9,INHBA,JUNB,AREG,RHOB,LAMB3,MYC,NFIL3,NFKB1,NFKB2,NFKBIA,NINJ1,ATP2B1,NR4A2,OLR1,G0S2,SERPINE1,SERPINB2,IER5,IL23A,SERPINB8,PLAU,PLAUR,DRAM1,MAP2K3,PMEPA1,PTGER4,PTGS2,BCL2A1,BCL6,SAT1,CCL20,CXCL6,SDC4,SOD2,STAT5A,KLF9,TAP1,BTG1,TGIF1,TNFAIP2,TRAF1,VEGFA,ZFP36,NR4A3,SPSB1,BHLHE40,PLPP3,CFLAR,TSC22D1,IER3,SPHK1,KLF4,B4GALT5,CD44,CD69
HALLMARK_UNFOLDED_PROTEIN_RESPONSE	Genes up-regulated during unfolded protein response, a cellular stress response related to the endoplasmic reticulum.	H	HALLMARK_UNFOLDED_PROTEIN_RESPONSE	47	688	113	4378	0.415929204	2.12E-11	5.30E-10	###	PREB,PDIA6,CEBPB,HYOU1,NOP56,CEBPG,MTHFD2,PDIA5,KDELR3,XPOT,ERN1,SEC31A,EXOSC2,FUS,SERP1,PSAT1,H2AX,HSPA5,HSPA9,DNAJB9,ASNS,ATF4,NFYA,NFYB,GEMIN4,DDIT4,FKBP14,WIPI1,ARFGAP1,DNAJC3,SRPRB,TSPYL2,SLC1A4,SRPRA,SSR1,TARS1,HSP90B1,VEGFA,WFS1,XBP1,CHAC1,CALR,SLC7A5,STC2,EIF2AK3,EDEM1,HERPUD1
HALLMARK_MTORC1_SIGNALING	Genes up-regulated through activation of mTORC1 complex.	H	HALLMARK_MTORC1_SIGNALING	66	688	200	4378	0.33	3.95E-10	6.59E-09	10135,1026,1075,10797,116496,134429,1491,1649,1719,2058,2181,23645,23657,23753,2542,26136,26227,27230,27346,2745,2805,2932,29968,3099,3308,3309,3313,3475,4171,4173,440,4783,4793,4860,488,51569,5236,54541,5606,5723,57761,5861,6117,6184,6472,6509,6510,6513,6652,6745,6781,7184,7277,7296,7374,7436,7453,7494,811,8140,8553,8615,8833,8894,9695,993	NAMPT,CDKN1A,CTSC,MTHFD2,NIBAN1,STARD4,CTH,DDIT3,DHFR,EPRS1,ACSL3,PPP1R15A,SLC7A11,SDF2L1,SLC37A4,TES,PHGDH,SERP1,TMEM97,GLRX,GOT1,GSK3B,PSAT1,HK2,HSPA4,HSPA5,HSPA9,IFRD1,MCM2,MCM4,ASNS,NFIL3,NFKBIB,PNP,ATP2A2,UFM1,PGM1,DDIT4,MAP2K3,PSPH,TRIB3,RAB1A,RPA1,RPN1,SHMT2,SLC1A4,SLC1A5,SLC2A1,SORD,SSR1,STC1,HSP90B1,TUBA4A,TXNRD1,UNG,VLDLR,WARS1,XBP1,CALR,SLC7A5,BHLHE40,USO1,GMPS,EIF2S2,EDEM1,CDC25A
HALLMARK_E2F_TARGETS	Genes encoding cell cycle related targets of E2F transcription factors.	H	HALLMARK_E2F_TARGETS	58	688	200	4378	0.29	8.16E-07	1.02E-05	1026,1027,10528,10535,10606,10714,10797,11168,1434,1786,203068,23047,23468,23649,27338,29028,3014,3070,3149,3835,3925,3930,3978,4001,4171,4172,4173,4174,4175,4176,4361,4609,5111,5411,54962,55148,55723,5591,57405,580,5982,6117,6118,6427,6470,64785,7112,7374,7884,79075,79077,7913,81611,9319,9837,9918,993,994	CDKN1A,CDKN1B,NOP56,RNASEH2A,PAICS,POLD3,MTHFD2,PSIP1,CSE1L,DNMT1,TUBB,PDS5B,CBX5,POLA2,UBE2S,ATAD2,H2AX,HELLS,HMGB3,KIF22,STMN1,LBR,LIG1,LMNB1,MCM2,MCM3,MCM4,MCM5,MCM6,MCM7,MRE11,MYC,PCNA,PNN,TIPIN,UBR7,ASF1B,PRKDC,SPC25,BARD1,RFC2,RPA1,RPA2,SRSF2,SHMT1,GINS3,TMPO,UNG,SLBP,DSCC1,DCTPP1,DEK,ANP32E,TRIP13,GINS1,NCAPD2,CDC25A,CDC25B
HALLMARK_HYPOXIA	Genes up-regulated in response to low oxygen levels (hypoxia).	H	HALLMARK_HYPOXIA	57	688	200	4378	0.285	1.90E-06	1.90E-05	1026,1027,10370,10397,10957,11015,133,1490,1649,1942,2131,2152,23210,23327,2355,23645,23764,2542,26136,2651,272,2745,2908,3069,3099,3162,3309,3340,3491,3569,3669,4502,4627,4783,5054,5155,5236,5329,54541,54800,6385,6478,6513,6576,6781,694,7422,7436,7538,839,8553,857,8614,8870,901,9469,9590	CDKN1A,CDKN1B,CITED2,NDRG1,PNRC1,KDELR3,ADM,CCN2,DDIT3,EFNA1,EXT1,F3,JMJD6,NEDD4L,FOSL2,PPP1R15A,MAFF,SLC37A4,TES,GCNT2,AMPD3,GLRX,NR3C1,HDLBP,HK2,HMOX1,HSPA5,NDST1,CCN1,IL6,ISG20,MT2A,MYH9,NFIL3,SERPINE1,PDGFB,PGM1,PLAUR,DDIT4,KLHL24,SDC4,SIAH2,SLC2A1,SLC25A1,STC1,BTG1,VEGFA,VLDLR,ZFP36,CASP6,BHLHE40,CAV1,STC2,IER3,CCNG2,CHST3,AKAP12
HALLMARK_EPITHELIAL_MESENCHYMAL_TRANSITION	Genes defining epithelial-mesenchymal transition, as in wound healing, fibrosis and metastasis.	H	HALLMARK_EPITHELIAL_MESENCHYMAL_TRANSITION	55	688	200	4378	0.275	9.48E-06	7.90E-05	10272,10398,11010,11167,1314,1490,1601,1809,2247,2335,2669,3371,3491,3569,3624,3673,374,388,3918,4176,4312,4314,4323,4837,4907,5054,51330,5217,5270,5329,5479,56937,57692,5817,5999,6303,6372,6382,6385,6695,6876,7049,7057,7171,7422,7424,7980,800,8038,813,8325,9235,9353,960,966	FSTL3,MYL9,GLIPR1,FSTL1,COPA,CCN2,DAB2,DPYSL3,FGF2,FN1,GEM,TNC,CCN1,IL6,INHBA,ITGA2,AREG,RHOB,LAMC2,MCM7,MMP1,MMP3,MMP14,NNMT,NT5E,SERPINE1,TNFRSF12A,PFN2,SERPINE2,PLAUR,PPIB,PMEPA1,MAGEE1,PVR,RGS4,SAT1,CXCL6,SDC1,SDC4,SPOCK1,TAGLN,TGFBR3,THBS1,TPM4,VEGFA,VEGFC,TFPI2,CALD1,ADAM12,CALU,FZD8,IL32,SLIT2,CD44,CD59
HALLMARK_UV_RESPONSE_DN	Genes down-regulated in response to ultraviolet (UV) radiation.	H	HALLMARK_UV_RESPONSE_DN	38	688	144	4378	0.263888889	5.72E-04	0.004087612	###	AKT3,CDKN1B,CITED2,PDLIM5,RBPMS,MGLL,NEK7,DAB2,LPAR1,F3,FHL2,ADGRL2,SYNE1,FYN,FZD2,SIPA1L1,NR3C1,APBB2,CCN1,IRS1,MYC,NFKB1,ATP2B1,SERPINE1,SFMBT1,RGS4,SLC7A1,TGFBR2,TGFBR3,TJP1,VLDLR,CDC42BPA,BHLHE40,CAV1,PLPP3,NRP1,SYNJ2,YTHDC1
HALLMARK_INFLAMMATORY_RESPONSE	Genes defining inflammatory response.	H	HALLMARK_INFLAMMATORY_RESPONSE	48	688	200	4378	0.24	0.001173469	0.007334181	###	NAMPT,CDKN1A,NLRP3,DCBLD2,SLC31A1,ADM,CSF1,CD55,SGMS2,LPAR1,AHR,EREG,F3,GCH1,LAMP3,HIF1A,ICAM1,IL1A,IL1B,IL6,TNFRSF9,INHBA,CD82,MMP14,MYC,NFKB1,NFKBIA,ATP2A2,ATP2B1,OLR1,P2RX4,P2RY2,SERPINE1,PLAUR,SELENOS,PTGER4,PVR,CCL20,CXCL6,SLC7A1,SLC7A2,C5AR1,IFITM1,SPHK1,OSMR,CD40,CD69,CD70
HALLMARK_G2M_CHECKPOINT	Genes involved in the G2/M checkpoint, as in progression through the cell division cycle.	H	HALLMARK_G2M_CHECKPOINT	47	688	199	4378	0.236180905	0.001882512	0.0104584	###	CHAF1A,CDKN1B,CENPA,SMC2,CBX1,E2F1,E2F2,PDS5B,POLA2,UBE2S,H2AX,HIF1A,HMGB3,HNRNPU,HSPA8,KIF22,KMT5A,STMN1,LBR,LMNB1,MCM2,MCM3,MCM5,MCM6,MEIS1,MT2A,MYC,NCL,ODC1,GINS2,PBK,BARD1,RBL1,RPA2,SRSF2,SLC7A1,SMARCC1,SNRPD1,TMPO,SLC7A5,SLC38A1,CDC7,RAD54L,YTHDC1,CDC6,CDC25A,CDC25B
HALLMARK_P53_PATHWAY	Genes involved in p53 pathways and networks.	H	HALLMARK_P53_PATHWAY	46	688	199	4378	0.231155779	0.003300316	0.01650158	###	TOB1,CDKN1A,NDRG1,SLC19A2,PLK2,PLK3,DDB2,DDIT3,EPHA2,F2R,PHLDA3,PPP1R15A,SLC7A11,SFN,SEC61A1,SERTAD3,HMOX1,IL1A,CD82,KRT17,TM4SF1,NINJ1,PCNA,IER5,PDGFA,SERPINB5,DDIT4,DRAM1,PIDD1,H2AJ,TRIB3,RAD9A,SAT1,SDC1,ABHD4,TSPYL2,AEN,SLC3A2,TAP1,BTG1,UPP1,CCNK,TSC22D1,IER3,SPHK1,KLF4
HALLMARK_ANDROGEN_RESPONSE	Genes defining response to androgens.	H	HALLMARK_ANDROGEN_RESPONSE	26	688	100	4378	0.26	0.005084587	0.02311176	10397,10611,11057,2181,220,22936,23223,2683,3005,4189,51465,5238,54407,56937,6303,6652,6675,6728,7520,87,8848,9053,9455,9510,9590,9871	NDRG1,PDLIM5,ABHD2,ACSL3,ALDH1A3,ELL2,RRP12,B4GALT1,H1-0,DNAJB9,UBE2J1,PGM3,SLC38A2,PMEPA1,SAT1,SORD,UAP1,SRP19,XRCC5,ACTN1,TSC22D1,MAP7,HOMER2,ADAMTS1,AKAP12,SEC24D
HALLMARK_CHOLESTEROL_HOMEOSTASIS	Genes involved in cholesterol homeostasis.	H	HALLMARK_CHOLESTEROL_HOMEOSTASIS	20	688	74	4378	0.27027027	0.008430719	0.035128	10957,116496,134429,182,214,2171,2222,27346,2770,4047,4597,4783,493869,51330,51478,5329,57761,6282,71,875	PNRC1,NIBAN1,STARD4,JAG1,ALCAM,FABP5,FDFT1,TMEM97,GNAI1,LSS,MVD,NFIL3,GPX8,TNFRSF12A,HSD17B7,PLAUR,TRIB3,S100A11,ACTG1,CBS
HALLMARK_ESTROGEN_RESPONSE_EARLY	Genes defining early response to estrogen.	H	HALLMARK_ESTROGEN_RESPONSE_EARLY	44	688	200	4378	0.22	0.0102294	0.03934385	###	TOB1,SLC19A2,ABHD2,PRSS23,EGR3,FDFT1,FHL2,INPP5F,FKBP4,FLNB,RRP12,WWC1,MYOF,B4GALT1,SFN,HES1,AREG,MAST4,LAD1,AFF1,MYC,P2RY2,SLC37A1,PODXL,RPS6KA2,SIAH2,SLC1A4,SLC2A1,SLC7A2,SLC16A1,SVIL,WFS1,XBP1,SLC7A5,BHLHE40,STC2,NAV2,PAPSS2,DHRS3,KLF4,PTGES,CD44,GAB2,MED24
HALLMARK_APOPTOSIS	Genes mediating programmed cell death (apoptosis) by activation of caspases.	H	HALLMARK_APOPTOSIS	36	688	161	4378	0.223602484	0.01495545	0.05341232	###	CDKN1A,CDKN1B,CTH,CYLD,DDIT3,EGR3,EREG,F2R,GCH1,PDCD4,H1-0,HMOX1,BIRC3,DNAJA1,IL1A,IL1B,IL6,ISG20,RHOB,TNFRSF12A,PLAT,PPP2R5B,DNAJC3,SAT1,SLC20A1,SOD2,TAP1,TGFBR3,CASP4,CASP6,CAV1,CFLAR,IER3,CD44,CD69,CDC25B
HALLMARK_ESTROGEN_RESPONSE_LATE	Genes defining late response to estrogen.	H	HALLMARK_ESTROGEN_RESPONSE_LATE	43	688	200	4378	0.215	0.01641336	0.0547112	10140,10535,11057,11098,12,1960,2030,2171,2222,224,2288,2317,26509,27250,2810,3669,3716,374,3918,4299,445,4665,51659,6196,64215,6478,6509,6566,6652,7466,7494,7517,7538,7980,8140,857,9060,9231,9314,9455,9536,960,990	TOB1,RNASEH2A,ABHD2,PRSS23,SERPINA3,EGR3,SLC29A1,FABP5,FDFT1,ALDH3A2,FKBP4,FLNB,MYOF,PDCD4,SFN,ISG20,JAK1,AREG,LAMC2,AFF1,ASS1,NAB2,GINS2,RPS6KA2,DNAJC1,SIAH2,SLC1A4,SLC16A1,SORD,WFS1,XBP1,XRCC3,ZFP36,TFPI2,SLC7A5,CAV1,PAPSS2,DLG5,KLF4,HOMER2,PTGES,CD44,CD
HALLMARK_WNT_BETA_CATENIN_SIGNALING	Genes up-regulated by activation of WNT signaling through accumulation of beta catenin CTNNB1 [GeneID=1499].	H	HALLMARK_WNT_BETA_CATENIN_SIGNALING	12	688	42	4378	0.285714286	0.02436238	0.07613244	###	HDAC5,CSNK1E,JAG1,GNAI1,MYC,PPARD,PTCH1,SKP2,ADAM17,AXIN1,FZD8,NUMB
HALLMARK_ANGIOGENESIS	Genes up-regulated during formation of blood vessels (angiogenesis).	H	HALLMARK_ANGIOGENESIS	10	688	36	4378	0.277777778	0.04575878	0.1345846	11,167,182,272,424,900,000,000,000,000,000,000,000,000	FSTL1,JAG1,TNFRSF21,OLR1,PDGFA,CXCL6,STC1,THBD,VEGFA,NRP1
HALLMARK_KRAS_SIGNALING_UP	Genes up-regulated by KRAS activation.	H	HALLMARK_KRAS_SIGNALING_UP	40	688	200	4378	0.2	0.05742326	0.1595091	101,10205,10221,10253,12,131566,2069,2119,2150,219333,220,22903,23136,23268,23531,23645,25907,2591,2745,2791,330,3553,3624,3673,3936,4319,4803,50486,5327,5328,5329,5743,6364,6447,6868,7185,8829,9053,9314,9590	ADAM8,MPZL2,TRIB1,SPRY2,SERPINA3,DCBLD2,EREG,ETV5,F2RL1,USP12,ALDH1A3,BTBD3,EPB41L3,DNMBP,MMD,PPP1R15A,TMEM158,GALNT3,GLRX,GNG11,BIRC3,IL1B,INHBA,ITGA2,LCP1,MMP10,NGF,G0S2,PLAT,PLAU,PLAUR,PTGS2,CCL20,SCG5,ADAM17,TRAF1,NRP1,MAP7,KLF4,AKAP12
HALLMARK_MYC_TARGETS_V2	A subgroup of genes regulated by MYC - version 2 (v2).	H	HALLMARK_MYC_TARGETS_V2	14	688	58	4378	0.24137931	0.06121343	0.161088	###	PRMT3,NOP56,CBX3,RRP12,TMEM97,HK2,MCM4,MCM5,MYC,MRTO4,SLC19A1,SORD,UNG,DCTPP1
HALLMARK_IL2_STAT5_SIGNALING	Genes up-regulated by STAT5 in response to IL2 stimulation.	H	HALLMARK_IL2_STAT5_SIGNALING	38	688	199	4378	0.190954774	0.1089819	0.2724548	###	NDRG1,CSF1,AHR,SPRED2,ALCAM,PHLDA1,DENND5A,NCS1,GABARAPL1,MAFF,TNFRSF21,HK2,TNFRSF9,IRF6,RHOB,MYC,NFIL3,PNP,NT5E,ODC1,P2RX4,SNX9,PRNP,PTCH1,NFKBIZ,CDCP1,SLC1A5,TRAF1,UMPS,XBP1,BHLHE40,ADAM19,NRP1,SOCS2,DHRS3,MAPKAPK2,CD44,CDC6
