# Supplementary material for: Angiotensin II type 1 receptor signaling promotes bladder cancer progression and its inhibition by Losartan
Source: Hypertens Res. 2026 Jan 19;49(4):1480–94. doi: 10.1038/s41440-025-02535-y (PMC13050642; doi:10.1038/s41440-025-02535-y)
Supplement: Supplementary file 4 — Table S3 [file 41440_2025_2535_MOESM4_ESM.pdf]

Table S4: Differentially expressed genes between AGTR1-overexpressing and control T24 bladder cancer cells.

Gene ID	Gene Symbol	Type	MSigDB H(hallmark) Term	MSigDB H(hallmark) Level1	MSigDB H(hallmark) Level2
100101467	'ZSCAN30'	mRNA			
100188893	'TOMM6'	mRNA			
1003	'CDH5'	mRNA			
100507421	'TMEM178B'	mRNA			
10076	'PTPRU'	mRNA			
10079	'ATP9A'	mRNA			
100820829	'MYZAP'	mRNA			
101	'ADAM8'	mRNA	HALLMARK_KRAS_SIGNALING_UP	H	HALLMARK_KRAS_SIGNALING_UP
10103	'TSPAN1'	mRNA	HALLMARK_KRAS_SIGNALING_UP	H	HALLMARK_KRAS_SIGNALING_UP
10131	'TRAP1'	mRNA			
10133	'OPTN'	mRNA	HALLMARK_BILE_ACID_METABOLISM+++HALLMARK_HEME_METABOLISM	H	HALLMARK_BILE_ACID_METABOLISM+++HALLMARK_HEME_METABOLISM
10135	'NAMPT'	mRNA	HALLMARK_INFLAMMATORY_RESPONSE+++HALLMARK_INTERFERON_GAMMA_RESPONSE+++HALLMARK_MTORC1_SIGNALING+++HALLMARK_TNFA_SIGNALING_VIA_NFKB	H	HALLMARK_INFLAMMATORY_RESPONSE+++HALLMARK_INTERFERON_GAMMA_RESPONSE+++HALLMARK_MTORC1_SIGNALING+++HALLMARK_TNFA_SIGNALING_VIA_NFKB
10140	'TOB1'	mRNA	HALLMARK_ADIPOGENESIS+++HALLMARK_ESTROGEN_RESPONSE_EARLY+++HALLMARK_ESTROGEN_RESPONSE_LATE+++HALLMARK_P53_PATHWAY	H	HALLMARK_ADIPOGENESIS+++HALLMARK_ESTROGEN_RESPONSE_EARLY+++HALLMARK_ESTROGEN_RESPONSE_LATE+++HALLMARK_P53_PATHWAY
10155	'TRIM28'	mRNA	HALLMARK_MYC_TARGETS_V1	H	HALLMARK_MYC_TARGETS_V1
10189	'ALYREF'	mRNA	HALLMARK_DNA_REPAIR	H	HALLMARK_DNA_REPAIR
1019	'CDK4'	mRNA	HALLMARK_E2F_TARGETS+++HALLMARK_G2M_CHECKPOINT+++HALLMARK_MYC_TARGETS_V1+++HALLMARK_MYC_TARGETS_V2+++HALLMARK_PI3K_AKT_MTOR_SIGNALING	H	HALLMARK_E2F_TARGETS+++HALLMARK_G2M_CHECKPOINT+++HALLMARK_MYC_TARGETS_V1+++HALLMARK_MYC_TARGETS_V2+++HALLMARK_PI3K_AKT_MTOR_SIGNALING
10204	'NUTF2'	mRNA			
10205	'MPZL2'	mRNA	HALLMARK_APICAL_JUNCTION+++HALLMARK_KRAS_SIGNALING_UP	H	HALLMARK_APICAL_JUNCTION+++HALLMARK_KRAS_SIGNALING_UP
10244	'RABEPK'	mRNA	HALLMARK_MYC_TARGETS_V2	H	HALLMARK_MYC_TARGETS_V2
10253	'SPRY2'	mRNA	HALLMARK_KRAS_SIGNALING_UP	H	HALLMARK_KRAS_SIGNALING_UP
1026	'CDKN1A'	mRNA	HALLMARK_APOPTOSIS+++HALLMARK_E2F_TARGETS+++HALLMARK_HYPOXIA+++HALLMARK_INFLAMMATORY_RESPONSE+++HALLMARK_INTERFERON_GAMMA_RESPONSE+++HALLMARK_MTORC1_SIGNALING+++HALLMARK_MYOGENESIS+++HALLMARK_P53_PATHWAY+++HALLMARK_PI3K_AKT_MTOR_SIGNALING+++HALLMARK_TNFA_SIGNALING_VIA_NFKB	H	HALLMARK_APOPTOSIS+++HALLMARK_E2F_TARGETS+++HALLMARK_HYPOXIA+++HALLMARK_INFLAMMATORY_RESPONSE+++HALLMARK_INTERFERON_GAMMA_RESPONSE+++HALLMARK_MTORC1_SIGNALING+++HALLMARK_MYOGENESIS+++HALLMARK_P53_PATHWAY+++HALLMARK_PI3K_AKT_MTOR_SIGNALING+++HALLMARK_TNFA_SIGNALING_VIA_NFKB
102723996	'LOC102723996'	mRNA			
10318	'TNIP1'	mRNA	HALLMARK_TNFA_SIGNALING_VIA_NFKB	H	HALLMARK_TNFA_SIGNALING_VIA_NFKB
10326	'SIRPB1'	mRNA			
10346	'TRIM22'	mRNA			
10370	'CITED2'	mRNA	HALLMARK_GLYCOLYSIS+++HALLMARK_HYPOXIA+++HALLMARK_UV_RESPONSE_DN	H	HALLMARK_GLYCOLYSIS+++HALLMARK_HYPOXIA+++HALLMARK_UV_RESPONSE_DN
10371	'SEMA3A'	mRNA			
10376	'TUBA1B'	mRNA			
10383	'TUBB4B'	mRNA			
10395	'DLC1'	mRNA	HALLMARK_ESTROGEN_RESPONSE_EARLY+++HALLMARK_UV_RESPONSE_DN	H	HALLMARK_ESTROGEN_RESPONSE_EARLY+++HALLMARK_UV_RESPONSE_DN
10397	'NDRG1'	mRNA	HALLMARK_ANDROGEN_RESPONSE+++HALLMARK_HYPOXIA+++HALLMARK_IL2_STAT5_SIGNALING+++HALLMARK_P53_PATHWAY	H	HALLMARK_ANDROGEN_RESPONSE+++HALLMARK_HYPOXIA+++HALLMARK_IL2_STAT5_SIGNALING+++HALLMARK_P53_PATHWAY
10398	'MYL9'	mRNA	HALLMARK_APICAL_JUNCTION+++HALLMARK_EPITHELIAL_MESENCHYMAL_TRANSITION	H	HALLMARK_APICAL_JUNCTION+++HALLMARK_EPITHELIAL_MESENCHYMAL_TRANSITION
10413	'YAP1'	mRNA			
10452	'TOMM40'	mRNA	HALLMARK_MTORC1_SIGNALING	H	HALLMARK_MTORC1_SIGNALING
10468	'FST'	mRNA	HALLMARK_MYOGENESIS	H	HALLMARK_MYOGENESIS
1047	'CLGN'	mRNA	HALLMARK_SPERMATOGENESIS	H	HALLMARK_SPERMATOGENESIS
10484	'SEC23A'	mRNA			
10509	'SEMA4B'	mRNA			
1051	'CEBPB'	mRNA	HALLMARK_COMPLEMENT+++HALLMARK_TNFA_SIGNALING_VIA_NFKB+++HALLMARK_UNFOLDED_PROTEIN_RESPONSE	H	HALLMARK_COMPLEMENT+++HALLMARK_TNFA_SIGNALING_VIA_NFKB+++HALLMARK_UNFOLDED_PROTEIN_RESPONSE
10514	'MYBBP1A'	mRNA	HALLMARK_ESTROGEN_RESPONSE_EARLY+++HALLMARK_MYC_TARGETS_V2	H	HALLMARK_ESTROGEN_RESPONSE_EARLY+++HALLMARK_MYC_TARGETS_V2
10528	'NOP56'	mRNA	HALLMARK_E2F_TARGETS+++HALLMARK_MYC_TARGETS_V1+++HALLMARK_MYC_TARGETS_V2+++HALLMARK_UNFOLDED_PROTEIN_RESPONSE	H	HALLMARK_E2F_TARGETS+++HALLMARK_MYC_TARGETS_V1+++HALLMARK_MYC_TARGETS_V2+++HALLMARK_UNFOLDED_PROTEIN_RESPONSE
10576	'CCT2'	mRNA	HALLMARK_MYC_TARGETS_V1	H	HALLMARK_MYC_TARGETS_V1
1059	'CENPB'	mRNA			
10606	'PAICS'	mRNA	HALLMARK_E2F_TARGETS	H	HALLMARK_E2F_TARGETS
10625	'IVNS1ABP'	mRNA			
10630	'PDPN'	mRNA	HALLMARK_INFLAMMATORY_RESPONSE	H	HALLMARK_INFLAMMATORY_RESPONSE
10643	'IGF2BP3'	mRNA			
10686	'CLDN16'	mRNA	HALLMARK_KRAS_SIGNALING_DN	H	HALLMARK_KRAS_SIGNALING_DN
10694	'CCT8'	mRNA			
10723	'SLC12A7'	mRNA			
1075	'CTSC'	mRNA	HALLMARK_COMPLEMENT+++HALLMARK_MTORC1_SIGNALING+++HALLMARK_PROTEIN_SECRETION	H	HALLMARK_COMPLEMENT+++HALLMARK_MTORC1_SIGNALING+++HALLMARK_PROTEIN_SECRETION
10808	'HSPH1'	mRNA	HALLMARK_FATTY_ACID_METABOLISM	H	HALLMARK_FATTY_ACID_METABOLISM
10849	'POLR1G'	mRNA			
10879	'SMR3B'	mRNA			
10910	'SUGT1'	mRNA			
10935	'PRDX3'	mRNA	HALLMARK_ADIPOGENESIS+++HALLMARK_MYC_TARGETS_V1+++HALLMARK_OXIDATIVE_PHOSPHORYLATION	H	HALLMARK_ADIPOGENESIS+++HALLMARK_MYC_TARGETS_V1+++HALLMARK_OXIDATIVE_PHOSPHORYLATION
109703458	'HTD2'	mRNA			
10971	'YWHAQ'	mRNA	HALLMARK_MYC_TARGETS_V1	H	HALLMARK_MYC_TARGETS_V1
10980	'COPS6'	mRNA			
11007	'CCDC85B'	mRNA			
11015	'KDELR3'	mRNA	HALLMARK_GLYCOLYSIS+++HALLMARK_HYPOXIA+++HALLMARK_UNFOLDED_PROTEIN_RESPONSE	H	HALLMARK_GLYCOLYSIS+++HALLMARK_HYPOXIA+++HALLMARK_UNFOLDED_PROTEIN_RESPONSE
1104	'RCC1'	mRNA			
1106	'CHD2'	mRNA			
11065	'UBE2C'	mRNA	HALLMARK_G2M_CHECKPOINT	H	HALLMARK_G2M_CHECKPOINT
11067	'DEPP1'	mRNA			
11098	'PRSS23'	mRNA	HALLMARK_COAGULATION+++HALLMARK_ESTROGEN_RESPONSE_EARLY+++HALLMARK_ESTROGEN_RESPONSE_LATE	H	HALLMARK_COAGULATION+++HALLMARK_ESTROGEN_RESPONSE_EARLY+++HALLMARK_ESTROGEN_RESPONSE_LATE
11102	'RPP14'	mRNA			
11118	'BTN3A2'	mRNA			
1112	'FOXN3'	mRNA	HALLMARK_G2M_CHECKPOINT	H	HALLMARK_G2M_CHECKPOINT
11190	'CEP250'	mRNA	HALLMARK_MITOTIC_SPINDLE	H	HALLMARK_MITOTIC_SPINDLE
11222	'MRPL3'	mRNA	HALLMARK_ALLOGRAFT_REJECTION	H	HALLMARK_ALLOGRAFT_REJECTION
112399	'EGLN3'	mRNA	HALLMARK_GLYCOLYSIS+++HALLMARK_MTORC1_SIGNALING	H	HALLMARK_GLYCOLYSIS+++HALLMARK_MTORC1_SIGNALING
11245	'GPR176'	mRNA			
112495	'GTF3C6'	mRNA			
11282	'MGAT4B'	mRNA			
113000	'RPUSD1'	mRNA			
113146	'AHNAK2'	mRNA			
11325	'DDX42'	mRNA			
11332	'ACOT7'	mRNA			
11343	'MGLL'	mRNA	HALLMARK_ADIPOGENESIS+++HALLMARK_FATTY_ACID_METABOLISM+++HALLMARK_UV_RESPONSE_DN	H	HALLMARK_ADIPOGENESIS+++HALLMARK_FATTY_ACID_METABOLISM+++HALLMARK_UV_RESPONSE_DN
113829	'SLC35A4'	mRNA			
114897	'C1QTNF1'	mRNA			
115098	'CCDC124'	mRNA			
115811	'IQCD'	mRNA			
1163	'CKS1B'	mRNA	HALLMARK_E2F_TARGETS+++HALLMARK_G2M_CHECKPOINT+++HALLMARK_UNFOLDED_PROTEIN_RESPONSE	H	HALLMARK_E2F_TARGETS+++HALLMARK_G2M_CHECKPOINT+++HALLMARK_UNFOLDED_PROTEIN_RESPONSE
116372	'LYPD1'	mRNA			
116496	'NIBAN1'	mRNA	HALLMARK_CHOLESTEROL_HOMEOSTASIS+++HALLMARK_MTORC1_SIGNALING	H	HALLMARK_CHOLESTEROL_HOMEOSTASIS+++HALLMARK_MTORC1_SIGNALING
118460	'EXOSC6'	mRNA			
118980	'SFXN2'	mRNA			
124905557	'LOC124905557'	mRNA			
124935	'SLC43A2'	mRNA			
126789	'PUSL1'	mRNA			
126792	'B3GALT6'	mRNA	HALLMARK_GLYCOLYSIS+++HALLMARK_HYPOXIA	H	HALLMARK_GLYCOLYSIS+++HALLMARK_HYPOXIA
127687	'C1orf122'	mRNA			
1282	'COL4A1'	mRNA	HALLMARK_ADIPOGENESIS+++HALLMARK_EPITHELIAL_MESENCHYMAL_TRANSITION	H	HALLMARK_ADIPOGENESIS+++HALLMARK_EPITHELIAL_MESENCHYMAL_TRANSITION
1284	'COL4A2'	mRNA	HALLMARK_COMPLEMENT+++HALLMARK_EPITHELIAL_MESENCHYMAL_TRANSITION+++HALLMARK_MYOGENESIS	H	HALLMARK_COMPLEMENT+++HALLMARK_EPITHELIAL_MESENCHYMAL_TRANSITION+++HALLMARK_MYOGENESIS
1288	'COL4A6'	mRNA			
1295	'COL8A1'	mRNA			
1303	'COL12A1'	mRNA	HALLMARK_EPITHELIAL_MESENCHYMAL_TRANSITION	H	HALLMARK_EPITHELIAL_MESENCHYMAL_TRANSITION
130367	'SGPP2'	mRNA			
1308	'COL17A1'	mRNA	HALLMARK_APICAL_JUNCTION	H	HALLMARK_APICAL_JUNCTION
130951	'M1AP'	mRNA			
131368	'ZPLD1'	mRNA			
131873	'COL6A6'	mRNA			
1347	'COX7A2'	mRNA	HALLMARK_OXIDATIVE_PHOSPHORYLATION	H	HALLMARK_OXIDATIVE_PHOSPHORYLATION
1349	'COX7B'	mRNA	HALLMARK_ADIPOGENESIS+++HALLMARK_OXIDATIVE_PHOSPHORYLATION	H	HALLMARK_ADIPOGENESIS+++HALLMARK_OXIDATIVE_PHOSPHORYLATION
1350	'COX7C'	mRNA	HALLMARK_OXIDATIVE_PHOSPHORYLATION	H	HALLMARK_OXIDATIVE_PHOSPHORYLATION
1351	'COX8A'	mRNA	HALLMARK_ADIPOGENESIS+++HALLMARK_OXIDATIVE_PHOSPHORYLATION	H	HALLMARK_ADIPOGENESIS+++HALLMARK_OXIDATIVE_PHOSPHORYLATION
135228	'CD109'	mRNA			
1374	'CPT1A'	mRNA	HALLMARK_FATTY_ACID_METABOLISM+++HALLMARK_OXIDATIVE_PHOSPHORYLATION	H	HALLMARK_FATTY_ACID_METABOLISM+++HALLMARK_OXIDATIVE_PHOSPHORYLATION
140576	'S100A16'	mRNA			
1434	'CSE1L'	mRNA	HALLMARK_E2F_TARGETS	H	HALLMARK_E2F_TARGETS
1435	'CSF1'	mRNA	HALLMARK_ALLOGRAFT_REJECTION+++HALLMARK_IL2_STAT5_SIGNALING+++HALLMARK_IL6_JAK_STAT3_SIGNALING+++HALLMARK_INFLAMMATORY_RESPONSE+++HALLMARK_INTERFERON_ALPHA_RESPONSE+++HALLMARK_TNFA_SIGNALING_VIA_NFKB	H	HALLMARK_ALLOGRAFT_REJECTION+++HALLMARK_IL2_STAT5_SIGNALING+++HALLMARK_IL6_JAK_STAT3_SIGNALING+++HALLMARK_INFLAMMATORY_RESPONSE+++HALLMARK_INTERFERON_ALPHA_RESPONSE+++HALLMARK_TNFA_SIGNALING_VIA_NFKB
1437	'CSF2'	mRNA	HALLMARK_IL2_STAT5_SIGNALING+++HALLMARK_IL6_JAK_STAT3_SIGNALING+++HALLMARK_KRAS_SIGNALING_UP+++HALLMARK_TNFA_SIGNALING_VIA_NFKB	H	HALLMARK_IL2_STAT5_SIGNALING+++HALLMARK_IL6_JAK_STAT3_SIGNALING+++HALLMARK_KRAS_SIGNALING_UP+++HALLMARK_TNFA_SIGNALING_VIA_NFKB
143903	'LAYN'	mRNA	HALLMARK_APICAL_JUNCTION	H	HALLMARK_APICAL_JUNCTION
145282	'MIPOL1'	mRNA			
145447	'ABHD12B'	mRNA			
1460	'CSNK2B'	mRNA	HALLMARK_PI3K_AKT_MTOR_SIGNALING	H	HALLMARK_PI3K_AKT_MTOR_SIGNALING
1468	'SLC25A10'	mRNA	HALLMARK_ADIPOGENESIS+++HALLMARK_GLYCOLYSIS	H	HALLMARK_ADIPOGENESIS+++HALLMARK_GLYCOLYSIS
1469	'CST1'	mRNA			
147381	'CBLN2'	mRNA			
1490	'CCN2'	mRNA	HALLMARK_EPITHELIAL_MESENCHYMAL_TRANSITION+++HALLMARK_HYPOXIA	H	HALLMARK_EPITHELIAL_MESENCHYMAL_TRANSITION+++HALLMARK_HYPOXIA
149111	'CNIH3'	mRNA			
149175	'MANEAL'	mRNA			
1508	'CTSB'	mRNA	HALLMARK_COAGULATION+++HALLMARK_COMPLEMENT+++HALLMARK_HEME_METABOLISM	H	HALLMARK_COAGULATION+++HALLMARK_COMPLEMENT+++HALLMARK_HEME_METABOLISM
1514	'CTSL'	mRNA	HALLMARK_COMPLEMENT	H	HALLMARK_COMPLEMENT
152002	'XXYLT1'	mRNA			
153579	'BTNL9'	mRNA			
157	'GRK3'	mRNA			
158	'ADSL'	mRNA	HALLMARK_FATTY_ACID_METABOLISM	H	HALLMARK_FATTY_ACID_METABOLISM
160760	'PPTC7'	mRNA			
1622	'DBI'	mRNA	HALLMARK_ANDROGEN_RESPONSE	H	HALLMARK_ANDROGEN_RESPONSE
162394	'SLFN5'	mRNA			
164312	'LRRN4'	mRNA			
1662	'DDX10'	mRNA	HALLMARK_UNFOLDED_PROTEIN_RESPONSE	H	HALLMARK_UNFOLDED_PROTEIN_RESPONSE
1736	'DKC1'	mRNA	HALLMARK_G2M_CHECKPOINT+++HALLMARK_UNFOLDED_PROTEIN_RESPONSE	H	HALLMARK_G2M_CHECKPOINT+++HALLMARK_UNFOLDED_PROTEIN_RESPONSE
1741	'DLG3'	mRNA			
1742	'DLG4'	mRNA	HALLMARK_PEROXISOME+++HALLMARK_UV_RESPONSE_UP	H	HALLMARK_PEROXISOME+++HALLMARK_UV_RESPONSE_UP
1788	'DNMT3A'	mRNA			
182	'JAG1'	mRNA	HALLMARK_ANGIOGENESIS+++HALLMARK_CHOLESTEROL_HOMEOSTASIS+++HALLMARK_NOTCH_SIGNALING+++HALLMARK_TNFA_SIGNALING_VIA_NFKB+++HALLMARK_WNT_BETA_CATENIN_SIGNALING	H	HALLMARK_ANGIOGENESIS+++HALLMARK_CHOLESTEROL_HOMEOSTASIS+++HALLMARK_NOTCH_SIGNALING+++HALLMARK_TNFA_SIGNALING_VIA_NFKB+++HALLMARK_WNT_BETA_CATENIN_SIGNALING
1830	'DSG3'	mRNA			
1839	'HBEGF'	mRNA	HALLMARK_INFLAMMATORY_RESPONSE+++HALLMARK_KRAS_SIGNALING_UP+++HALLMARK_MYOGENESIS+++HALLMARK_P53_PATHWAY+++HALLMARK_TNFA_SIGNALING_VIA_NFKB	H	HALLMARK_INFLAMMATORY_RESPONSE+++HALLMARK_KRAS_SIGNALING_UP+++HALLMARK_MYOGENESIS+++HALLMARK_P53_PATHWAY+++HALLMARK_TNFA_SIGNALING_VIA_NFKB
1846	'DUSP4'	mRNA	HALLMARK_TNFA_SIGNALING_VIA_NFKB	H	HALLMARK_TNFA_SIGNALING_VIA_NFKB
185	'AGTR1'	mRNA			
19	'ABCA1'	mRNA	HALLMARK_ADIPOGENESIS+++HALLMARK_BILE_ACID_METABOLISM+++HALLMARK_INFLAMMATORY_RESPONSE+++HALLMARK_PROTEIN_SECRETION+++HALLMARK_TNFA_SIGNALING_VIA_NFKB	H	HALLMARK_ADIPOGENESIS+++HALLMARK_BILE_ACID_METABOLISM+++HALLMARK_INFLAMMATORY_RESPONSE+++HALLMARK_PROTEIN_SECRETION+++HALLMARK_TNFA_SIGNALING_VIA_NFKB
1901	'S1PR1'	mRNA			
1902	'LPAR1'	mRNA	HALLMARK_INFLAMMATORY_RESPONSE+++HALLMARK_UV_RESPONSE_DN	H	HALLMARK_INFLAMMATORY_RESPONSE+++HALLMARK_UV_RESPONSE_DN
1903	'S1PR3'	mRNA			
1917	'EEF1A2'	mRNA			
192111	'PGAM5'	mRNA			
192683	'SCAMP5'	mRNA			
1973	'EIF4A1'	mRNA	HALLMARK_MYC_TARGETS_V1+++HALLMARK_UNFOLDED_PROTEIN_RESPONSE	H	HALLMARK_MYC_TARGETS_V1+++HALLMARK_UNFOLDED_PROTEIN_RESPONSE
1978	'EIF4EBP1'	mRNA	HALLMARK_UNFOLDED_PROTEIN_RESPONSE	H	HALLMARK_UNFOLDED_PROTEIN_RESPONSE
1999	'ELF3'	mRNA	HALLMARK_ESTROGEN_RESPONSE_EARLY+++HALLMARK_GLYCOLYSIS	H	HALLMARK_ESTROGEN_RESPONSE_EARLY+++HALLMARK_GLYCOLYSIS
20	'ABCA2'	mRNA	HALLMARK_BILE_ACID_METABOLISM+++HALLMARK_CHOLESTEROL_HOMEOSTASIS	H	HALLMARK_BILE_ACID_METABOLISM+++HALLMARK_CHOLESTEROL_HOMEOSTASIS
200734	'SPRED2'	mRNA	HALLMARK_IL2_STAT5_SIGNALING	H	HALLMARK_IL2_STAT5_SIGNALING
2010	'EMD'	mRNA			
201191	'SAMD14'	mRNA			
2017	'CTTN'	mRNA	HALLMARK_MITOTIC_SPINDLE	H	HALLMARK_MITOTIC_SPINDLE
201895	'SMIM14'	mRNA			
2026	'ENO2'	mRNA	HALLMARK_APOPTOSIS+++HALLMARK_EPITHELIAL_MESENCHYMAL_TRANSITION+++HALLMARK_FATTY_ACID_METABOLISM+++HALLMARK_GLYCOLYSIS+++HALLMARK_HYPOXIA+++HALLMARK_UV_RESPONSE_UP	H	HALLMARK_APOPTOSIS+++HALLMARK_EPITHELIAL_MESENCHYMAL_TRANSITION+++HALLMARK_FATTY_ACID_METABOLISM+++HALLMARK_GLYCOLYSIS+++HALLMARK_HYPOXIA+++HALLMARK_UV_RESPONSE_UP
203068	'TUBB'	mRNA	HALLMARK_E2F_TARGETS	H	HALLMARK_E2F_TARGETS
2034	'EPAS1'	mRNA			
2035	'EPB41'	mRNA	HALLMARK_HEME_METABOLISM+++HALLMARK_MITOTIC_SPINDLE	H	HALLMARK_HEME_METABOLISM+++HALLMARK_MITOTIC_SPINDLE
2036	'EPB41L1'	mRNA			
204	'AK2'	mRNA	HALLMARK_ADIPOGENESIS+++HALLMARK_E2F_TARGETS	H	HALLMARK_ADIPOGENESIS+++HALLMARK_E2F_TARGETS
2047	'EPHB1'	mRNA			
205	'AK4'	mRNA	HALLMARK_GLYCOLYSIS+++HALLMARK_HYPOXIA+++HALLMARK_MTORC1_SIGNALING	H	HALLMARK_GLYCOLYSIS+++HALLMARK_HYPOXIA+++HALLMARK_MTORC1_SIGNALING
205860	'TRIML2'	mRNA			
2091	'FBL'	mRNA	HALLMARK_MYC_TARGETS_V1	H	HALLMARK_MYC_TARGETS_V1
2150	'F2RL1'	mRNA	HALLMARK_KRAS_SIGNALING_UP+++HALLMARK_TNFA_SIGNALING_VIA_NFKB	H	HALLMARK_KRAS_SIGNALING_UP+++HALLMARK_TNFA_SIGNALING_VIA_NFKB
2167	'FABP4'	mRNA	HALLMARK_ADIPOGENESIS	H	HALLMARK_ADIPOGENESIS
2171	'FABP5'	mRNA	HALLMARK_CHOLESTEROL_HOMEOSTASIS+++HALLMARK_ESTROGEN_RESPONSE_LATE	H	HALLMARK_CHOLESTEROL_HOMEOSTASIS+++HALLMARK_ESTROGEN_RESPONSE_LATE
2194	'FASN'	mRNA	HALLMARK_CHOLESTEROL_HOMEOSTASIS+++HALLMARK_ESTROGEN_RESPONSE_EARLY+++HALLMARK_FATTY_ACID_METABOLISM	H	HALLMARK_CHOLESTEROL_HOMEOSTASIS+++HALLMARK_ESTROGEN_RESPONSE_EARLY+++HALLMARK_FATTY_ACID_METABOLISM
2202	'EFEMP1'	mRNA	HALLMARK_UV_RESPONSE_DN	H	HALLMARK_UV_RESPONSE_DN
220323	'OAF'	mRNA			
2250	'FGF5'	mRNA			
22846	'VASH1'	mRNA			
22859	'ADGRL1'	mRNA			
22866	'CNKSR2'	mRNA			
22874	'PLEKHA6'	mRNA			
2288	'FKBP4'	mRNA	HALLMARK_ESTROGEN_RESPONSE_EARLY+++HALLMARK_ESTROGEN_RESPONSE_LATE+++HALLMARK_GLYCOLYSIS+++HALLMARK_UV_RESPONSE_UP	H	HALLMARK_ESTROGEN_RESPONSE_EARLY+++HALLMARK_ESTROGEN_RESPONSE_LATE+++HALLMARK_GLYCOLYSIS+++HALLMARK_UV_RESPONSE_UP
22905	'EPN2'	mRNA			
22948	'CCT5'	mRNA	HALLMARK_MYC_TARGETS_V1	H	HALLMARK_MYC_TARGETS_V1
22999	'RIMS1'	mRNA			
23022	'PALLD'	mRNA	HALLMARK_MITOTIC_SPINDLE	H	HALLMARK_MITOTIC_SPINDLE
2303	'FOXC2'	mRNA	HALLMARK_EPITHELIAL_MESENCHYMAL_TRANSITION	H	HALLMARK_EPITHELIAL_MESENCHYMAL_TRANSITION
23051	'ZHX3'	mRNA			
23052	'ENDOD1'	mRNA	HALLMARK_ESTROGEN_RESPONSE_EARLY+++HALLMARK_HEME_METABOLISM	H	HALLMARK_ESTROGEN_RESPONSE_EARLY+++HALLMARK_HEME_METABOLISM
23075	'SWAP70'	mRNA	HALLMARK_IL2_STAT5_SIGNALING	H	HALLMARK_IL2_STAT5_SIGNALING
231	'AKR1B1'	mRNA			
2317	'FLNB'	mRNA	HALLMARK_ESTROGEN_RESPONSE_EARLY+++HALLMARK_ESTROGEN_RESPONSE_LATE+++HALLMARK_MITOTIC_SPINDLE	H	HALLMARK_ESTROGEN_RESPONSE_EARLY+++HALLMARK_ESTROGEN_RESPONSE_LATE+++HALLMARK_MITOTIC_SPINDLE
23170	'TTLL12'	mRNA			
23199	'GSE1'	mRNA			
23212	'RRS1'	mRNA			
23220	'DTX4'	mRNA	HALLMARK_NOTCH_SIGNALING	H	HALLMARK_NOTCH_SIGNALING
23231	'SEL1L3'	mRNA			
23246	'BOP1'	mRNA			
23258	'DENND5A'	mRNA	HALLMARK_IL2_STAT5_SIGNALING+++HALLMARK_TNFA_SIGNALING_VIA_NFKB	H	HALLMARK_IL2_STAT5_SIGNALING+++HALLMARK_TNFA_SIGNALING_VIA_NFKB
23277	'CLUH'	mRNA			
23302	'WSCD1'	mRNA			
23327	'NEDD4L'	mRNA	HALLMARK_HYPOXIA	H	HALLMARK_HYPOXIA
23363	'OBSL1'	mRNA			
23365	'ARHGEF12'	mRNA	HALLMARK_HEME_METABOLISM+++HALLMARK_MITOTIC_SPINDLE	H	HALLMARK_HEME_METABOLISM+++HALLMARK_MITOTIC_SPINDLE
23433	'RHOQ'	mRNA			
23464	'GCAT'	mRNA			
23467	'NPTXR'	mRNA	HALLMARK_UV_RESPONSE_UP	H	HALLMARK_UV_RESPONSE_UP
23475	'QPRT'	mRNA			
23657	'SLC7A11'	mRNA	HALLMARK_HEME_METABOLISM+++HALLMARK_MTORC1_SIGNALING+++HALLMARK_P53_PATHWAY	H	HALLMARK_HEME_METABOLISM+++HALLMARK_MTORC1_SIGNALING+++HALLMARK_P53_PATHWAY
23710	'GABARAPL1'	mRNA	HALLMARK_FATTY_ACID_METABOLISM+++HALLMARK_IL2_STAT5_SIGNALING+++HALLMARK_XENOBIOTIC_METABOLISM	H	HALLMARK_FATTY_ACID_METABOLISM+++HALLMARK_IL2_STAT5_SIGNALING+++HALLMARK_XENOBIOTIC_METABOLISM
23780	'APOL2'	mRNA			
241	'ALOX5AP'	mRNA			
24147	'FJX1'	mRNA	HALLMARK_TNFA_SIGNALING_VIA_NFKB	H	HALLMARK_TNFA_SIGNALING_VIA_NFKB
2534	'FYN'	mRNA	HALLMARK_COAGULATION+++HALLMARK_COMPLEMENT+++HALLMARK_UV_RESPONSE_DN	H	HALLMARK_COAGULATION+++HALLMARK_COMPLEMENT+++HALLMARK_UV_RESPONSE_DN
253559	'CADM2'	mRNA	HALLMARK_APICAL_JUNCTION	H	HALLMARK_APICAL_JUNCTION
254863	'TMEM256'	mRNA			
256764	'WDR72'	mRNA			
2583	'B4GALNT1'	mRNA			
25841	'ABTB2'	mRNA			
25855	'BRMS1'	mRNA			
25873	'RPL36'	mRNA	HALLMARK_P53_PATHWAY	H	HALLMARK_P53_PATHWAY
25890	'ABI3BP'	mRNA	HALLMARK_EPITHELIAL_MESENCHYMAL_TRANSITION	H	HALLMARK_EPITHELIAL_MESENCHYMAL_TRANSITION
25906	'ANAPC15'	mRNA			
25907	'TMEM158'	mRNA	HALLMARK_KRAS_SIGNALING_UP	H	HALLMARK_KRAS_SIGNALING_UP
259307	'IL4I1'	mRNA	HALLMARK_FATTY_ACID_METABOLISM	H	HALLMARK_FATTY_ACID_METABOLISM
25932	'CLIC4'	mRNA			
26020	'LRP10'	mRNA	HALLMARK_HEME_METABOLISM	H	HALLMARK_HEME_METABOLISM
26022	'TMEM98'	mRNA			
260436	'FDCSP'	mRNA			
26064	'RAI14'	mRNA			
26115	'TANC2'	mRNA			
26227	'PHGDH'	mRNA	HALLMARK_MTORC1_SIGNALING	H	HALLMARK_MTORC1_SIGNALING
26278	'SACS'	mRNA			
2634	'GBP2'	mRNA	HALLMARK_ALLOGRAFT_REJECTION+++HALLMARK_INTERFERON_ALPHA_RESPONSE	H	HALLMARK_ALLOGRAFT_REJECTION+++HALLMARK_INTERFERON_ALPHA_RESPONSE
2635	'GBP3'	mRNA			
26470	'SEZ6L2'	mRNA			
26472	'PPP1R14B'	mRNA			
26517	'TIMM13'	mRNA	HALLMARK_OXIDATIVE_PHOSPHORYLATION	H	HALLMARK_OXIDATIVE_PHOSPHORYLATION
26519	'TIMM10'	mRNA	HALLMARK_OXIDATIVE_PHOSPHORYLATION	H	HALLMARK_OXIDATIVE_PHOSPHORYLATION
26521	'TIMM8B'	mRNA	HALLMARK_OXIDATIVE_PHOSPHORYLATION	H	HALLMARK_OXIDATIVE_PHOSPHORYLATION
2653	'GCSH'	mRNA			
2669	'GEM'	mRNA	HALLMARK_EPITHELIAL_MESENCHYMAL_TRANSITION+++HALLMARK_TNFA_SIGNALING_VIA_NFKB	H	HALLMARK_EPITHELIAL_MESENCHYMAL_TRANSITION+++HALLMARK_TNFA_SIGNALING_VIA_NFKB
2697	'GJA1'	mRNA	HALLMARK_EPITHELIAL_MESENCHYMAL_TRANSITION+++HALLMARK_ESTROGEN_RESPONSE_EARLY+++HALLMARK_UV_RESPONSE_DN	H	HALLMARK_EPITHELIAL_MESENCHYMAL_TRANSITION+++HALLMARK_ESTROGEN_RESPONSE_EARLY+++HALLMARK_UV_RESPONSE_DN
26973	'CHORDC1'	mRNA			
26986	'PABPC1'	mRNA	HALLMARK_MYC_TARGETS_V1+++HALLMARK_PEROXISOME	H	HALLMARK_MYC_TARGETS_V1+++HALLMARK_PEROXISOME
2706	'GJB2'	mRNA			
27063	'ANKRD1'	mRNA			
27069	'GHITM'	mRNA	HALLMARK_ADIPOGENESIS	H	HALLMARK_ADIPOGENESIS
27101	'CACYBP'	mRNA	HALLMARK_MTORC1_SIGNALING	H	HALLMARK_MTORC1_SIGNALING
27122	'DKK3'	mRNA			
27147	'DENND2A'	mRNA			
27250	'PDCD4'	mRNA	HALLMARK_ADIPOGENESIS+++HALLMARK_APOPTOSIS+++HALLMARK_ESTROGEN_RESPONSE_LATE	H	HALLMARK_ADIPOGENESIS+++HALLMARK_APOPTOSIS+++HALLMARK_ESTROGEN_RESPONSE_LATE
27292	'DIMT1'	mRNA			
27335	'EIF3K'	mRNA			
27338	'UBE2S'	mRNA	HALLMARK_E2F_TARGETS+++HALLMARK_G2M_CHECKPOINT	H	HALLMARK_E2F_TARGETS+++HALLMARK_G2M_CHECKPOINT
27346	'TMEM97'	mRNA	HALLMARK_CHOLESTEROL_HOMEOSTASIS+++HALLMARK_MTORC1_SIGNALING+++HALLMARK_MYC_TARGETS_V2+++HALLMARK_XENOBIOTIC_METABOLISM	H	HALLMARK_CHOLESTEROL_HOMEOSTASIS+++HALLMARK_MTORC1_SIGNALING+++HALLMARK_MYC_TARGETS_V2+++HALLMARK_XENOBIOTIC_METABOLISM
27347	'STK39'	mRNA	HALLMARK_ANDROGEN_RESPONSE	H	HALLMARK_ANDROGEN_RESPONSE
27350	'APOBEC3C'	mRNA			
274	'BIN1'	mRNA	HALLMARK_MITOTIC_SPINDLE+++HALLMARK_MYOGENESIS	H	HALLMARK_MITOTIC_SPINDLE+++HALLMARK_MYOGENESIS
2744	'GLS'	mRNA	HALLMARK_UV_RESPONSE_UP	H	HALLMARK_UV_RESPONSE_UP
2787	'GNG5'	mRNA			
2788	'GNG7'	mRNA			
28231	'SLCO4A1'	mRNA			
28232	'SLCO3A1'	mRNA			
284207	'METRNL'	mRNA			
2873	'GPS1'	mRNA			
2876	'GPX1'	mRNA	HALLMARK_APOPTOSIS	H	HALLMARK_APOPTOSIS
2879	'GPX4'	mRNA	HALLMARK_ADIPOGENESIS+++HALLMARK_APOPTOSIS+++HALLMARK_DNA_REPAIR+++HALLMARK_IL2_STAT5_SIGNALING+++HALLMARK_OXIDATIVE_PHOSPHORYLATION+++HALLMARK_REACTIVE_OXYGEN_SPECIES_PATHWAY	H	HALLMARK_ADIPOGENESIS+++HALLMARK_APOPTOSIS+++HALLMARK_DNA_REPAIR+++HALLMARK_IL2_STAT5_SIGNALING+++HALLMARK_OXIDATIVE_PHOSPHORYLATION+++HALLMARK_REACTIVE_OXYGEN_SPECIES_PATHWAY
29094	'LGALSL'	mRNA			
29107	'NXT1'	mRNA	HALLMARK_ESTROGEN_RESPONSE_EARLY+++HALLMARK_ESTROGEN_RESPONSE_LATE	H	HALLMARK_ESTROGEN_RESPONSE_EARLY+++HALLMARK_ESTROGEN_RESPONSE_LATE
2919	'CXCL1'	mRNA	HALLMARK_COMPLEMENT+++HALLMARK_EPITHELIAL_MESENCHYMAL_TRANSITION+++HALLMARK_IL6_JAK_STAT3_SIGNALING+++HALLMARK_TNFA_SIGNALING_VIA_NFKB	H	HALLMARK_COMPLEMENT+++HALLMARK_EPITHELIAL_MESENCHYMAL_TRANSITION+++HALLMARK_IL6_JAK_STAT3_SIGNALING+++HALLMARK_TNFA_SIGNALING_VIA_NFKB
292	'SLC25A5'	mRNA	HALLMARK_OXIDATIVE_PHOSPHORYLATION	H	HALLMARK_OXIDATIVE_PHOSPHORYLATION
2920	'CXCL2'	mRNA	HALLMARK_TNFA_SIGNALING_VIA_NFKB+++HALLMARK_UV_RESPONSE_UP	H	HALLMARK_TNFA_SIGNALING_VIA_NFKB+++HALLMARK_UV_RESPONSE_UP
2950	'GSTP1'	mRNA			
2971	'GTF3A'	mRNA			
2987	'GUK1'	mRNA	HALLMARK_DNA_REPAIR	H	HALLMARK_DNA_REPAIR
29940	'DSE'	mRNA			
29953	'TRHDE'	mRNA			
3005	'H1-0'	mRNA	HALLMARK_ANDROGEN_RESPONSE+++HALLMARK_APOPTOSIS+++HALLMARK_HEME_METABOLISM	H	HALLMARK_ANDROGEN_RESPONSE+++HALLMARK_APOPTOSIS+++HALLMARK_HEME_METABOLISM
3014	'H2AX'	mRNA	HALLMARK_E2F_TARGETS+++HALLMARK_G2M_CHECKPOINT+++HALLMARK_UNFOLDED_PROTEIN_RESPONSE+++HALLMARK_UV_RESPONSE_UP	H	HALLMARK_E2F_TARGETS+++HALLMARK_G2M_CHECKPOINT+++HALLMARK_UNFOLDED_PROTEIN_RESPONSE+++HALLMARK_UV_RESPONSE_UP
302	'ANXA2'	mRNA	HALLMARK_HYPOXIA+++HALLMARK_UV_RESPONSE_DN	H	HALLMARK_HYPOXIA+++HALLMARK_UV_RESPONSE_DN
3020	'H3-3A'	mRNA			
3021	'H3-3B'	mRNA			
30835	'CD209'	mRNA	HALLMARK_APICAL_JUNCTION	H	HALLMARK_APICAL_JUNCTION
3084	'NRG1'	mRNA			
3091	'HIF1A'	mRNA	HALLMARK_ALLOGRAFT_REJECTION+++HALLMARK_G2M_CHECKPOINT+++HALLMARK_INFLAMMATORY_RESPONSE+++HALLMARK_INTERFERON_GAMMA_RESPONSE	H	HALLMARK_ALLOGRAFT_REJECTION+++HALLMARK_G2M_CHECKPOINT+++HALLMARK_INFLAMMATORY_RESPONSE+++HALLMARK_INTERFERON_GAMMA_RESPONSE
3106	'HLA-B'	mRNA	HALLMARK_INTERFERON_GAMMA_RESPONSE	H	HALLMARK_INTERFERON_GAMMA_RESPONSE
3133	'HLA-E'	mRNA	HALLMARK_ALLOGRAFT_REJECTION	H	HALLMARK_ALLOGRAFT_REJECTION
3146	'HMGB1'	mRNA			
3149	'HMGB3'	mRNA	HALLMARK_E2F_TARGETS+++HALLMARK_G2M_CHECKPOINT	H	HALLMARK_E2F_TARGETS+++HALLMARK_G2M_CHECKPOINT
3151	'HMGN2'	mRNA	HALLMARK_G2M_CHECKPOINT	H	HALLMARK_G2M_CHECKPOINT
3157	'HMGCS1'	mRNA	HALLMARK_ANDROGEN_RESPONSE+++HALLMARK_CHOLESTEROL_HOMEOSTASIS+++HALLMARK_FATTY_ACID_METABOLISM+++HALLMARK_MTORC1_SIGNALING	H	HALLMARK_ANDROGEN_RESPONSE+++HALLMARK_CHOLESTEROL_HOMEOSTASIS+++HALLMARK_FATTY_ACID_METABOLISM+++HALLMARK_MTORC1_SIGNALING
316	'AOX1'	mRNA	HALLMARK_XENOBIOTIC_METABOLISM	H	HALLMARK_XENOBIOTIC_METABOLISM
3163	'HMOX2'	mRNA	HALLMARK_REACTIVE_OXYGEN_SPECIES_PATHWAY	H	HALLMARK_REACTIVE_OXYGEN_SPECIES_PATHWAY
3178	'HNRNPA1'	mRNA	HALLMARK_MYC_TARGETS_V1	H	HALLMARK_MYC_TARGETS_V1
3182	'HNRNPAB'	mRNA			
3215	'HOXB5'	mRNA			
3218	'HOXB8'	mRNA			
3219	'HOXB9'	mRNA	HALLMARK_HYPOXIA	H	HALLMARK_HYPOXIA
3251	'HPRT1'	mRNA	HALLMARK_DNA_REPAIR+++HALLMARK_ESTROGEN_RESPONSE_LATE+++HALLMARK_MTORC1_SIGNALING+++HALLMARK_MYC_TARGETS_V1+++HALLMARK_XENOBIOTIC_METABOLISM	H	HALLMARK_DNA_REPAIR+++HALLMARK_ESTROGEN_RESPONSE_LATE+++HALLMARK_MTORC1_SIGNALING+++HALLMARK_MYC_TARGETS_V1+++HALLMARK_XENOBIOTIC_METABOLISM
330	'BIRC3'	mRNA	HALLMARK_APOPTOSIS+++HALLMARK_KRAS_SIGNALING_UP+++HALLMARK_TNFA_SIGNALING_VIA_NFKB	H	HALLMARK_APOPTOSIS+++HALLMARK_KRAS_SIGNALING_UP+++HALLMARK_TNFA_SIGNALING_VIA_NFKB
3301	'DNAJA1'	mRNA	HALLMARK_APOPTOSIS+++HALLMARK_UV_RESPONSE_UP	H	HALLMARK_APOPTOSIS+++HALLMARK_UV_RESPONSE_UP
3303	'HSPA1A'	mRNA	HALLMARK_COMPLEMENT	H	HALLMARK_COMPLEMENT
3304	'HSPA1B'	mRNA			
3312	'HSPA8'	mRNA	HALLMARK_G2M_CHECKPOINT	H	HALLMARK_G2M_CHECKPOINT
3313	'HSPA9'	mRNA	HALLMARK_MTORC1_SIGNALING+++HALLMARK_OXIDATIVE_PHOSPHORYLATION+++HALLMARK_UNFOLDED_PROTEIN_RESPONSE	H	HALLMARK_MTORC1_SIGNALING+++HALLMARK_OXIDATIVE_PHOSPHORYLATION+++HALLMARK_UNFOLDED_PROTEIN_RESPONSE
3320	'HSP90AA1'	mRNA	HALLMARK_FATTY_ACID_METABOLISM	H	HALLMARK_FATTY_ACID_METABOLISM
3326	'HSP90AB1'	mRNA	HALLMARK_MYC_TARGETS_V1	H	HALLMARK_MYC_TARGETS_V1
3329	'HSPD1'	mRNA	HALLMARK_MTORC1_SIGNALING+++HALLMARK_MYC_TARGETS_V1+++HALLMARK_MYC_TARGETS_V2	H	HALLMARK_MTORC1_SIGNALING+++HALLMARK_MYC_TARGETS_V1+++HALLMARK_MYC_TARGETS_V2
3336	'HSPE1'	mRNA	HALLMARK_MTORC1_SIGNALING+++HALLMARK_MYC_TARGETS_V1+++HALLMARK_MYC_TARGETS_V2	H	HALLMARK_MTORC1_SIGNALING+++HALLMARK_MYC_TARGETS_V1+++HALLMARK_MYC_TARGETS_V2
3337	'DNAJB1'	mRNA	HALLMARK_UV_RESPONSE_UP	H	HALLMARK_UV_RESPONSE_UP
3383	'ICAM1'	mRNA	HALLMARK_ALLOGRAFT_REJECTION+++HALLMARK_APICAL_JUNCTION+++HALLMARK_INFLAMMATORY_RESPONSE+++HALLMARK_INTERFERON_GAMMA_RESPONSE+++HALLMARK_TNFA_SIGNALING_VIA_NFKB+++HALLMARK_UV_RESPONSE_UP	H	HALLMARK_ALLOGRAFT_REJECTION+++HALLMARK_APICAL_JUNCTION+++HALLMARK_INFLAMMATORY_RESPONSE+++HALLMARK_INTERFERON_GAMMA_RESPONSE+++HALLMARK_TNFA_SIGNALING_VIA_NFKB+++HALLMARK_UV_RESPONSE_UP
3399	'ID3'	mRNA	HALLMARK_TGF_BETA_SIGNALING	H	HALLMARK_TGF_BETA_SIGNALING
340061	'STING1'	mRNA			
340273	'ABCB5'	mRNA			
3429	'IFI27'	mRNA	HALLMARK_INTERFERON_ALPHA_RESPONSE+++HALLMARK_INTERFERON_GAMMA_RESPONSE	H	HALLMARK_INTERFERON_ALPHA_RESPONSE+++HALLMARK_INTERFERON_GAMMA_RESPONSE
3433	'IFIT2'	mRNA	HALLMARK_INTERFERON_ALPHA_RESPONSE+++HALLMARK_INTERFERON_GAMMA_RESPONSE+++HALLMARK_TNFA_SIGNALING_VIA_NFKB	H	HALLMARK_INTERFERON_ALPHA_RESPONSE+++HALLMARK_INTERFERON_GAMMA_RESPONSE+++HALLMARK_TNFA_SIGNALING_VIA_NFKB
3455	'IFNAR2'	mRNA	HALLMARK_ALLOGRAFT_REJECTION+++HALLMARK_INTERFERON_GAMMA_RESPONSE	H	HALLMARK_ALLOGRAFT_REJECTION+++HALLMARK_INTERFERON_GAMMA_RESPONSE
347735	'SERINC2'	mRNA			
3481	'IGF2'	mRNA	HALLMARK_KRAS_SIGNALING_UP	H	HALLMARK_KRAS_SIGNALING_UP
3488	'IGFBP5'	mRNA	HALLMARK_MTORC1_SIGNALING+++HALLMARK_UV_RESPONSE_DN	H	HALLMARK_MTORC1_SIGNALING+++HALLMARK_UV_RESPONSE_DN
3491	'CCN1'	mRNA	HALLMARK_EPITHELIAL_MESENCHYMAL_TRANSITION+++HALLMARK_HYPOXIA+++HALLMARK_TNFA_SIGNALING_VIA_NFKB+++HALLMARK_UV_RESPONSE_DN	H	HALLMARK_EPITHELIAL_MESENCHYMAL_TRANSITION+++HALLMARK_HYPOXIA+++HALLMARK_TNFA_SIGNALING_VIA_NFKB+++HALLMARK_UV_RESPONSE_DN
353	'APRT'	mRNA	HALLMARK_DNA_REPAIR	H	HALLMARK_DNA_REPAIR
3552	'IL1A'	mRNA	HALLMARK_APOPTOSIS+++HALLMARK_INFLAMMATORY_RESPONSE+++HALLMARK_P53_PATHWAY+++HALLMARK_TNFA_SIGNALING_VIA_NFKB	H	HALLMARK_APOPTOSIS+++HALLMARK_INFLAMMATORY_RESPONSE+++HALLMARK_P53_PATHWAY+++HALLMARK_TNFA_SIGNALING_VIA_NFKB
3553	'IL1B'	mRNA	HALLMARK_ALLOGRAFT_REJECTION+++HALLMARK_APOPTOSIS+++HALLMARK_IL6_JAK_STAT3_SIGNALING+++HALLMARK_INFLAMMATORY_RESPONSE+++HALLMARK_KRAS_SIGNALING_UP+++HALLMARK_TNFA_SIGNALING_VIA_NFKB	H	HALLMARK_ALLOGRAFT_REJECTION+++HALLMARK_APOPTOSIS+++HALLMARK_IL6_JAK_STAT3_SIGNALING+++HALLMARK_INFLAMMATORY_RESPONSE+++HALLMARK_KRAS_SIGNALING_UP+++HALLMARK_TNFA_SIGNALING_VIA_NFKB
3570	'IL6R'	mRNA			
3604	'TNFRSF9'	mRNA	HALLMARK_IL2_STAT5_SIGNALING+++HALLMARK_INFLAMMATORY_RESPONSE+++HALLMARK_TNFA_SIGNALING_VIA_NFKB	H	HALLMARK_IL2_STAT5_SIGNALING+++HALLMARK_INFLAMMATORY_RESPONSE+++HALLMARK_TNFA_SIGNALING_VIA_NFKB
3606	'IL18'	mRNA	HALLMARK_ALLOGRAFT_REJECTION+++HALLMARK_APOPTOSIS+++HALLMARK_INFLAMMATORY_RESPONSE+++HALLMARK_TNFA_SIGNALING_VIA_NFKB	H	HALLMARK_ALLOGRAFT_REJECTION+++HALLMARK_APOPTOSIS+++HALLMARK_INFLAMMATORY_RESPONSE+++HALLMARK_TNFA_SIGNALING_VIA_NFKB
3625	'INHBB'	mRNA	HALLMARK_ALLOGRAFT_REJECTION+++HALLMARK_ESTROGEN_RESPONSE_EARLY+++HALLMARK_P53_PATHWAY	H	HALLMARK_ALLOGRAFT_REJECTION+++HALLMARK_ESTROGEN_RESPONSE_EARLY+++HALLMARK_P53_PATHWAY
3656	'IRAK2'	mRNA	HALLMARK_INFLAMMATORY_RESPONSE	H	HALLMARK_INFLAMMATORY_RESPONSE
3669	'ISG20'	mRNA	HALLMARK_APOPTOSIS+++HALLMARK_ESTROGEN_RESPONSE_LATE+++HALLMARK_GLYCOLYSIS+++HALLMARK_HYPOXIA+++HALLMARK_INTERFERON_ALPHA_RESPONSE+++HALLMARK_INTERFERON_GAMMA_RESPONSE	H	HALLMARK_APOPTOSIS+++HALLMARK_ESTROGEN_RESPONSE_LATE+++HALLMARK_GLYCOLYSIS+++HALLMARK_HYPOXIA+++HALLMARK_INTERFERON_ALPHA_RESPONSE+++HALLMARK_INTERFERON_GAMMA_RESPONSE
3675	'ITGA3'	mRNA	HALLMARK_APICAL_JUNCTION	H	HALLMARK_APICAL_JUNCTION
3690	'ITGB3'	mRNA	HALLMARK_COAGULATION+++HALLMARK_EPITHELIAL_MESENCHYMAL_TRANSITION+++HALLMARK_IL6_JAK_STAT3_SIGNALING+++HALLMARK_INFLAMMATORY_RESPONSE+++HALLMARK_UV_RESPONSE_DN	H	HALLMARK_COAGULATION+++HALLMARK_EPITHELIAL_MESENCHYMAL_TRANSITION+++HALLMARK_IL6_JAK_STAT3_SIGNALING+++HALLMARK_INFLAMMATORY_RESPONSE+++HALLMARK_UV_RESPONSE_DN
3710	'ITPR3'	mRNA			
3732	'CD82'	mRNA	HALLMARK_INFLAMMATORY_RESPONSE+++HALLMARK_P53_PATHWAY	H	HALLMARK_INFLAMMATORY_RESPONSE+++HALLMARK_P53_PATHWAY
381	'ARF5'	mRNA			
3814	'KISS1'	mRNA			
3838	'KPNA2'	mRNA	HALLMARK_E2F_TARGETS+++HALLMARK_G2M_CHECKPOINT+++HALLMARK_MYC_TARGETS_V1	H	HALLMARK_E2F_TARGETS+++HALLMARK_G2M_CHECKPOINT+++HALLMARK_MYC_TARGETS_V1
3843	'IPO5'	mRNA			
3856	'KRT8'	mRNA	HALLMARK_ANDROGEN_RESPONSE+++HALLMARK_ESTROGEN_RESPONSE_EARLY	H	HALLMARK_ANDROGEN_RESPONSE+++HALLMARK_ESTROGEN_RESPONSE_EARLY
388524	'RPSA2'	mRNA			
388962	'BOLA3'	mRNA			
3897	'L1CAM'	mRNA	HALLMARK_HEDGEHOG_SIGNALING	H	HALLMARK_HEDGEHOG_SIGNALING
390792	'KRT39'	mRNA			
391	'RHOG'	mRNA	HALLMARK_COMPLEMENT+++HALLMARK_INFLAMMATORY_RESPONSE	H	HALLMARK_COMPLEMENT+++HALLMARK_INFLAMMATORY_RESPONSE
3914	'LAMB3'	mRNA	HALLMARK_APICAL_JUNCTION+++HALLMARK_TNFA_SIGNALING_VIA_NFKB	H	HALLMARK_APICAL_JUNCTION+++HALLMARK_TNFA_SIGNALING_VIA_NFKB
3918	'LAMC2'	mRNA	HALLMARK_APICAL_JUNCTION+++HALLMARK_EPITHELIAL_MESENCHYMAL_TRANSITION+++HALLMARK_ESTROGEN_RESPONSE_LATE	H	HALLMARK_APICAL_JUNCTION+++HALLMARK_EPITHELIAL_MESENCHYMAL_TRANSITION+++HALLMARK_ESTROGEN_RESPONSE_LATE
3921	'RPSA'	mRNA			
394	'ARHGAP5'	mRNA	HALLMARK_MITOTIC_SPINDLE	H	HALLMARK_MITOTIC_SPINDLE

3945	'LDHB'	mRNA	HALLMARK_OXIDATIVE_PHOSPHORYLATION+++HALLMARK_P53_PATHWAY	H	HALLMARK_OXIDATIVE_PHOSPHORYLATION+++HALLMARK_P53_PATHWAY
397	'ARHGDIB'	mRNA			
3983	'ABLIM1'	mRNA	HALLMARK_ESTROGEN_RESPONSE_EARLY+++HALLMARK_MYOGENESIS	H	HALLMARK_ESTROGEN_RESPONSE_EARLY+++HALLMARK_MYOGENESIS
4001	'LMNB1'	mRNA	HALLMARK_E2F_TARGETS+++HALLMARK_G2M_CHECKPOINT+++HALLMARK_MITOTIC_SPINDLE	H	HALLMARK_E2F_TARGETS+++HALLMARK_G2M_CHECKPOINT+++HALLMARK_MITOTIC_SPINDLE
400745	'SH2D5'	mRNA			
401138	'AMTN'	mRNA			
4013	'VWA5A'	mRNA	HALLMARK_KRAS_SIGNALING_UP+++HALLMARK_P53_PATHWAY	H	HALLMARK_KRAS_SIGNALING_UP+++HALLMARK_P53_PATHWAY
401427	'OR2A7'	mRNA			
4015	'LOX'	mRNA	HALLMARK_EPITHELIAL_MESENCHYMAL_TRANSITION+++HALLMARK_HYPOXIA	H	HALLMARK_EPITHELIAL_MESENCHYMAL_TRANSITION+++HALLMARK_HYPOXIA
401505	'TOMM5'	mRNA			
4035	'LRP1'	mRNA	HALLMARK_COAGULATION+++HALLMARK_COMPLEMENT+++HALLMARK_EPITHELIAL_MESENCHYMAL_TRANSITION	H	HALLMARK_COAGULATION+++HALLMARK_COMPLEMENT+++HALLMARK_EPITHELIAL_MESENCHYMAL_TRANSITION
4131	'MAP1B'	mRNA	HALLMARK_UV_RESPONSE_DN	H	HALLMARK_UV_RESPONSE_DN
4134	'MAP4'	mRNA			
4150	'MAZ'	mRNA			
4172	'MCM3'	mRNA	HALLMARK_E2F_TARGETS+++HALLMARK_G2M_CHECKPOINT	H	HALLMARK_E2F_TARGETS+++HALLMARK_G2M_CHECKPOINT
4176	'MCM7'	mRNA	HALLMARK_E2F_TARGETS+++HALLMARK_EPITHELIAL_MESENCHYMAL_TRANSITION+++HALLMARK_MYC_TARGETS_V1	H	HALLMARK_E2F_TARGETS+++HALLMARK_EPITHELIAL_MESENCHYMAL_TRANSITION+++HALLMARK_MYC_TARGETS_V1
4189	'DNAJB9'	mRNA	HALLMARK_ADIPOGENESIS+++HALLMARK_ANDROGEN_RESPONSE+++HALLMARK_UNFOLDED_PROTEIN_RESPONSE	H	HALLMARK_ADIPOGENESIS+++HALLMARK_ANDROGEN_RESPONSE+++HALLMARK_UNFOLDED_PROTEIN_RESPONSE
4240	'MFGE8'	mRNA			
4241	'MELTF'	mRNA			
4254	'KITLG'	mRNA			
4311	'MME'	mRNA			
4312	'MMP1'	mRNA	HALLMARK_COAGULATION+++HALLMARK_EPITHELIAL_MESENCHYMAL_TRANSITION	H	HALLMARK_COAGULATION+++HALLMARK_EPITHELIAL_MESENCHYMAL_TRANSITION
4319	'MMP10'	mRNA	HALLMARK_COAGULATION+++HALLMARK_KRAS_SIGNALING_UP	H	HALLMARK_COAGULATION+++HALLMARK_KRAS_SIGNALING_UP
4322	'MMP13'	mRNA	HALLMARK_COMPLEMENT	H	HALLMARK_COMPLEMENT
4325	'MMP16'	mRNA	HALLMARK_UV_RESPONSE_DN	H	HALLMARK_UV_RESPONSE_DN
439	'GET3'	mRNA			
444	'ASPH'	mRNA			
445	'ASS1'	mRNA	HALLMARK_ESTROGEN_RESPONSE_LATE	H	HALLMARK_ESTROGEN_RESPONSE_LATE
445815	'PALM2AKAP2'	mRNA			
4493	'MT1E'	mRNA	HALLMARK_HYPOXIA+++HALLMARK_UV_RESPONSE_DN	H	HALLMARK_HYPOXIA+++HALLMARK_UV_RESPONSE_DN
4599	'MX1'	mRNA	HALLMARK_INTERFERON_ALPHA_RESPONSE+++HALLMARK_INTERFERON_GAMMA_RESPONSE+++HALLMARK_KRAS_SIGNALING_DN	H	HALLMARK_INTERFERON_ALPHA_RESPONSE+++HALLMARK_INTERFERON_GAMMA_RESPONSE+++HALLMARK_KRAS_SIGNALING_DN
4605	'MYBL2'	mRNA	HALLMARK_E2F_TARGETS+++HALLMARK_G2M_CHECKPOINT	H	HALLMARK_E2F_TARGETS+++HALLMARK_G2M_CHECKPOINT
4609	'MYC'	mRNA	HALLMARK_E2F_TARGETS+++HALLMARK_ESTROGEN_RESPONSE_EARLY+++HALLMARK_G2M_CHECKPOINT+++HALLMARK_IL2_STAT5_SIGNALING+++HALLMARK_INFLAMMATORY_RESPONSE+++HALLMARK_MYC_TARGETS_V1+++HALLMARK_MYC_TARGETS_V2+++HALLMARK_TNFA_SIGNALING_VIA_NFKB+++HALLMARK_UV_RESPONSE_DN+++HALLMARK_WNT_BETA_CATENIN_SIGNALING	H	HALLMARK_E2F_TARGETS+++HALLMARK_ESTROGEN_RESPONSE_EARLY+++HALLMARK_G2M_CHECKPOINT+++HALLMARK_IL2_STAT5_SIGNALING+++HALLMARK_INFLAMMATORY_RESPONSE+++HALLMARK_MYC_TARGETS_V1+++HALLMARK_MYC_TARGETS_V2+++HALLMARK_TNFA_SIGNALING_VIA_NFKB+++HALLMARK_UV_RESPONSE_DN+++HALLMARK_WNT_BETA_CATENIN_SIGNALING
4638	'MYLK'	mRNA	HALLMARK_ADIPOGENESIS+++HALLMARK_EPITHELIAL_MESENCHYMAL_TRANSITION+++HALLMARK_MYOGENESIS	H	HALLMARK_ADIPOGENESIS+++HALLMARK_EPITHELIAL_MESENCHYMAL_TRANSITION+++HALLMARK_MYOGENESIS
4642	'MYO1D'	mRNA			
4666	'NACA'	mRNA			
4685	'NCAM2'	mRNA			
4694	'NDUFA1'	mRNA	HALLMARK_OXIDATIVE_PHOSPHORYLATION	H	HALLMARK_OXIDATIVE_PHOSPHORYLATION
4722	'NDUFS3'	mRNA	HALLMARK_ADIPOGENESIS+++HALLMARK_OXIDATIVE_PHOSPHORYLATION	H	HALLMARK_ADIPOGENESIS+++HALLMARK_OXIDATIVE_PHOSPHORYLATION
4726	'NDUFS6'	mRNA	HALLMARK_OXIDATIVE_PHOSPHORYLATION	H	HALLMARK_OXIDATIVE_PHOSPHORYLATION
4728	'NDUFS8'	mRNA	HALLMARK_OXIDATIVE_PHOSPHORYLATION	H	HALLMARK_OXIDATIVE_PHOSPHORYLATION
4736	'RPL10A'	mRNA			
4756	'NEO1'	mRNA			
4784	'NFIX'	mRNA			
4792	'NFKBIA'	mRNA	HALLMARK_INFLAMMATORY_RESPONSE+++HALLMARK_INTERFERON_GAMMA_RESPONSE+++HALLMARK_TNFA_SIGNALING_VIA_NFKB+++HALLMARK_UV_RESPONSE_UP	H	HALLMARK_INFLAMMATORY_RESPONSE+++HALLMARK_INTERFERON_GAMMA_RESPONSE+++HALLMARK_TNFA_SIGNALING_VIA_NFKB+++HALLMARK_UV_RESPONSE_UP
4811	'NID1'	mRNA			
4830	'NME1'	mRNA	HALLMARK_ALLOGRAFT_REJECTION+++HALLMARK_DNA_REPAIR+++HALLMARK_E2F_TARGETS+++HALLMARK_MYC_TARGETS_V1	H	HALLMARK_ALLOGRAFT_REJECTION+++HALLMARK_DNA_REPAIR+++HALLMARK_E2F_TARGETS+++HALLMARK_MYC_TARGETS_V1
4837	'NNMT'	mRNA	HALLMARK_EPITHELIAL_MESENCHYMAL_TRANSITION	H	HALLMARK_EPITHELIAL_MESENCHYMAL_TRANSITION
4839	'NOP2'	mRNA	HALLMARK_IL2_STAT5_SIGNALING+++HALLMARK_MYC_TARGETS_V2	H	HALLMARK_IL2_STAT5_SIGNALING+++HALLMARK_MYC_TARGETS_V2
4862	'NPAS2'	mRNA			
4864	'NPC1'	mRNA	HALLMARK_BILE_ACID_METABOLISM+++HALLMARK_XENOBIOTIC_METABOLISM	H	HALLMARK_BILE_ACID_METABOLISM+++HALLMARK_XENOBIOTIC_METABOLISM
4869	'NPM1'	mRNA	HALLMARK_ALLOGRAFT_REJECTION+++HALLMARK_MYC_TARGETS_V1+++HALLMARK_MYC_TARGETS_V2+++HALLMARK_UNFOLDED_PROTEIN_RESPONSE	H	HALLMARK_ALLOGRAFT_REJECTION+++HALLMARK_MYC_TARGETS_V1+++HALLMARK_MYC_TARGETS_V2+++HALLMARK_UNFOLDED_PROTEIN_RESPONSE
490	'ATP2B1'	mRNA	HALLMARK_INFLAMMATORY_RESPONSE+++HALLMARK_TNFA_SIGNALING_VIA_NFKB+++HALLMARK_UV_RESPONSE_DN	H	HALLMARK_INFLAMMATORY_RESPONSE+++HALLMARK_TNFA_SIGNALING_VIA_NFKB+++HALLMARK_UV_RESPONSE_DN
4904	'YBX1'	mRNA			
4907	'NT5E'	mRNA	HALLMARK_EPITHELIAL_MESENCHYMAL_TRANSITION+++HALLMARK_GLYCOLYSIS+++HALLMARK_IL2_STAT5_SIGNALING	H	HALLMARK_EPITHELIAL_MESENCHYMAL_TRANSITION+++HALLMARK_GLYCOLYSIS+++HALLMARK_IL2_STAT5_SIGNALING
4925	'NUCB2'	mRNA			
493	'ATP2B4'	mRNA	HALLMARK_ESTROGEN_RESPONSE_LATE+++HALLMARK_UV_RESPONSE_DN	H	HALLMARK_ESTROGEN_RESPONSE_LATE+++HALLMARK_UV_RESPONSE_DN
4939	'OAS2'	mRNA	HALLMARK_INTERFERON_GAMMA_RESPONSE	H	HALLMARK_INTERFERON_GAMMA_RESPONSE
4953	'ODC1'	mRNA	HALLMARK_FATTY_ACID_METABOLISM+++HALLMARK_G2M_CHECKPOINT+++HALLMARK_IL2_STAT5_SIGNALING+++HALLMARK_MYC_TARGETS_V1	H	HALLMARK_FATTY_ACID_METABOLISM+++HALLMARK_G2M_CHECKPOINT+++HALLMARK_IL2_STAT5_SIGNALING+++HALLMARK_MYC_TARGETS_V1
4982	'TNFRSF11B'	mRNA	HALLMARK_APICAL_JUNCTION+++HALLMARK_EPITHELIAL_MESENCHYMAL_TRANSITION	H	HALLMARK_APICAL_JUNCTION+++HALLMARK_EPITHELIAL_MESENCHYMAL_TRANSITION
5036	'PA2G4'	mRNA	HALLMARK_ANDROGEN_RESPONSE+++HALLMARK_E2F_TARGETS+++HALLMARK_MYC_TARGETS_V1+++HALLMARK_MYC_TARGETS_V2	H	HALLMARK_ANDROGEN_RESPONSE+++HALLMARK_E2F_TARGETS+++HALLMARK_MYC_TARGETS_V1+++HALLMARK_MYC_TARGETS_V2
50486	'G0S2'	mRNA	HALLMARK_FATTY_ACID_METABOLISM+++HALLMARK_KRAS_SIGNALING_UP+++HALLMARK_TNFA_SIGNALING_VIA_NFKB	H	HALLMARK_FATTY_ACID_METABOLISM+++HALLMARK_KRAS_SIGNALING_UP+++HALLMARK_TNFA_SIGNALING_VIA_NFKB
50488	'MINK1'	mRNA			
5052	'PRDX1'	mRNA	HALLMARK_MTORC1_SIGNALING+++HALLMARK_PEROXISOME+++HALLMARK_REACTIVE_OXYGEN_SPECIES_PATHWAY	H	HALLMARK_MTORC1_SIGNALING+++HALLMARK_PEROXISOME+++HALLMARK_REACTIVE_OXYGEN_SPECIES_PATHWAY
5055	'SERPINB2'	mRNA	HALLMARK_COAGULATION+++HALLMARK_COMPLEMENT+++HALLMARK_KRAS_SIGNALING_DN+++HALLMARK_TNFA_SIGNALING_VIA_NFKB	H	HALLMARK_COAGULATION+++HALLMARK_COMPLEMENT+++HALLMARK_KRAS_SIGNALING_DN+++HALLMARK_TNFA_SIGNALING_VIA_NFKB
506	'ATP5F1B'	mRNA	HALLMARK_OXIDATIVE_PHOSPHORYLATION	H	HALLMARK_OXIDATIVE_PHOSPHORYLATION
50628	'GEMIN4'	mRNA	HALLMARK_MITOTIC_SPINDLE+++HALLMARK_UNFOLDED_PROTEIN_RESPONSE	H	HALLMARK_MITOTIC_SPINDLE+++HALLMARK_UNFOLDED_PROTEIN_RESPONSE
5066	'PAM'	mRNA	HALLMARK_GLYCOLYSIS+++HALLMARK_HYPOXIA+++HALLMARK_PROTEIN_SECRETION	H	HALLMARK_GLYCOLYSIS+++HALLMARK_HYPOXIA+++HALLMARK_PROTEIN_SECRETION
5092	'PCBD1'	mRNA	HALLMARK_FATTY_ACID_METABOLISM	H	HALLMARK_FATTY_ACID_METABOLISM
50944	'SHANK1'	mRNA			
5098	'PCDHGC3'	mRNA			
51002	'TPRKB'	mRNA	HALLMARK_P53_PATHWAY	H	HALLMARK_P53_PATHWAY
51073	'MRPL4'	mRNA			
51081	'MRPS7'	mRNA			
51109	'RDH11'	mRNA	HALLMARK_FATTY_ACID_METABOLISM+++HALLMARK_MTORC1_SIGNALING+++HALLMARK_PEROXISOME	H	HALLMARK_FATTY_ACID_METABOLISM+++HALLMARK_MTORC1_SIGNALING+++HALLMARK_PEROXISOME
5111	'PCNA'	mRNA	HALLMARK_DNA_REPAIR+++HALLMARK_E2F_TARGETS+++HALLMARK_MYC_TARGETS_V1+++HALLMARK_P53_PATHWAY	H	HALLMARK_DNA_REPAIR+++HALLMARK_E2F_TARGETS+++HALLMARK_MYC_TARGETS_V1+++HALLMARK_P53_PATHWAY
51154	'MRTO4'	mRNA	HALLMARK_MYC_TARGETS_V2	H	HALLMARK_MYC_TARGETS_V2
51200	'CPA4'	mRNA			
51213	'LUZP4'	mRNA			
51218	'GLRX5'	mRNA	HALLMARK_HEME_METABOLISM	H	HALLMARK_HEME_METABOLISM
51232	'CRIM1'	mRNA			
51258	'MRPL51'	mRNA			
51264	'MRPL27'	mRNA			
51287	'COA4'	mRNA			
513	'ATP5F1D'	mRNA	HALLMARK_OXIDATIVE_PHOSPHORYLATION	H	HALLMARK_OXIDATIVE_PHOSPHORYLATION
51330	'TNFRSF12A'	mRNA	HALLMARK_APOPTOSIS+++HALLMARK_CHOLESTEROL_HOMEOSTASIS+++HALLMARK_EPITHELIAL_MESENCHYMAL_TRANSITION+++HALLMARK_IL6_JAK_STAT3_SIGNALING	H	HALLMARK_APOPTOSIS+++HALLMARK_CHOLESTEROL_HOMEOSTASIS+++HALLMARK_EPITHELIAL_MESENCHYMAL_TRANSITION+++HALLMARK_IL6_JAK_STAT3_SIGNALING
51363	'CHST15'	mRNA			
51371	'POMP'	mRNA			
51372	'TMA7'	mRNA			
5138	'PDE2A'	mRNA			
51399	'TRAPPC4'	mRNA			
514	'ATP5F1E'	mRNA	HALLMARK_OXIDATIVE_PHOSPHORYLATION	H	HALLMARK_OXIDATIVE_PHOSPHORYLATION
51406	'NOL7'	mRNA			
51422	'PRKAG2'	mRNA			
51428	'DDX41'	mRNA			
51444	'RNF138'	mRNA			
51504	'TRMT112'	mRNA			
51560	'RAB6B'	mRNA			
516	'ATP5MC1'	mRNA	HALLMARK_MTORC1_SIGNALING+++HALLMARK_OXIDATIVE_PHOSPHORYLATION	H	HALLMARK_MTORC1_SIGNALING+++HALLMARK_OXIDATIVE_PHOSPHORYLATION
5163	'PDK1'	mRNA	HALLMARK_HYPOXIA+++HALLMARK_MTORC1_SIGNALING+++HALLMARK_PI3K_AKT_MTOR_SIGNALING	H	HALLMARK_HYPOXIA+++HALLMARK_MTORC1_SIGNALING+++HALLMARK_PI3K_AKT_MTOR_SIGNALING
51646	'YPEL5'	mRNA	HALLMARK_HEME_METABOLISM	H	HALLMARK_HEME_METABOLISM
51655	'RASD1'	mRNA			
51659	'GINS2'	mRNA	HALLMARK_ESTROGEN_RESPONSE_LATE+++HALLMARK_G2M_CHECKPOINT	H	HALLMARK_ESTROGEN_RESPONSE_LATE+++HALLMARK_G2M_CHECKPOINT
51690	'LSM7'	mRNA	HALLMARK_MYC_TARGETS_V1	H	HALLMARK_MYC_TARGETS_V1
51704	'GPRC5B'	mRNA	HALLMARK_KRAS_SIGNALING_UP	H	HALLMARK_KRAS_SIGNALING_UP
51776	'MAP3K20'	mRNA	HALLMARK_APICAL_JUNCTION+++HALLMARK_G2M_CHECKPOINT	H	HALLMARK_APICAL_JUNCTION+++HALLMARK_G2M_CHECKPOINT
518	'ATP5MC3'	mRNA	HALLMARK_OXIDATIVE_PHOSPHORYLATION	H	HALLMARK_OXIDATIVE_PHOSPHORYLATION
5245	'PHB1'	mRNA	HALLMARK_MYC_TARGETS_V1+++HALLMARK_MYC_TARGETS_V2	H	HALLMARK_MYC_TARGETS_V1+++HALLMARK_MYC_TARGETS_V2
5250	'SLC25A3'	mRNA	HALLMARK_MYC_TARGETS_V1+++HALLMARK_OXIDATIVE_PHOSPHORYLATION	H	HALLMARK_MYC_TARGETS_V1+++HALLMARK_OXIDATIVE_PHOSPHORYLATION
5266	'PI3'	mRNA			
5270	'SERPINE2'	mRNA	HALLMARK_EPITHELIAL_MESENCHYMAL_TRANSITION	H	HALLMARK_EPITHELIAL_MESENCHYMAL_TRANSITION
5327	'PLAT'	mRNA	HALLMARK_APOPTOSIS+++HALLMARK_COAGULATION+++HALLMARK_COMPLEMENT+++HALLMARK_KRAS_SIGNALING_UP	H	HALLMARK_APOPTOSIS+++HALLMARK_COAGULATION+++HALLMARK_COMPLEMENT+++HALLMARK_KRAS_SIGNALING_UP
5331	'PLCB3'	mRNA			
5332	'PLCB4'	mRNA	HALLMARK_UV_RESPONSE_DN	H	HALLMARK_UV_RESPONSE_DN
5336	'PLCG2'	mRNA			
53373	'TPCN1'	mRNA			
5347	'PLK1'	mRNA	HALLMARK_E2F_TARGETS+++HALLMARK_G2M_CHECKPOINT+++HALLMARK_MITOTIC_SPINDLE+++HALLMARK_MTORC1_SIGNALING+++HALLMARK_MYC_TARGETS_V2	H	HALLMARK_E2F_TARGETS+++HALLMARK_G2M_CHECKPOINT+++HALLMARK_MITOTIC_SPINDLE+++HALLMARK_MTORC1_SIGNALING+++HALLMARK_MYC_TARGETS_V2
5352	'PLOD2'	mRNA	HALLMARK_EPITHELIAL_MESENCHYMAL_TRANSITION+++HALLMARK_GLYCOLYSIS+++HALLMARK_MTORC1_SIGNALING	H	HALLMARK_EPITHELIAL_MESENCHYMAL_TRANSITION+++HALLMARK_GLYCOLYSIS+++HALLMARK_MTORC1_SIGNALING
53615	'MBD3'	mRNA			
5362	'PLXNA2'	mRNA			
53840	'TRIM34'	mRNA			
53947	'A4GALT'	mRNA			
54107	'POLE3'	mRNA	HALLMARK_MYC_TARGETS_V1+++HALLMARK_UV_RESPONSE_UP	H	HALLMARK_MYC_TARGETS_V1+++HALLMARK_UV_RESPONSE_UP
5413	'SEPTIN5'	mRNA			
54206	'ERRFI1'	mRNA	HALLMARK_CHOLESTEROL_HOMEOSTASIS+++HALLMARK_HYPOXIA	H	HALLMARK_CHOLESTEROL_HOMEOSTASIS+++HALLMARK_HYPOXIA
5425	'POLD2'	mRNA	HALLMARK_E2F_TARGETS+++HALLMARK_MYC_TARGETS_V1	H	HALLMARK_E2F_TARGETS+++HALLMARK_MYC_TARGETS_V1
54331	'GNG2'	mRNA	HALLMARK_COMPLEMENT	H	HALLMARK_COMPLEMENT
5434	'POLR2E'	mRNA	HALLMARK_DNA_REPAIR	H	HALLMARK_DNA_REPAIR
5436	'POLR2G'	mRNA	HALLMARK_DNA_REPAIR	H	HALLMARK_DNA_REPAIR
5441	'POLR2L'	mRNA			
54458	'PRR13'	mRNA			
54475	'NLE1'	mRNA			
54541	'DDIT4'	mRNA	HALLMARK_GLYCOLYSIS+++HALLMARK_HYPOXIA+++HALLMARK_MTORC1_SIGNALING+++HALLMARK_P53_PATHWAY+++HALLMARK_UNFOLDED_PROTEIN_RESPONSE	H	HALLMARK_GLYCOLYSIS+++HALLMARK_HYPOXIA+++HALLMARK_MTORC1_SIGNALING+++HALLMARK_P53_PATHWAY+++HALLMARK_UNFOLDED_PROTEIN_RESPONSE
54555	'DDX49'	mRNA			
54663	'WDR74'	mRNA	HALLMARK_MYC_TARGETS_V2	H	HALLMARK_MYC_TARGETS_V2
54675	'CRLS1'	mRNA			
5468	'PPARG'	mRNA	HALLMARK_ADIPOGENESIS+++HALLMARK_CHOLESTEROL_HOMEOSTASIS+++HALLMARK_UV_RESPONSE_DN	H	HALLMARK_ADIPOGENESIS+++HALLMARK_CHOLESTEROL_HOMEOSTASIS+++HALLMARK_UV_RESPONSE_DN
54739	'XAF1'	mRNA	HALLMARK_INTERFERON_GAMMA_RESPONSE	H	HALLMARK_INTERFERON_GAMMA_RESPONSE
54800	'KLHL24'	mRNA	HALLMARK_HYPOXIA	H	HALLMARK_HYPOXIA
5493	'PPL'	mRNA			
54976	'ADISSP'	mRNA			
54998	'AURKAIP1'	mRNA			
55052	'MRPL20'	mRNA			
55103	'RALGPS2'	mRNA			
552900	'BOLA2'	mRNA	HALLMARK_DNA_REPAIR	H	HALLMARK_DNA_REPAIR
55299	'BRIX1'	mRNA			
55366	'LGR4'	mRNA			
55450	'CAMK2N1'	mRNA			
55454	'CSGALNACT2'	mRNA			
55506	'MACROH2A2'	mRNA	HALLMARK_KRAS_SIGNALING_DN	H	HALLMARK_KRAS_SIGNALING_DN
55651	'NHP2'	mRNA	HALLMARK_MYC_TARGETS_V1+++HALLMARK_UNFOLDED_PROTEIN_RESPONSE	H	HALLMARK_MYC_TARGETS_V1+++HALLMARK_UNFOLDED_PROTEIN_RESPONSE
55701	'ARHGEF40'	mRNA			
55847	'CISD1'	mRNA			
55920	'RCC2'	mRNA			
56106	'PCDHGA10'	mRNA			
56603	'CYP26B1'	mRNA	HALLMARK_ESTROGEN_RESPONSE_EARLY+++HALLMARK_ESTROGEN_RESPONSE_LATE	H	HALLMARK_ESTROGEN_RESPONSE_EARLY+++HALLMARK_ESTROGEN_RESPONSE_LATE
56670	'SUCNR1'	mRNA			
56917	'MEIS3'	mRNA			
56937	'PMEPA1'	mRNA	HALLMARK_ANDROGEN_RESPONSE+++HALLMARK_EPITHELIAL_MESENCHYMAL_TRANSITION+++HALLMARK_TGF_BETA_SIGNALING+++HALLMARK_TNFA_SIGNALING_VIA_NFKB	H	HALLMARK_ANDROGEN_RESPONSE+++HALLMARK_EPITHELIAL_MESENCHYMAL_TRANSITION+++HALLMARK_TGF_BETA_SIGNALING+++HALLMARK_TNFA_SIGNALING_VIA_NFKB
56967	'C14orf132'	mRNA			
56987	'BBX'	mRNA			
56993	'TOMM22'	mRNA	HALLMARK_OXIDATIVE_PHOSPHORYLATION	H	HALLMARK_OXIDATIVE_PHOSPHORYLATION
56999	'ADAMTS9'	mRNA			
571	'BACH1'	mRNA	HALLMARK_HEME_METABOLISM	H	HALLMARK_HEME_METABOLISM
57185	'NIPAL3'	mRNA			
57480	'PLEKHG1'	mRNA			
57488	'ESYT2'	mRNA			
57493	'HEG1'	mRNA			
57515	'SERINC1'	mRNA	HALLMARK_FATTY_ACID_METABOLISM	H	HALLMARK_FATTY_ACID_METABOLISM
57555	'NLGN2'	mRNA	HALLMARK_APICAL_JUNCTION	H	HALLMARK_APICAL_JUNCTION
57561	'ARRDC3'	mRNA			
5757	'PTMA'	mRNA			
57619	'SHROOM3'	mRNA			
5794	'PTPRH'	mRNA			
5796	'PTPRK'	mRNA			
5802	'PTPRS'	mRNA			
58472	'SQOR'	mRNA	HALLMARK_ADIPOGENESIS	H	HALLMARK_ADIPOGENESIS
58476	'TP53INP2'	mRNA	HALLMARK_FATTY_ACID_METABOLISM	H	HALLMARK_FATTY_ACID_METABOLISM
5886	'RAD23A'	mRNA	HALLMARK_HEME_METABOLISM	H	HALLMARK_HEME_METABOLISM
5901	'RAN'	mRNA	HALLMARK_E2F_TARGETS+++HALLMARK_MYC_TARGETS_V1	H	HALLMARK_E2F_TARGETS+++HALLMARK_MYC_TARGETS_V1
5902	'RANBP1'	mRNA	HALLMARK_E2F_TARGETS+++HALLMARK_MYC_TARGETS_V1	H	HALLMARK_E2F_TARGETS+++HALLMARK_MYC_TARGETS_V1
5905	'RANGAP1'	mRNA			
5954	'RCN1'	mRNA			
5999	'RGS4'	mRNA	HALLMARK_EPITHELIAL_MESENCHYMAL_TRANSITION+++HALLMARK_UV_RESPONSE_DN	H	HALLMARK_EPITHELIAL_MESENCHYMAL_TRANSITION+++HALLMARK_UV_RESPONSE_DN
60386	'SLC25A19'	mRNA	HALLMARK_PEROXISOME	H	HALLMARK_PEROXISOME
60489	'APOBEC3G'	mRNA	HALLMARK_COMPLEMENT	H	HALLMARK_COMPLEMENT
60495	'HPSE2'	mRNA			
6059	'ABCE1'	mRNA	HALLMARK_ALLOGRAFT_REJECTION+++HALLMARK_MYC_TARGETS_V1	H	HALLMARK_ALLOGRAFT_REJECTION+++HALLMARK_MYC_TARGETS_V1
6124	'RPL4'	mRNA			
6128	'RPL6'	mRNA	HALLMARK_MYC_TARGETS_V1	H	HALLMARK_MYC_TARGETS_V1
6132	'RPL8'	mRNA			
6134	'RPL10'	mRNA			
6139	'RPL17'	mRNA			
6141	'RPL18'	mRNA	HALLMARK_MYC_TARGETS_V1+++HALLMARK_P53_PATHWAY	H	HALLMARK_MYC_TARGETS_V1+++HALLMARK_P53_PATHWAY
6142	'RPL18A'	mRNA			
6144	'RPL21'	mRNA			
6146	'RPL22'	mRNA	HALLMARK_MYC_TARGETS_V1	H	HALLMARK_MYC_TARGETS_V1
6147	'RPL23A'	mRNA			
6150	'MRPL23'	mRNA	HALLMARK_MYC_TARGETS_V1+++HALLMARK_UV_RESPONSE_UP	H	HALLMARK_MYC_TARGETS_V1+++HALLMARK_UV_RESPONSE_UP
6152	'RPL24'	mRNA			
6154	'RPL26'	mRNA			
6158	'RPL28'	mRNA			
6160	'RPL31'	mRNA			
6161	'RPL32'	mRNA			
6169	'RPL38'	mRNA			
6175	'RPLP0'	mRNA	HALLMARK_MYC_TARGETS_V1	H	HALLMARK_MYC_TARGETS_V1
6176	'RPLP1'	mRNA			
6181	'RPLP2'	mRNA			
6182	'MRPL12'	mRNA			
6185	'RPN2'	mRNA			
6187	'RPS2'	mRNA	HALLMARK_MYC_TARGETS_V1	H	HALLMARK_MYC_TARGETS_V1
6188	'RPS3'	mRNA	HALLMARK_MYC_TARGETS_V1	H	HALLMARK_MYC_TARGETS_V1
6193	'RPS5'	mRNA	HALLMARK_MYC_TARGETS_V1	H	HALLMARK_MYC_TARGETS_V1
6196	'RPS6KA2'	mRNA	HALLMARK_ESTROGEN_RESPONSE_EARLY+++HALLMARK_ESTROGEN_RESPONSE_LATE	H	HALLMARK_ESTROGEN_RESPONSE_EARLY+++HALLMARK_ESTROGEN_RESPONSE_LATE
6203	'RPS9'	mRNA	HALLMARK_ALLOGRAFT_REJECTION	H	HALLMARK_ALLOGRAFT_REJECTION
6205	'RPS11'	mRNA			
6206	'RPS12'	mRNA	HALLMARK_P53_PATHWAY	H	HALLMARK_P53_PATHWAY
6207	'RPS13'	mRNA			
6210	'RPS15A'	mRNA			
6217	'RPS16'	mRNA			
6232	'RPS27'	mRNA			
6234	'RPS28'	mRNA			
6238	'RRBP1'	mRNA			
6273	'S100A2'	mRNA			
6288	'SAA1'	mRNA			
63027	'SLC22A23'	mRNA			
63035	'BCORL1'	mRNA			
6319	'SCD'	mRNA	HALLMARK_ANDROGEN_RESPONSE+++HALLMARK_CHOLESTEROL_HOMEOSTASIS+++HALLMARK_MTORC1_SIGNALING+++HALLMARK_MYOGENESIS	H	HALLMARK_ANDROGEN_RESPONSE+++HALLMARK_CHOLESTEROL_HOMEOSTASIS+++HALLMARK_MTORC1_SIGNALING+++HALLMARK_MYOGENESIS
6345	'SRL'	mRNA			
6372	'CXCL6'	mRNA	HALLMARK_ANGIOGENESIS+++HALLMARK_EPITHELIAL_MESENCHYMAL_TRANSITION+++HALLMARK_INFLAMMATORY_RESPONSE+++HALLMARK_TNFA_SIGNALING_VIA_NFKB	H	HALLMARK_ANGIOGENESIS+++HALLMARK_EPITHELIAL_MESENCHYMAL_TRANSITION+++HALLMARK_INFLAMMATORY_RESPONSE+++HALLMARK_TNFA_SIGNALING_VIA_NFKB
6374	'CXCL5'	mRNA			
63874	'ABHD4'	mRNA	HALLMARK_P53_PATHWAY	H	HALLMARK_P53_PATHWAY
6390	'SDHB'	mRNA	HALLMARK_ADIPOGENESIS+++HALLMARK_OXIDATIVE_PHOSPHORYLATION	H	HALLMARK_ADIPOGENESIS+++HALLMARK_OXIDATIVE_PHOSPHORYLATION
640	'BLK'	mRNA			
64097	'EPB41L4A'	mRNA			
64118	'DUS1L'	mRNA			
64123	'ADGRL4'	mRNA	HALLMARK_KRAS_SIGNALING_UP	H	HALLMARK_KRAS_SIGNALING_UP
6415	'SELENOW'	mRNA	HALLMARK_UV_RESPONSE_UP	H	HALLMARK_UV_RESPONSE_UP
6418	'SET'	mRNA	HALLMARK_MYC_TARGETS_V1	H	HALLMARK_MYC_TARGETS_V1
642	'BLMH'	mRNA			
6432	'SRSF7'	mRNA	HALLMARK_MYC_TARGETS_V1	H	HALLMARK_MYC_TARGETS_V1
64359	'NXN'	mRNA			
6446	'SGK1'	mRNA	HALLMARK_ANDROGEN_RESPONSE+++HALLMARK_ESTROGEN_RESPONSE_LATE+++HALLMARK_KRAS_SIGNALING_DN+++HALLMARK_TNFA_SIGNALING_VIA_NFKB	H	HALLMARK_ANDROGEN_RESPONSE+++HALLMARK_ESTROGEN_RESPONSE_LATE+++HALLMARK_KRAS_SIGNALING_DN+++HALLMARK_TNFA_SIGNALING_VIA_NFKB
647024	'C6orf132'	mRNA			
64764	'CREB3L2'	mRNA			
64805	'P2RY12'	mRNA			
64866	'CDCP1'	mRNA	HALLMARK_IL2_STAT5_SIGNALING	H	HALLMARK_IL2_STAT5_SIGNALING
64943	'NT5DC2'	mRNA			
64975	'MRPL41'	mRNA			
65003	'MRPL11'	mRNA	HALLMARK_OXIDATIVE_PHOSPHORYLATION	H	HALLMARK_OXIDATIVE_PHOSPHORYLATION
65083	'NOL6'	mRNA			
6515	'SLC2A3'	mRNA	HALLMARK_HYPOXIA+++HALLMARK_IL2_STAT5_SIGNALING+++HALLMARK_MTORC1_SIGNALING+++HALLMARK_TNFA_SIGNALING_VIA_NFKB	H	HALLMARK_HYPOXIA+++HALLMARK_IL2_STAT5_SIGNALING+++HALLMARK_MTORC1_SIGNALING+++HALLMARK_TNFA_SIGNALING_VIA_NFKB
6535	'SLC6A8'	mRNA	HALLMARK_EPITHELIAL_MESENCHYMAL_TRANSITION+++HALLMARK_HEME_METABOLISM+++HALLMARK_MYOGENESIS+++HALLMARK_UV_RESPONSE_UP	H	HALLMARK_EPITHELIAL_MESENCHYMAL_TRANSITION+++HALLMARK_HEME_METABOLISM+++HALLMARK_MYOGENESIS+++HALLMARK_UV_RESPONSE_UP
6542	'SLC7A2'	mRNA	HALLMARK_ESTROGEN_RESPONSE_EARLY+++HALLMARK_INFLAMMATORY_RESPONSE	H	HALLMARK_ESTROGEN_RESPONSE_EARLY+++HALLMARK_INFLAMMATORY_RESPONSE
6590	'SLPI'	mRNA	HALLMARK_KRAS_SIGNALING_UP	H	HALLMARK_KRAS_SIGNALING_UP
65993	'MRPS34'	mRNA			
6611	'SMS'	mRNA	HALLMARK_ANDROGEN_RESPONSE+++HALLMARK_FATTY_ACID_METABOLISM	H	HALLMARK_ANDROGEN_RESPONSE+++HALLMARK_FATTY_ACID_METABOLISM
6632	'SNRPD1'	mRNA	HALLMARK_G2M_CHECKPOINT+++HALLMARK_MYC_TARGETS_V1	H	HALLMARK_G2M_CHECKPOINT+++HALLMARK_MYC_TARGETS_V1
6635	'SNRPE'	mRNA			
6637	'SNRPG'	mRNA	HALLMARK_MYC_TARGETS_V1	H	HALLMARK_MYC_TARGETS_V1
664	'BNIP3'	mRNA	HALLMARK_PROTEIN_SECRETION	H	HALLMARK_PROTEIN_SECRETION
6647	'SOD1'	mRNA	HALLMARK_ADIPOGENESIS+++HALLMARK_APOPTOSIS+++HALLMARK_BILE_ACID_METABOLISM+++HALLMARK_GLYCOLYSIS+++HALLMARK_PEROXISOME+++HALLMARK_PROTEIN_SECRETION+++HALLMARK_REACTIVE_OXYGEN_SPECIES_PATHWAY	H	HALLMARK_ADIPOGENESIS+++HALLMARK_APOPTOSIS+++HALLMARK_BILE_ACID_METABOLISM+++HALLMARK_GLYCOLYSIS+++HALLMARK_PEROXISOME+++HALLMARK_PROTEIN_SECRETION+++HALLMARK_REACTIVE_OXYGEN_SPECIES_PATHWAY
6648	'SOD2'	mRNA	HALLMARK_APOPTOSIS+++HALLMARK_INTERFERON_GAMMA_RESPONSE+++HALLMARK_PEROXISOME+++HALLMARK_REACTIVE_OXYGEN_SPECIES_PATHWAY+++HALLMARK_TNFA_SIGNALING_VIA_NFKB+++HALLMARK_UV_RESPONSE_UP	H	HALLMARK_APOPTOSIS+++HALLMARK_INTERFERON_GAMMA_RESPONSE+++HALLMARK_PEROXISOME+++HALLMARK_REACTIVE_OXYGEN_SPECIES_PATHWAY+++HALLMARK_TNFA_SIGNALING_VIA_NFKB+++HALLMARK_UV_RESPONSE_UP
6692	'SPINT1'	mRNA			
6712	'SPTBN2'	mRNA	HALLMARK_KRAS_SIGNALING_DN	H	HALLMARK_KRAS_SIGNALING_DN
6723	'SRM'	mRNA	HALLMARK_MYC_TARGETS_V1+++HALLMARK_MYC_TARGETS_V2	H	HALLMARK_MYC_TARGETS_V1+++HALLMARK_MYC_TARGETS_V2
6727	'SRP14'	mRNA	HALLMARK_PANCREAS_BETA_CELLS	H	HALLMARK_PANCREAS_BETA_CELLS
6744	'ITPRID2'	mRNA			
6748	'SSR4'	mRNA			
6779	'STATH'	mRNA			
6781	'STC1'	mRNA	HALLMARK_ANGIOGENESIS+++HALLMARK_GLYCOLYSIS+++HALLMARK_HYPOXIA+++HALLMARK_MTORC1_SIGNALING	H	HALLMARK_ANGIOGENESIS+++HALLMARK_GLYCOLYSIS+++HALLMARK_HYPOXIA+++HALLMARK_MTORC1_SIGNALING
6876	'TAGLN'	mRNA	HALLMARK_EPITHELIAL_MESENCHYMAL_TRANSITION+++HALLMARK_MYOGENESIS	H	HALLMARK_EPITHELIAL_MESENCHYMAL_TRANSITION+++HALLMARK_MYOGENESIS
6880	'TAF9'	mRNA	HALLMARK_DNA_REPAIR	H	HALLMARK_DNA_REPAIR
6881	'TAF10'	mRNA	HALLMARK_DNA_REPAIR	H	HALLMARK_DNA_REPAIR
689	'BTF3'	mRNA			
6892	'TAPBP'	mRNA	HALLMARK_ALLOGRAFT_REJECTION+++HALLMARK_INFLAMMATORY_RESPONSE+++HALLMARK_INTERFERON_GAMMA_RESPONSE	H	HALLMARK_ALLOGRAFT_REJECTION+++HALLMARK_INFLAMMATORY_RESPONSE+++HALLMARK_INTERFERON_GAMMA_RESPONSE
6950	'TCP1'	mRNA	HALLMARK_MYC_TARGETS_V1	H	HALLMARK_MYC_TARGETS_V1
7045	'TGFBI'	mRNA	HALLMARK_APICAL_JUNCTION+++HALLMARK_EPITHELIAL_MESENCHYMAL_TRANSITION+++HALLMARK_GLYCOLYSIS+++HALLMARK_HYPOXIA	H	HALLMARK_APICAL_JUNCTION+++HALLMARK_EPITHELIAL_MESENCHYMAL_TRANSITION+++HALLMARK_GLYCOLYSIS+++HALLMARK_HYPOXIA
7053	'TGM3'	mRNA			
7056	'THBD'	mRNA	HALLMARK_ANGIOGENESIS+++HALLMARK_COAGULATION	H	HALLMARK_ANGIOGENESIS+++HALLMARK_COAGULATION
7057	'THBS1'	mRNA	HALLMARK_COAGULATION+++HALLMARK_EPITHELIAL_MESENCHYMAL_TRANSITION+++HALLMARK_TGF_BETA_SIGNALING	H	HALLMARK_COAGULATION+++HALLMARK_EPITHELIAL_MESENCHYMAL_TRANSITION+++HALLMARK_TGF_BETA_SIGNALING
7075	'TIE1'	mRNA			
7077	'TIMP2'	mRNA	HALLMARK_APOPTOSIS+++HALLMARK_COMPLEMENT	H	HALLMARK_APOPTOSIS+++HALLMARK_COMPLEMENT
708	'C1QBP'	mRNA	HALLMARK_MYC_TARGETS_V1	H	HALLMARK_MYC_TARGETS_V1
7084	'TK2'	mRNA	HALLMARK_DNA_REPAIR	H	HALLMARK_DNA_REPAIR
7091	'TLE4'	mRNA	HALLMARK_SPERMATOGENESIS	H	HALLMARK_SPERMATOGENESIS
7097	'TLR2'	mRNA	HALLMARK_ALLOGRAFT_REJECTION+++HALLMARK_IL6_JAK_STAT3_SIGNALING+++HALLMARK_INFLAMMATORY_RESPONSE+++HALLMARK_TNFA_SIGNALING_VIA_NFKB	H	HALLMARK_ALLOGRAFT_REJECTION+++HALLMARK_IL6_JAK_STAT3_SIGNALING+++HALLMARK_INFLAMMATORY_RESPONSE+++HALLMARK_TNFA_SIGNALING_VIA_NFKB
71	'ACTG1'	mRNA	HALLMARK_APICAL_JUNCTION+++HALLMARK_CHOLESTEROL_HOMEOSTASIS	H	HALLMARK_APICAL_JUNCTION+++HALLMARK_CHOLESTEROL_HOMEOSTASIS
7127	'TNFAIP2'	mRNA	HALLMARK_INTERFERON_GAMMA_RESPONSE+++HALLMARK_TNFA_SIGNALING_VIA_NFKB	H	HALLMARK_INTERFERON_GAMMA_RESPONSE+++HALLMARK_TNFA_SIGNALING_VIA_NFKB
7128	'TNFAIP3'	mRNA	HALLMARK_COMPLEMENT+++HALLMARK_EPITHELIAL_MESENCHYMAL_TRANSITION+++HALLMARK_HYPOXIA+++HALLMARK_INTERFERON_GAMMA_RESPONSE+++HALLMARK_KRAS_SIGNALING_UP+++HALLMARK_TNFA_SIGNALING_VIA_NFKB	H	HALLMARK_COMPLEMENT+++HALLMARK_EPITHELIAL_MESENCHYMAL_TRANSITION+++HALLMARK_HYPOXIA+++HALLMARK_INTERFERON_GAMMA_RESPONSE+++HALLMARK_KRAS_SIGNALING_UP+++HALLMARK_TNFA_SIGNALING_VIA_NFKB
7138	'TNNT1'	mRNA	HALLMARK_MYOGENESIS	H	HALLMARK_MYOGENESIS
7145	'TNS1'	mRNA	HALLMARK_HEME_METABOLISM	H	HALLMARK_HEME_METABOLISM
718	'C3'	mRNA	HALLMARK_ADIPOGENESIS+++HALLMARK_COAGULATION+++HALLMARK_COMPLEMENT+++HALLMARK_HEME_METABOLISM	H	HALLMARK_ADIPOGENESIS+++HALLMARK_COAGULATION+++HALLMARK_COMPLEMENT+++HALLMARK_HEME_METABOLISM
7203	'CCT3'	mRNA	HALLMARK_MYC_TARGETS_V1	H	HALLMARK_MYC_TARGETS_V1
7223	'TRPC4'	mRNA			
728489	'DNLZ'	mRNA			
7296	'TXNRD1'	mRNA	HALLMARK_MTORC1_SIGNALING+++HALLMARK_REACTIVE_OXYGEN_SPECIES_PATHWAY	H	HALLMARK_MTORC1_SIGNALING+++HALLMARK_REACTIVE_OXYGEN_SPECIES_PATHWAY
7314	'UBB'	mRNA			
7332	'UBE2L3'	mRNA	HALLMARK_MYC_TARGETS_V1	H	HALLMARK_MYC_TARGETS_V1
7347	'UCHL3'	mRNA			
7388	'UQCRH'	mRNA	HALLMARK_OXIDATIVE_PHOSPHORYLATION	H	HALLMARK_OXIDATIVE_PHOSPHORYLATION
7417	'VDAC2'	mRNA	HALLMARK_APOPTOSIS+++HALLMARK_OXIDATIVE_PHOSPHORYLATION	H	HALLMARK_APOPTOSIS+++HALLMARK_OXIDATIVE_PHOSPHORYLATION
7431	'VIM'	mRNA	HALLMARK_EPITHELIAL_MESENCHYMAL_TRANSITION	H	HALLMARK_EPITHELIAL_MESENCHYMAL_TRANSITION
7436	'VLDLR'	mRNA	HALLMARK_GLYCOLYSIS+++HALLMARK_HEDGEHOG_SIGNALING+++HALLMARK_HYPOXIA+++HALLMARK_MTORC1_SIGNALING+++HALLMARK_UV_RESPONSE_DN	H	HALLMARK_GLYCOLYSIS+++HALLMARK_HEDGEHOG_SIGNALING+++HALLMARK_HYPOXIA+++HALLMARK_MTORC1_SIGNALING+++HALLMARK_UV_RESPONSE_DN
7498	'XDH'	mRNA	HALLMARK_XENOBIOTIC_METABOLISM	H	HALLMARK_XENOBIOTIC_METABOLISM
7529	'YWHAB'	mRNA	HALLMARK_PI3K_AKT_MTOR_SIGNALING	H	HALLMARK_PI3K_AKT_MTOR_SIGNALING
767	'CA8'	mRNA			
7706	'TRIM25'	mRNA	HALLMARK_INTERFERON_ALPHA_RESPONSE+++HALLMARK_INTERFERON_GAMMA_RESPONSE	H	HALLMARK_INTERFERON_ALPHA_RESPONSE+++HALLMARK_INTERFERON_GAMMA_RESPONSE
79050	'NOC4L'	mRNA	HALLMARK_MYC_TARGETS_V2	H	HALLMARK_MYC_TARGETS_V2
79056	'PRRG4'	mRNA			
79077	'DCTPP1'	mRNA	HALLMARK_E2F_TARGETS+++HALLMARK_MYC_TARGETS_V2	H	HALLMARK_E2F_TARGETS+++HALLMARK_MYC_TARGETS_V2
79080	'CCDC86'	mRNA			
79144	'PPDPF'	mRNA			
7919	'DDX39B'	mRNA			
79365	'BHLHE41'	mRNA			
79627	'OGFRL1'	mRNA			
7965	'AIMP2'	mRNA	HALLMARK_MYC_TARGETS_V1+++HALLMARK_MYC_TARGETS_V2	H	HALLMARK_MYC_TARGETS_V1+++HALLMARK_MYC_TARGETS_V2
79693	'YRDC'	mRNA	HALLMARK_KRAS_SIGNALING_UP+++HALLMARK_TNFA_SIGNALING_VIA_NFKB	H	HALLMARK_KRAS_SIGNALING_UP+++HALLMARK_TNFA_SIGNALING_VIA_NFKB
79709	'COLGALT1'	mRNA	HALLMARK_EPITHELIAL_MESENCHYMAL_TRANSITION	H	HALLMARK_EPITHELIAL_MESENCHYMAL_TRANSITION
79772	'MCTP1'	mRNA			
79803	'HPS6'	mRNA			
79875	'THSD4'	mRNA	HALLMARK_ESTROGEN_RESPONSE_EARLY	H	HALLMARK_ESTROGEN_RESPONSE_EARLY
79901	'CYBRD1'	mRNA			
79924	'ADM2'	mRNA			
79971	'WLS'	mRNA	HALLMARK_IL2_STAT5_SIGNALING	H	HALLMARK_IL2_STAT5_SIGNALING
80142	'PTGES2'	mRNA			
80177	'MYCT1'	mRNA			
8029	'CUBN'	mRNA			
80833	'APOL3'	mRNA			
8091	'HMGA2'	mRNA			
81029	'WNT5B'	mRNA	HALLMARK_WNT_BETA_CATENIN_SIGNALING	H	HALLMARK_WNT_BETA_CATENIN_SIGNALING
81031	'SLC2A10'	mRNA			
811	'CALR'	mRNA	HALLMARK_MTORC1_SIGNALING+++HALLMARK_PI3K_AKT_MTOR_SIGNALING+++HALLMARK_UNFOLDED_PROTEIN_RESPONSE	H	HALLMARK_MTORC1_SIGNALING+++HALLMARK_PI3K_AKT_MTOR_SIGNALING+++HALLMARK_UNFOLDED_PROTEIN_RESPONSE
813	'CALU'	mRNA	HALLMARK_EPITHELIAL_MESENCHYMAL_TRANSITION	H	HALLMARK_EPITHELIAL_MESENCHYMAL_TRANSITION
8148	'TAF15'	mRNA			
81563	'C1orf21'	mRNA			
81605	'URM1'	mRNA			
81631	'MAP1LC3B'	mRNA			
8260	'NAA10'	mRNA			
8270	'LAGE3'	mRNA			
8337	'H2AC18'	mRNA			
834	'CASP1'	mRNA	HALLMARK_APOPTOSIS+++HALLMARK_COMPLEMENT+++HALLMARK_INTERFERON_ALPHA_RESPONSE+++HALLMARK_INTERFERON_GAMMA_RESPONSE+++HALLMARK_P53_PATHWAY	H	HALLMARK_APOPTOSIS+++HALLMARK_COMPLEMENT+++HALLMARK_INTERFERON_ALPHA_RESPONSE+++HALLMARK_INTERFERON_GAMMA_RESPONSE+++HALLMARK_P53_PATHWAY
83442	'SH3BGRL3'	mRNA			
83461	'CDCA3'	mRNA	HALLMARK_E2F_TARGETS	H	HALLMARK_E2F_TARGETS
8349	'H2BC21'	mRNA			
83648	'FAM167A'	mRNA			
83743	'GRWD1'	mRNA	HALLMARK_MYC_TARGETS_V2	H	HALLMARK_MYC_TARGETS_V2
83787	'ARMC10'	mRNA			
83879	'CDCA7'	mRNA			
8407	'TAGLN2'	mRNA			
8409	'UXT'	mRNA	HALLMARK_MITOTIC_SPINDLE	H	HALLMARK_MITOTIC_SPINDLE

84300	'UQCC2'	mRNA			
84306	'PDCD2L'	mRNA	HALLMARK_IL2_STAT5_SIGNALING	H	HALLMARK_IL2_STAT5_SIGNALING
84552	'PARD6G'	mRNA	HALLMARK_APICAL_JUNCTION	H	HALLMARK_APICAL_JUNCTION
84632	'AFAP1L2'	mRNA	HALLMARK_APICAL_SURFACE	H	HALLMARK_APICAL_SURFACE
84650	'EBPL'	mRNA			
84790	'TUBA1C'	mRNA			
84844	'PHF5A'	mRNA	HALLMARK_E2F_TARGETS	H	HALLMARK_E2F_TARGETS
84916	'UTP4'	mRNA			
84969	'TOX2'	mRNA			
84985	'FAM83A'	mRNA			
8514	'KCNAB2'	mRNA			
8519	'IFITM1'	mRNA	HALLMARK_INFLAMMATORY_RESPONSE+++HALLMARK_INTERFERON_ALPHA_RESPONSE	H	HALLMARK_INFLAMMATORY_RESPONSE+++HALLMARK_INTERFERON_ALPHA_RESPONSE
85236	'H2BC12'	mRNA	HALLMARK_G2M_CHECKPOINT	H	HALLMARK_G2M_CHECKPOINT
85363	'TRIM5'	mRNA	HALLMARK_INTERFERON_ALPHA_RESPONSE	H	HALLMARK_INTERFERON_ALPHA_RESPONSE
8542	'APOL1'	mRNA			
85441	'HELZ2'	mRNA	HALLMARK_INTERFERON_ALPHA_RESPONSE+++HALLMARK_INTERFERON_GAMMA_RESPONSE	H	HALLMARK_INTERFERON_ALPHA_RESPONSE+++HALLMARK_INTERFERON_GAMMA_RESPONSE
8568	'RRP1'	mRNA			
8626	'TP63'	mRNA	HALLMARK_P53_PATHWAY	H	HALLMARK_P53_PATHWAY
8631	'SKAP1'	mRNA			
8638	'OASL'	mRNA	HALLMARK_INTERFERON_ALPHA_RESPONSE+++HALLMARK_INTERFERON_GAMMA_RESPONSE	H	HALLMARK_INTERFERON_ALPHA_RESPONSE+++HALLMARK_INTERFERON_GAMMA_RESPONSE
8704	'B4GALT2'	mRNA	HALLMARK_GLYCOLYSIS	H	HALLMARK_GLYCOLYSIS
871	'SERPINH1'	mRNA	HALLMARK_EPITHELIAL_MESENCHYMAL_TRANSITION+++HALLMARK_MTORC1_SIGNALING	H	HALLMARK_EPITHELIAL_MESENCHYMAL_TRANSITION+++HALLMARK_MTORC1_SIGNALING
8767	'RIPK2'	mRNA	HALLMARK_ALLOGRAFT_REJECTION+++HALLMARK_INFLAMMATORY_RESPONSE+++HALLMARK_INTERFERON_ALPHA_RESPONSE+++HALLMARK_INTERFERON_GAMMA_RESPONSE+++HALLMARK_TNFA_SIGNALING_VIA_NFKB	H	HALLMARK_ALLOGRAFT_REJECTION+++HALLMARK_INFLAMMATORY_RESPONSE+++HALLMARK_INTERFERON_ALPHA_RESPONSE+++HALLMARK_INTERFERON_GAMMA_RESPONSE+++HALLMARK_TNFA_SIGNALING_VIA_NFKB
8811	'GALR2'	mRNA			
8877	'SPHK1'	mRNA	HALLMARK_INFLAMMATORY_RESPONSE+++HALLMARK_MYOGENESIS+++HALLMARK_P53_PATHWAY+++HALLMARK_TNFA_SIGNALING_VIA_NFKB	H	HALLMARK_INFLAMMATORY_RESPONSE+++HALLMARK_MYOGENESIS+++HALLMARK_P53_PATHWAY+++HALLMARK_TNFA_SIGNALING_VIA_NFKB
8878	'SQSTM1'	mRNA	HALLMARK_APOPTOSIS+++HALLMARK_MTORC1_SIGNALING+++HALLMARK_PI3K_AKT_MTOR_SIGNALING+++HALLMARK_TNFA_SIGNALING_VIA_NFKB+++HALLMARK_UV_RESPONSE_UP	H	HALLMARK_APOPTOSIS+++HALLMARK_MTORC1_SIGNALING+++HALLMARK_PI3K_AKT_MTOR_SIGNALING+++HALLMARK_TNFA_SIGNALING_VIA_NFKB+++HALLMARK_UV_RESPONSE_UP
8892	'EIF2B2'	mRNA			
890	'CCNA2'	mRNA	HALLMARK_G2M_CHECKPOINT+++HALLMARK_MYC_TARGETS_V1	H	HALLMARK_G2M_CHECKPOINT+++HALLMARK_MYC_TARGETS_V1
896	'CCND3'	mRNA	HALLMARK_ALLOGRAFT_REJECTION+++HALLMARK_ANDROGEN_RESPONSE+++HALLMARK_HEME_METABOLISM+++HALLMARK_IL2_STAT5_SIGNALING+++HALLMARK_P53_PATHWAY+++HALLMARK_UV_RESPONSE_UP	H	HALLMARK_ALLOGRAFT_REJECTION+++HALLMARK_ANDROGEN_RESPONSE+++HALLMARK_HEME_METABOLISM+++HALLMARK_IL2_STAT5_SIGNALING+++HALLMARK_P53_PATHWAY+++HALLMARK_UV_RESPONSE_UP
89797	'NAV2'	mRNA	HALLMARK_ESTROGEN_RESPONSE_EARLY+++HALLMARK_MYOGENESIS	H	HALLMARK_ESTROGEN_RESPONSE_EARLY+++HALLMARK_MYOGENESIS
89891	'DYNC2I2'	mRNA			
8995	'TNFSF18'	mRNA			
89958	'SAPCD2'	mRNA			
90139	'TSPAN18'	mRNA			
9045	'RPL14'	mRNA	HALLMARK_MYC_TARGETS_V1	H	HALLMARK_MYC_TARGETS_V1
9060	'PAPSS2'	mRNA	HALLMARK_ESTROGEN_RESPONSE_EARLY+++HALLMARK_ESTROGEN_RESPONSE_LATE+++HALLMARK_XENOBIOTIC_METABOLISM	H	HALLMARK_ESTROGEN_RESPONSE_EARLY+++HALLMARK_ESTROGEN_RESPONSE_LATE+++HALLMARK_XENOBIOTIC_METABOLISM
9076	'CLDN1'	mRNA			
908	'CCT6A'	mRNA	HALLMARK_MTORC1_SIGNALING	H	HALLMARK_MTORC1_SIGNALING
91663	'MYADM'	mRNA			
91754	'NEK9'	mRNA			
9180	'OSMR'	mRNA	HALLMARK_IL6_JAK_STAT3_SIGNALING+++HALLMARK_INFLAMMATORY_RESPONSE	H	HALLMARK_IL6_JAK_STAT3_SIGNALING+++HALLMARK_INFLAMMATORY_RESPONSE
9188	'DDX21'	mRNA	HALLMARK_MYC_TARGETS_V1+++HALLMARK_UV_RESPONSE_UP	H	HALLMARK_MYC_TARGETS_V1+++HALLMARK_UV_RESPONSE_UP
9208	'LRRFIP1'	mRNA			
9212	'AURKB'	mRNA	HALLMARK_E2F_TARGETS+++HALLMARK_G2M_CHECKPOINT	H	HALLMARK_E2F_TARGETS+++HALLMARK_G2M_CHECKPOINT
92126	'DSEL'	mRNA			
9221	'NOLC1'	mRNA	HALLMARK_E2F_TARGETS+++HALLMARK_G2M_CHECKPOINT+++HALLMARK_MYC_TARGETS_V1+++HALLMARK_MYC_TARGETS_V2+++HALLMARK_UNFOLDED_PROTEIN_RESPONSE	H	HALLMARK_E2F_TARGETS+++HALLMARK_G2M_CHECKPOINT+++HALLMARK_MYC_TARGETS_V1+++HALLMARK_MYC_TARGETS_V2+++HALLMARK_UNFOLDED_PROTEIN_RESPONSE
9235	'IL32'	mRNA	HALLMARK_EPITHELIAL_MESENCHYMAL_TRANSITION	H	HALLMARK_EPITHELIAL_MESENCHYMAL_TRANSITION
9246	'UBE2L6'	mRNA	HALLMARK_FATTY_ACID_METABOLISM+++HALLMARK_INTERFERON_ALPHA_RESPONSE+++HALLMARK_INTERFERON_GAMMA_RESPONSE	H	HALLMARK_FATTY_ACID_METABOLISM+++HALLMARK_INTERFERON_ALPHA_RESPONSE+++HALLMARK_INTERFERON_GAMMA_RESPONSE
92689	'FAM114A1'	mRNA			
928	'CD9'	mRNA	HALLMARK_CHOLESTEROL_HOMEOSTASIS+++HALLMARK_COAGULATION+++HALLMARK_ESTROGEN_RESPONSE_LATE+++HALLMARK_IL6_JAK_STAT3_SIGNALING+++HALLMARK_MTORC1_SIGNALING	H	HALLMARK_CHOLESTEROL_HOMEOSTASIS+++HALLMARK_COAGULATION+++HALLMARK_ESTROGEN_RESPONSE_LATE+++HALLMARK_IL6_JAK_STAT3_SIGNALING+++HALLMARK_MTORC1_SIGNALING
93099	'DMKN'	mRNA			
9349	'RPL23'	mRNA			
9377	'COX5A'	mRNA	HALLMARK_MYC_TARGETS_V1+++HALLMARK_OXIDATIVE_PHOSPHORYLATION	H	HALLMARK_MYC_TARGETS_V1+++HALLMARK_OXIDATIVE_PHOSPHORYLATION
9378	'NRXN1'	mRNA			
9404	'LPXN'	mRNA			
9411	'ARHGAP29'	mRNA	HALLMARK_MITOTIC_SPINDLE	H	HALLMARK_MITOTIC_SPINDLE
9423	'NTN1'	mRNA			
94239	'H2AZ2'	mRNA	HALLMARK_G2M_CHECKPOINT	H	HALLMARK_G2M_CHECKPOINT
9500	'MAGED1'	mRNA			
9516	'LITAF'	mRNA	HALLMARK_TNFA_SIGNALING_VIA_NFKB	H	HALLMARK_TNFA_SIGNALING_VIA_NFKB
9538	'EI24'	mRNA	HALLMARK_P53_PATHWAY	H	HALLMARK_P53_PATHWAY
9563	'H6PD'	mRNA			
9572	'NR1D1'	mRNA			
9582	'APOBEC3B'	mRNA			
9588	'PRDX6'	mRNA	HALLMARK_FATTY_ACID_METABOLISM+++HALLMARK_REACTIVE_OXYGEN_SPECIES_PATHWAY	H	HALLMARK_FATTY_ACID_METABOLISM+++HALLMARK_REACTIVE_OXYGEN_SPECIES_PATHWAY
9590	'AKAP12'	mRNA	HALLMARK_ANDROGEN_RESPONSE+++HALLMARK_HYPOXIA+++HALLMARK_KRAS_SIGNALING_UP	H	HALLMARK_ANDROGEN_RESPONSE+++HALLMARK_HYPOXIA+++HALLMARK_KRAS_SIGNALING_UP
9619	'ABCG1'	mRNA			
9636	'ISG15'	mRNA	HALLMARK_INTERFERON_ALPHA_RESPONSE+++HALLMARK_INTERFERON_GAMMA_RESPONSE	H	HALLMARK_INTERFERON_ALPHA_RESPONSE+++HALLMARK_INTERFERON_GAMMA_RESPONSE
9638	'FEZ1'	mRNA	HALLMARK_APOPTOSIS	H	HALLMARK_APOPTOSIS
9644	'SH3PXD2A'	mRNA			
9647	'PPM1F'	mRNA			
9648	'GCC2'	mRNA			
9659	'PDE4DIP'	mRNA	HALLMARK_MYOGENESIS	H	HALLMARK_MYOGENESIS
9734	'HDAC9'	mRNA	HALLMARK_ALLOGRAFT_REJECTION+++HALLMARK_KRAS_SIGNALING_UP	H	HALLMARK_ALLOGRAFT_REJECTION+++HALLMARK_KRAS_SIGNALING_UP
9751	'SNPH'	mRNA			
9788	'MTSS1'	mRNA			
9804	'TOMM20'	mRNA			
9839	'ZEB2'	mRNA			
9871	'SEC24D'	mRNA	HALLMARK_ANDROGEN_RESPONSE+++HALLMARK_PROTEIN_SECRETION	H	HALLMARK_ANDROGEN_RESPONSE+++HALLMARK_PROTEIN_SECRETION
9877	'ZC3H11A'	mRNA			
9902	'MRC2'	mRNA			
991	'CDC20'	mRNA	HALLMARK_E2F_TARGETS+++HALLMARK_ESTROGEN_RESPONSE_LATE+++HALLMARK_G2M_CHECKPOINT+++HALLMARK_MYC_TARGETS_V1	H	HALLMARK_E2F_TARGETS+++HALLMARK_ESTROGEN_RESPONSE_LATE+++HALLMARK_G2M_CHECKPOINT+++HALLMARK_MYC_TARGETS_V1
9933	'PUM3'	mRNA			
9961	'MVP'	mRNA	HALLMARK_INTERFERON_GAMMA_RESPONSE+++HALLMARK_PEROXISOME	H	HALLMARK_INTERFERON_GAMMA_RESPONSE+++HALLMARK_PEROXISOME
