# Supplementary material for: Angiotensin II type 1 receptor signaling promotes bladder cancer progression and its inhibition by Losartan
Source: Hypertens Res. 2026 Jan 19;49(4):1480–94. doi: 10.1038/s41440-025-02535-y (PMC13050642; doi:10.1038/s41440-025-02535-y)
Supplement: Supplementary file 5 — Table S4 [file 41440_2025_2535_MOESM5_ESM.pdf]

Table S5: Top 20 pathways most significantly altered in AGTR1-overexpressing versus control T24 bladder cancer cells.

MSigDB H(hallmark) Term ID	MSigDB H(hallmark) Term Desc	MSigDB H(hallmark) Level1	MSigDB H(hallmark) Level2	Term Candidate Gene Num	Total Candidate Gene Num	Term Gene Num	Total Gene Num	Rich Ratio	P value	Q value	Gene ids	Gene Symbols
HALLMARK_MYC_TARGETS_V1	A subgroup of genes regulated by MYC - version 1 (v1).	H	HALLMARK_MYC_TARGETS_V1	56	400	200	4378	0.28	2.16E-15	1.08E-13	10155,1019,10528,10576,10935,10971,1973,2091,22948,26986,3178,3251,3326,3329,3336,38<br>38,4176,4609,4830,4869,4953,5036,5111,51690,5245,5250,54107,5425,55651,5901,5902,6059<br>,6128,6141,6146,6150,6175,6187,6188,6193,6418,6432,6632,6637,6723,6950,708,7203,7332,7,	TRIM28,CDK4,NOP56,CCT2,PRDX3,YWHAQ,EIF4A1,FBL,CCT5,PABPC1,HNRNPA1,HPRT1,HSP90AB1,HSPD1,HSP<br>E1,KPNA2,MCM7,MYC,NME1,NPM1,ODC1,PA2G4,PCNA,LSM7,PHB1,SLC25A3,POLE3,POLD2,NHP2,RAN,RANBP1<br>ABCE1,RPL6,RPL18,RPL22,MRPL23,RPLP0,RPS2,RPS3,RPS5,SET,SRSF7,SNRPD1,SNRPG,SRM,TCP1,C1QBP,CC
HALLMARK_MYC_TARGETS_V2	A subgroup of genes regulated by MYC - version 2 (v2).	H	HALLMARK_MYC_TARGETS_V2	21	400	58	4378	0.362068966	1.25E-08	3.12E-07	##	CDK4,RABEPK,MYBBP1A,NOP56,TMEM97,HSPD1,HSPE1,MYC,NOP2,NPM1,PA2G4,MRTO4,PHB1,PLK1,WDR74,S
HALLMARK_TNFA_SIGNALING_VIA_NFKB	Genes regulated by NF-kB in response to TNF [GeneID=7124].	H	HALLMARK_TNFA_SIGNALING_VIA_NFKB	43	400	200	4378	0.215	4.29E-08	7.16E-07	10135,1026,10318,1051,1435,1437,182,1839,1846,19,2150,23258,24147,2669,2919,2920,330,<br>3383,3433,3491,3552,3553,3604,3606,3914,4609,4792,490,50486,5055,56937,6372,6446,6515	NAMPT,CDKN1A,TNIP1,CEBPB,CSF1,CSF2,JAG1,HBEGF,DUSP4,ABCA1,F2RL1,DENND5A,FJX1,GEM,CXCL1,CXCL<br>2,BIRC3,ICAM1,IFIT2,CCN1,IL1A,IL1B,TNFRSF9,IL18,LAMB3,MYC,NFKBIA,ATP2B1,G0S2,SERPINB2,PMEPA1,CXC
HALLMARK_EPITHELIAL_MESENCHYMAL_TRANSITION	Genes defining epithelial-mesenchymal transition, as in wound healing, fibrosis and metastasis.	H	HALLMARK_EPITHELIAL_MESENCHYMAL_TRANSITION	38	400	200	4378	0.19	6.95E-06	8.69E-05	##	MYL9,COL4A1,COL4A2,COL12A1,CCN2,ENO2,FOXC2,ABI3BP,GEM,GJA1,CXCL1,CCN1,ITGB3,LAMC2,LOX,LRP1,M<br>CM7,MMP1,MYLK,NNMT,NT5E,TNFRSF11B,TNFRSF12A,SERPINE2,PLOD2,PMEPA1,RGS4,CXCL6,SLC6A8,TAGL
HALLMARK_MTORC1_SIGNALING	Genes up-regulated through activation of mTORC1 complex.	H	HALLMARK_MTORC1_SIGNALING	34	400	200	4378	0.17	2.22E-04	0.002224946	##	NAMPT,CDKN1A,TOMM40,CTSC,EGLN3,NIBAN1,AK4,SLC7A11,PHGDH,CACYBP,TMEM97,HMGCS1,HPRT1,HSPA<br>9,HSPD1,HSPE1,IGFBP5,PRDX1,RDH11,ATP5MC1,PDK1,PLK1,PLOD2,DDIT4,SCD,SLC2A3,STC1,TXNRD1,VLDLR,
HALLMARK_E2F_TARGETS	Genes encoding cell cycle related targets of E2F transcription factors.	H	HALLMARK_E2F_TARGETS	30	400	200	4378	0.15	0.00401618	0.03346817	##	CDK4,CDKN1A,NOP56,PAICS,CKS1B,CSE1L,TUBB,AK2,UBE2S,H2AX,HMGB3,KPNA2,LMNB1,MCM3,MCM7,MYBL<br>2,MYC,NME1,PA2G4,PCNA,PLK1,POLD2,RAN,RANBP1,DCTPP1,CDCA3,PHF5A,AURKB,NOLC1,CDC20
HALLMARK_OXIDATIVE_PHOSPHORYLATION	Genes encoding proteins involved in oxidative phosphorylation.	H	HALLMARK_OXIDATIVE_PHOSPHORYLATION	29	400	200	4378	0.145	0.007539142	0.05385101	##	PRDX3,COX7A2,COX7B,COX7C,COX8A,CPT1A,TIMM13,TIMM10,TIMM8B,GPX4,SLC25A5,HSPA9,LDHB,NDUFA1,N<br>DUFS3,NDUFS6,NDUFS8,ATP5F1B,ATP5F1D,ATP5F1E,ATP5MC1,ATP5MC3,SLC25A3,TOMM22,SDHB,MRPL11,U
HALLMARK_INFLAMMATORY_RESPONSE	Genes defining inflammatory response.	H	HALLMARK_INFLAMMATORY_RESPONSE	28	400	200	4378	0.14	0.01361794	0.08463189	10135,1026,10630,1435,1839,19,1902,3091,3383,3552,3553,3604,3606,3656,3690,3732,391,4<br>609,4792,490,6372,6542,6892,7097,8519,8767,8877,9180	NAMPT,CDKN1A,PDPN,CSF1,HBEGF,ABCA1,LPAR1,HIF1A,ICAM1,IL1A,IL1B,TNFRSF9,IL18,IRAK2,ITGB3,CD82,RH<br>OG,MYC,NFKBIA,ATP2B1,CXCL6,SLC7A2,TAPBP,TLR2,IFITM1,RIPK2,SPHK1,OSMR
HALLMARK_CHOLESTEROL_HOMEOSTASIS	Genes involved in cholesterol homeostasis.	H	HALLMARK_CHOLESTEROL_HOMEOSTASIS	13	400	74	4378	0.175675676	0.01523374	0.08463189	116496,182,20,2171,2194,27346,3157,51330,54206,5468,6319,71,928	NIBAN1,JAG1,ABCA2,FABP5,FASN,TMEM97,HMGCS1,TNFRSF12A,ERRFI1,PPARG,SCD,ACTG1,CD9
HALLMARK_UV_RESPONSE_DN	Genes down-regulated in response to ultraviolet (UV) radiation.	H	HALLMARK_UV_RESPONSE_DN	21	400	144	4378	0.145833333	0.02010536	0.1005268	##	CITED2,DLC1,MGLL,LPAR1,EFEMP1,FYN,GJA1,ANXA2,IGFBP5,CCN1,ITGB3,MAP1B,MMP16,MT1E,MYC,ATP2B1,
HALLMARK_G2M_CHECKPOINT	Genes involved in the G2/M checkpoint, as in progression through the cell division cycle.	H	HALLMARK_G2M_CHECKPOINT	27	400	199	4378	0.135678392	0.02230165	0.1013711	##	CDK4,UBE2C,FOXN3,CKS1B,DKC1,UBE2S,H2AX,HIF1A,HMGB3,HMGN2,HSPA8,KPNA2,LMNB1,MCM3,MYBL2,MY<br>C,ODC1,GINS2,MAP3K20,PLK1,SNRPD1,H2BC12,CCNA2,AURKB,NOLC1,H2AZ2,CDC20
HALLMARK_UNFOLDED_PROTEIN_RESPONSE	Genes up-regulated during unfolded protein response, a cellular stress response related to the endoplasmic reticulum.	H	HALLMARK_UNFOLDED_PROTEIN_RESPONSE	17	400	113	4378	0.150442478	0.0263049	0.1096037	##	CEBPB,NOP56,KDELR3,CKS1B,DDX10,DKC1,EIF4A1,EIF4EBP1,H2AX,HSPA9,DNAJB9,NPM1,GEMIN4,DDIT4,NHP2
HALLMARK_INTERFERON_ALPHA_RESPONSE	Genes up-regulated in response to alpha interferon proteins.	H	HALLMARK_INTERFERON_ALPHA_RESPONSE	15	400	97	4378	0.154639175	0.02858844	0.1099555	1,435,263,434,293,430,000,000,000,000,000,000,000,000,000,000,000,000,000,000,000	CSF1,GBP2,IFI27,IFIT2,ISG20,MX1,TRIM25,CASP1,IFITM1,TRIM5,HELZ2,OASL,RIPK2,UBE2L6,ISG15
HALLMARK_ANDROGEN_RESPONSE	Genes defining response to androgens.	H	HALLMARK_ANDROGEN_RESPONSE	15	400	100	4378	0.15	0.03641524	0.1300544	10,397,162,227,347,300,000,000,000,000,000,000,000,000,000,000,000,000,000,000,000	NDRG1,DBI,STK39,H1-0,HMGCS1,KRT8,DNAJB9,PA2G4,PMEPA1,SCD,SGK1,SMS,CCND3,AKAP12,SEC24D
HALLMARK_HYPOXIA	Genes up-regulated in response to low oxygen levels (hypoxia).	H	HALLMARK_HYPOXIA	26	400	200	4378	0.13	0.03950536	0.1316845	##	CDKN1A,CITED2,NDRG1,KDELR3,B3GALT6,CCN2,ENO2,AK4,NEDD4L,ANXA2,HOXB9,CCN1,ISG20,LOX,MT1E,PA<br>M,PDK1,ERRFI1,DDIT4,KLHL24,SLC2A3,STC1,TGFBI,TNFAIP3,VLDLR,AKAP12
HALLMARK_APOPTOSIS	Genes mediating programmed cell death (apoptosis) by activation of caspases.	H	HALLMARK_APOPTOSIS	21	400	161	4378	0.130434783	0.05846592	0.182706	##	CDKN1A,ENO2,PDCD4,GPX1,GPX4,H1-
HALLMARK_INTERFERON_GAMMA_RESPONSE	Genes up-regulated in response to IFNG [GeneID=3458].	H	HALLMARK_INTERFERON_GAMMA_RESPONSE	25	400	200	4378	0.125	0.06339071	0.1864433	##	NAMPT,CDKN1A,HIF1A,HLA-<br>B,ICAM1,IFI27,IFIT2,IFNAR2,ISG20,MX1,NFKBIA,OAS2,XAF1,SOD2,TAPBP,TNFAIP2,TNFAIP3,TRIM25,CASP1,HEL
HALLMARK_FATTY_ACID_METABOLISM	Genes encoding proteins involved in metabolism of fatty acids.	H	HALLMARK_FATTY_ACID_METABOLISM	19	400	157	4378	0.121019108	0.1225265	0.3371782	##	HSPH1,MGLL,CPT1A,ADSL,ENO2,FASN,GABARAPL1,IL4I1,HMGCS1,HSP90AA1,ODC1,G0S2,PCBD1,RDH11,SERIN
HALLMARK_UV_RESPONSE_UP	Genes up-regulated in response to ultraviolet (UV) radiation.	H	HALLMARK_UV_RESPONSE_UP	19	400	158	4378	0.120253165	0.1281277	0.3371782	##	DLG4,ENO2,FKBP4,NPTXR,GLS,CXCL2,H2AX,DNAJA1,DNAJB1,ICAM1,NFKBIA,POLE3,MRPL23,SELENOW,SLC6A8
HALLMARK_REACTIVE_OXYGEN_SPECIES_PATHWAY	Genes up-regulated by reactive oxigen species (ROS).	H	HALLMARK_REACTIVE_OXYGEN_SPECIES_PATHWAY	7	400	49	4378	0.142857143	0.1553067	0.3882667	2,879,316,350,526,640,000,000,000,000	GPX4,HMOX2,PRDX1,SOD1,SOD2,TXNRD1,PRDX6
