# Supplementary material for: Angiotensin II type 1 receptor signaling promotes bladder cancer progression and its inhibition by Losartan
Source: Hypertens Res. 2026 Jan 19;49(4):1480–94. doi: 10.1038/s41440-025-02535-y (PMC13050642; doi:10.1038/s41440-025-02535-y)
Supplement: Supplementary file 6 — Supplementary Figure 1 [file 41440_2025_2535_MOESM6_ESM.pptx]

## Slide 1
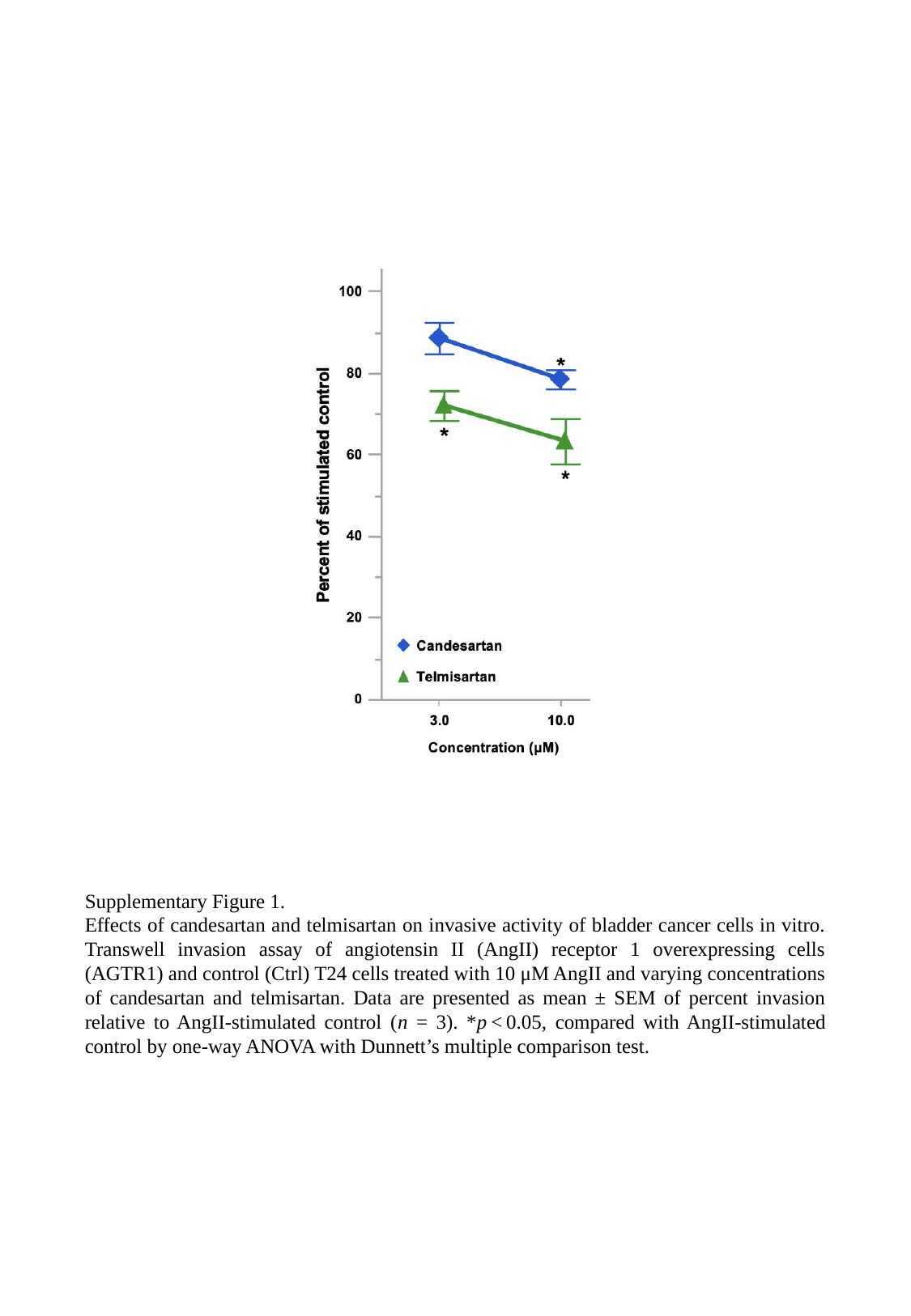

Supplementary Figure 1.
Effects of candesartan and telmisartan on invasive activity of bladder cancer cells in vitro. Transwell invasion assay of angiotensin II (AngII) receptor 1 overexpressing cells (AGTR1) and control (Ctrl) T24 cells treated with 10 μM AngII and varying concentrations of candesartan and telmisartan. Data are presented as mean ± SEM of percent invasion relative to AngII-stimulated control (n = 3). *p < 0.05, compared with AngII-stimulated control by one-way ANOVA with Dunnett’s multiple comparison test.
