# Supplementary material for: Angiotensin II type 1 receptor signaling promotes bladder cancer progression and its inhibition by Losartan
Source: Hypertens Res. 2026 Jan 19;49(4):1480–94. doi: 10.1038/s41440-025-02535-y (PMC13050642; doi:10.1038/s41440-025-02535-y)
Supplement: Supplementary file 7 — Supplementary Figure 2 [file 41440_2025_2535_MOESM7_ESM.pptx]

## Slide 1
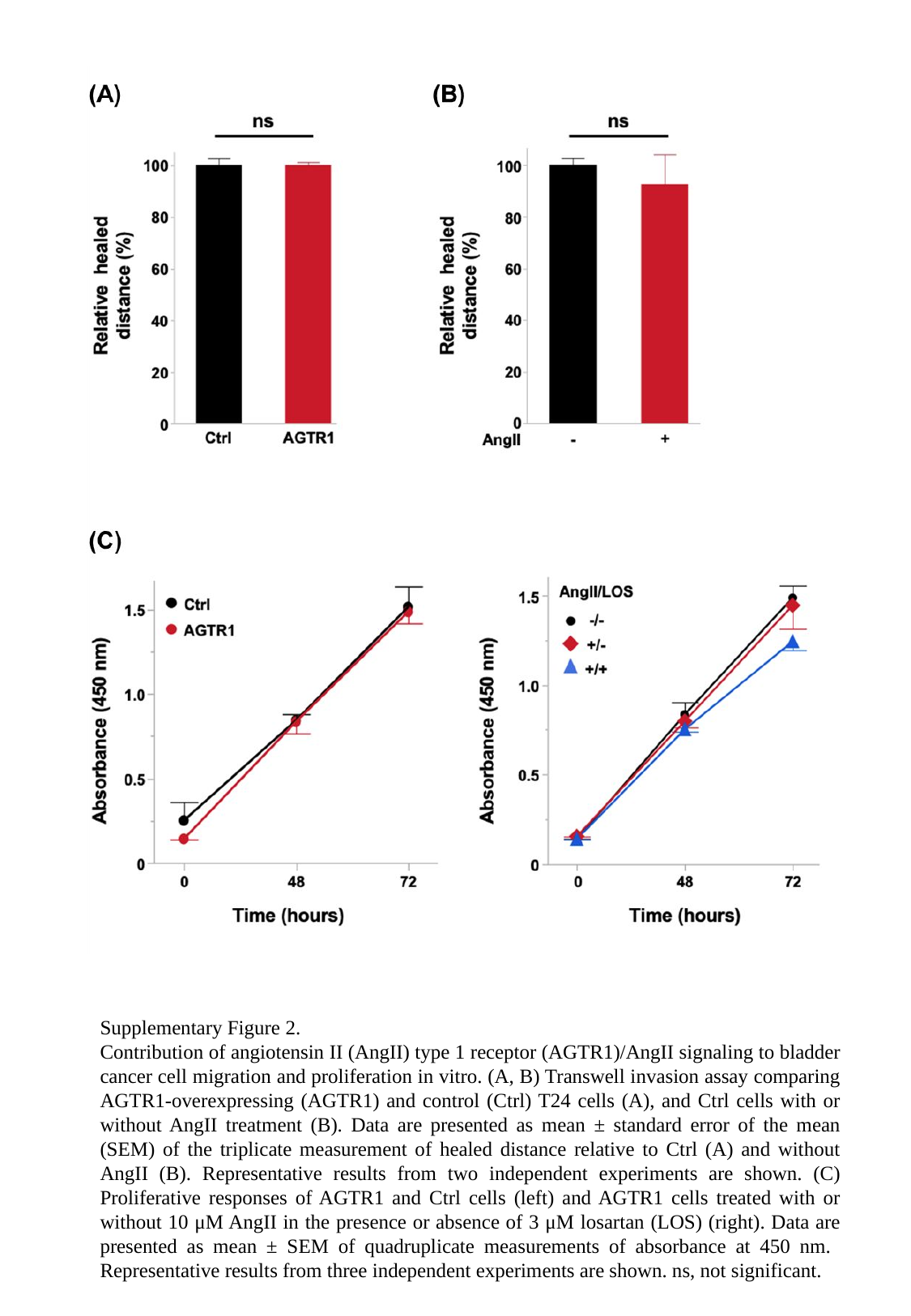

Supplementary Figure 2.
Contribution of angiotensin II (AngII) type 1 receptor (AGTR1)/AngII signaling to bladder cancer cell migration and proliferation in vitro. (A, B) Transwell invasion assay comparing AGTR1-overexpressing (AGTR1) and control (Ctrl) T24 cells (A), and Ctrl cells with or without AngII treatment (B). Data are presented as mean ± standard error of the mean (SEM) of the triplicate measurement of healed distance relative to Ctrl (A) and without AngII (B). Representative results from two independent experiments are shown. (C) Proliferative responses of AGTR1 and Ctrl cells (left) and AGTR1 cells treated with or without 10 μM AngII in the presence or absence of 3 μM losartan (LOS) (right). Data are presented as mean ± SEM of quadruplicate measurements of absorbance at 450 nm. Representative results from three independent experiments are shown. ns, not significant.
