# Supplementary material for: Angiotensin II type 1 receptor signaling promotes bladder cancer progression and its inhibition by Losartan
Source: Hypertens Res. 2026 Jan 19;49(4):1480–94. doi: 10.1038/s41440-025-02535-y (PMC13050642; doi:10.1038/s41440-025-02535-y)
Supplement: Supplementary file 8 — Supplementary Figure 3 [file 41440_2025_2535_MOESM8_ESM.pptx]

## Slide 1
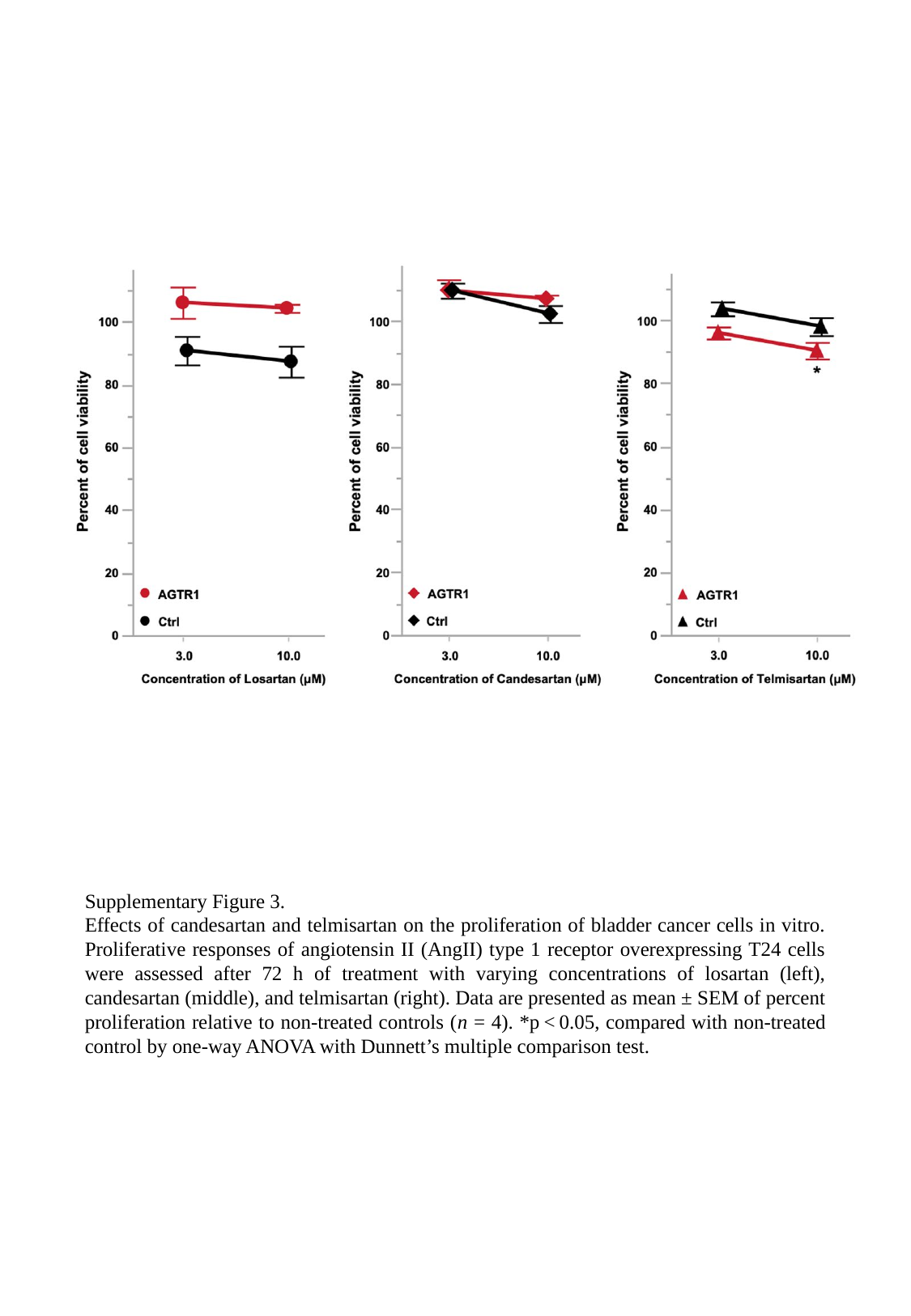

Supplementary Figure 3.
Effects of candesartan and telmisartan on the proliferation of bladder cancer cells in vitro. Proliferative responses of angiotensin II (AngII) type 1 receptor overexpressing T24 cells were assessed after 72 h of treatment with varying concentrations of losartan (left), candesartan (middle), and telmisartan (right). Data are presented as mean ± SEM of percent proliferation relative to non-treated controls (n = 4). *p < 0.05, compared with non-treated control by one-way ANOVA with Dunnett’s multiple comparison test.
