# Supplementary material for: Angiotensin II type 1 receptor signaling promotes bladder cancer progression and its inhibition by Losartan
Source: Hypertens Res. 2026 Jan 19;49(4):1480–94. doi: 10.1038/s41440-025-02535-y (PMC13050642; doi:10.1038/s41440-025-02535-y)
Supplement: Supplementary file 9 — Supplementary Figure 4 [file 41440_2025_2535_MOESM9_ESM.pptx]

## Slide 1
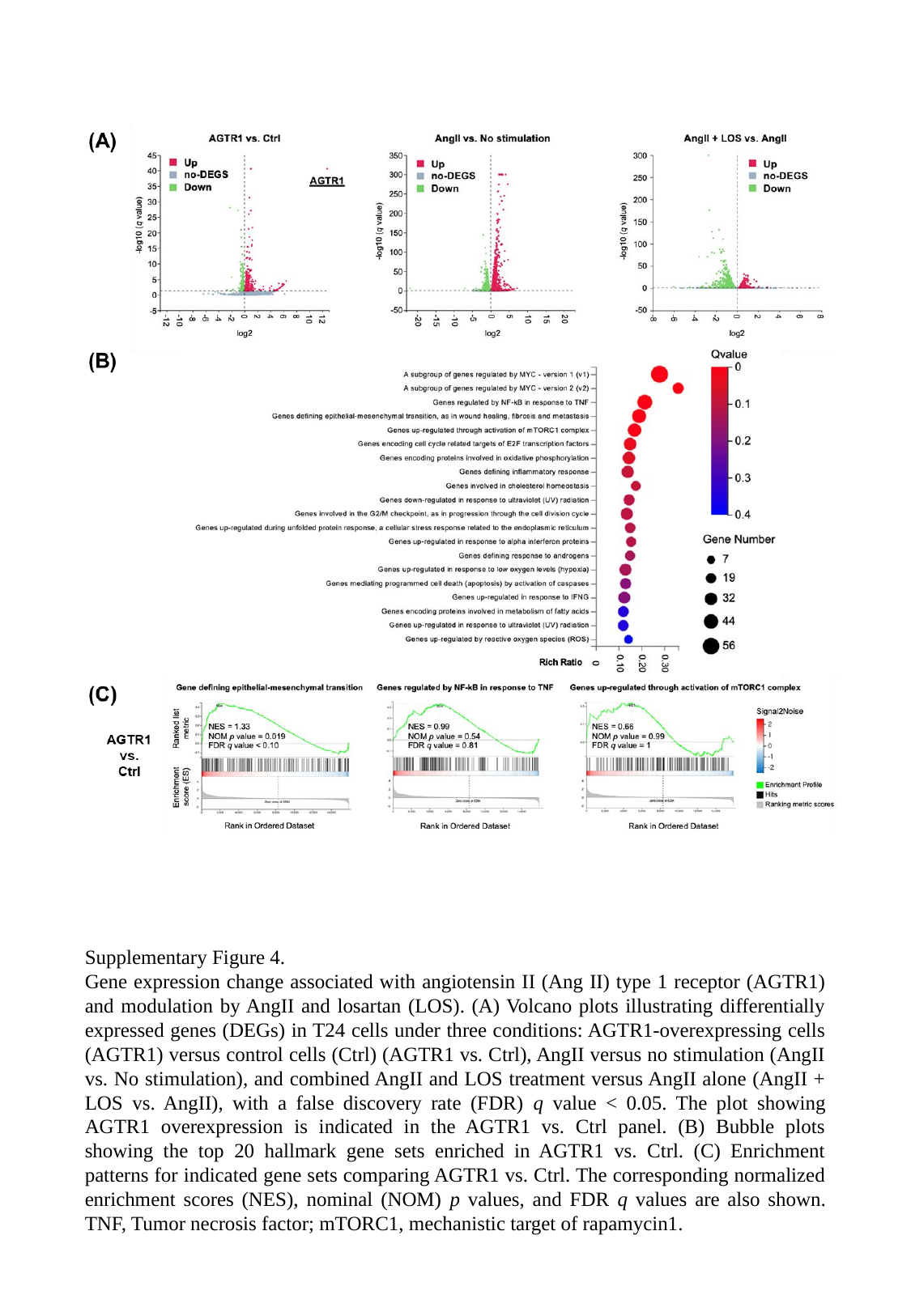

Supplementary Figure 4.
Gene expression change associated with angiotensin II (Ang II) type 1 receptor (AGTR1) and modulation by AngII and losartan (LOS). (A) Volcano plots illustrating differentially expressed genes (DEGs) in T24 cells under three conditions: AGTR1-overexpressing cells (AGTR1) versus control cells (Ctrl) (AGTR1 vs. Ctrl), AngII versus no stimulation (AngII vs. No stimulation), and combined AngII and LOS treatment versus AngII alone (AngII + LOS vs. AngII), with a false discovery rate (FDR) q value < 0.05. The plot showing AGTR1 overexpression is indicated in the AGTR1 vs. Ctrl panel. (B) Bubble plots showing the top 20 hallmark gene sets enriched in AGTR1 vs. Ctrl. (C) Enrichment patterns for indicated gene sets comparing AGTR1 vs. Ctrl. The corresponding normalized enrichment scores (NES), nominal (NOM) p values, and FDR q values are also shown. TNF, Tumor necrosis factor; mTORC1, mechanistic target of rapamycin1.
