# Supplementary material for: Angiotensin II type 1 receptor signaling promotes bladder cancer progression and its inhibition by Losartan
Source: Hypertens Res. 2026 Jan 19;49(4):1480–94. doi: 10.1038/s41440-025-02535-y (PMC13050642; doi:10.1038/s41440-025-02535-y)
Supplement: Supplementary file 10 — Supplementary Figure 5 [file 41440_2025_2535_MOESM10_ESM.pptx]

## Slide 1
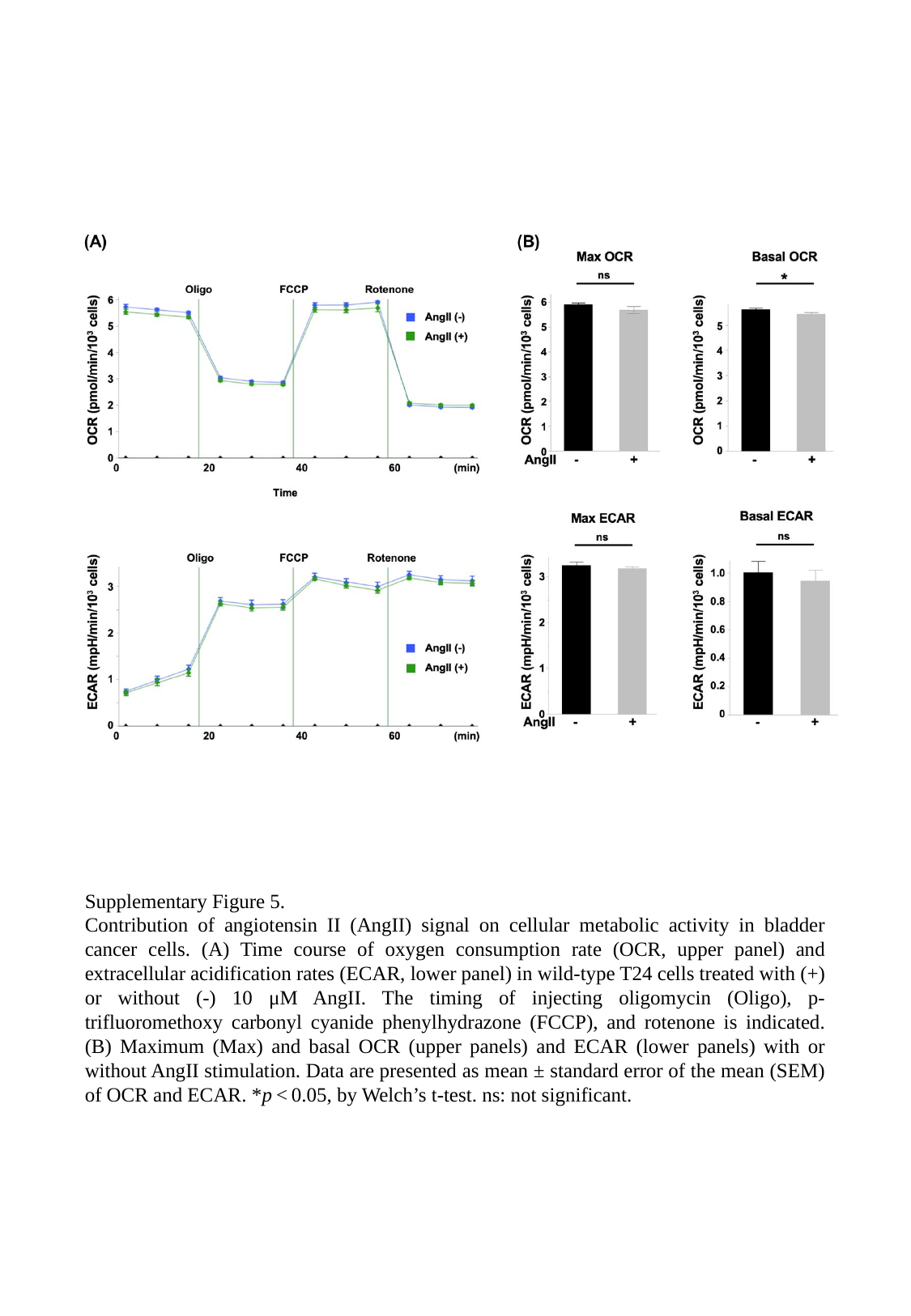

Supplementary Figure 5.
Contribution of angiotensin II (AngII) signal on cellular metabolic activity in bladder cancer cells. (A) Time course of oxygen consumption rate (OCR, upper panel) and extracellular acidification rates (ECAR, lower panel) in wild-type T24 cells treated with (+) or without (-) 10 μM AngII. The timing of injecting oligomycin (Oligo), p-trifluoromethoxy carbonyl cyanide phenylhydrazone (FCCP), and rotenone is indicated. (B) Maximum (Max) and basal OCR (upper panels) and ECAR (lower panels) with or without AngII stimulation. Data are presented as mean ± standard error of the mean (SEM) of OCR and ECAR. *p < 0.05, by Welch’s t-test. ns: not significant.
