# Supplementary material for: Angiotensin II type 1 receptor signaling promotes bladder cancer progression and its inhibition by Losartan
Source: Hypertens Res. 2026 Jan 19;49(4):1480–94. doi: 10.1038/s41440-025-02535-y (PMC13050642; doi:10.1038/s41440-025-02535-y)
Supplement: Supplementary file 11 — Supplementary Figure 6 [file 41440_2025_2535_MOESM11_ESM.pptx]

## Slide 1
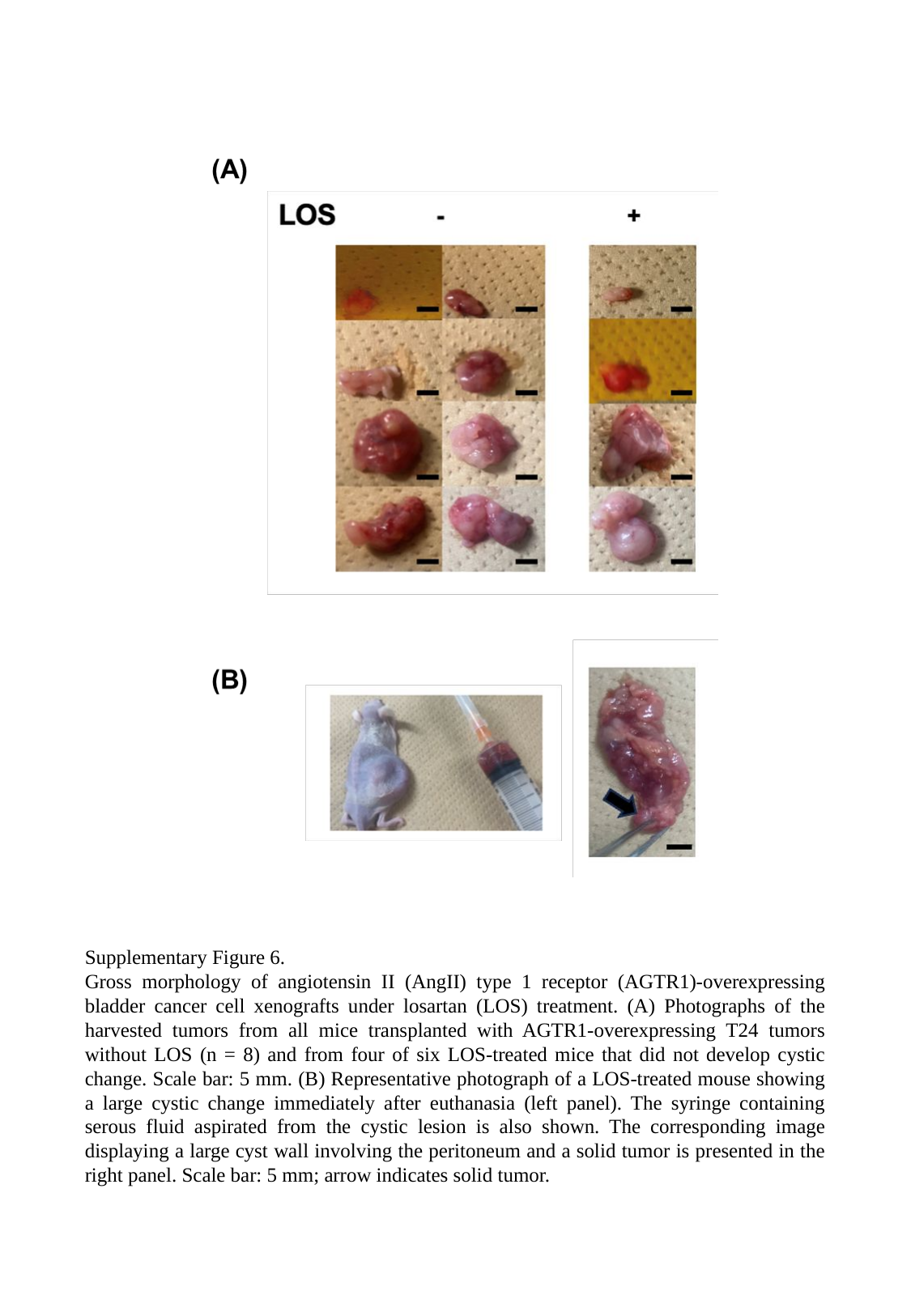

Supplementary Figure 6.
Gross morphology of angiotensin II (AngII) type 1 receptor (AGTR1)-overexpressing bladder cancer cell xenografts under losartan (LOS) treatment. (A) Photographs of the harvested tumors from all mice transplanted with AGTR1-overexpressing T24 tumors without LOS (n = 8) and from four of six LOS-treated mice that did not develop cystic change. Scale bar: 5 mm. (B) Representative photograph of a LOS-treated mouse showing a large cystic change immediately after euthanasia (left panel). The syringe containing serous fluid aspirated from the cystic lesion is also shown. The corresponding image displaying a large cyst wall involving the peritoneum and a solid tumor is presented in the right panel. Scale bar: 5 mm; arrow indicates solid tumor.
