# Supplementary material for: Angiotensin II type 1 receptor signaling promotes bladder cancer progression and its inhibition by Losartan
Source: Hypertens Res. 2026 Jan 19;49(4):1480–94. doi: 10.1038/s41440-025-02535-y (PMC13050642; doi:10.1038/s41440-025-02535-y)
Supplement: Supplementary file 12 — Supplementary Figure 7 [file 41440_2025_2535_MOESM12_ESM.pptx]

## Slide 1
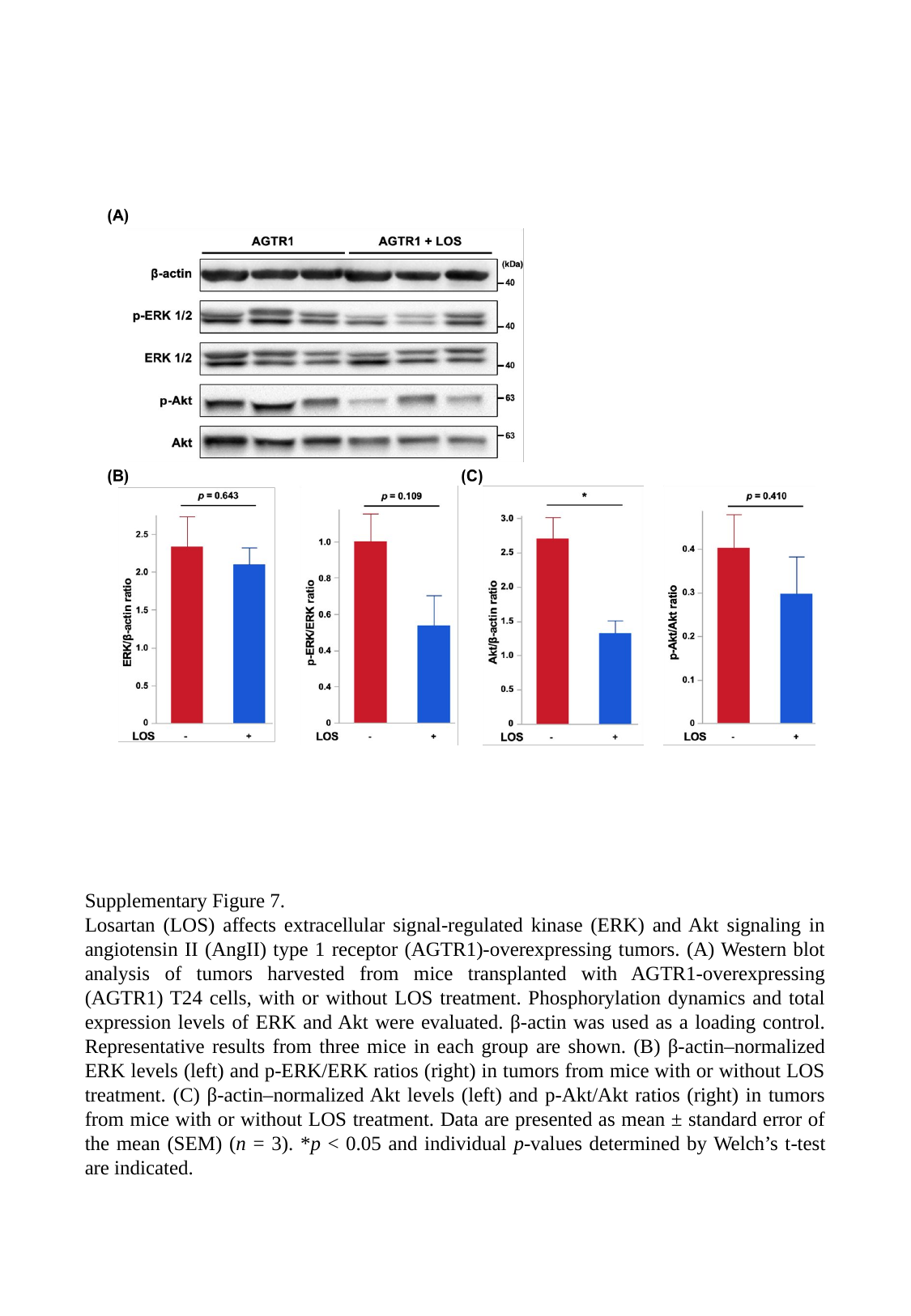

Supplementary Figure 7.
Losartan (LOS) affects extracellular signal-regulated kinase (ERK) and Akt signaling in angiotensin II (AngII) type 1 receptor (AGTR1)-overexpressing tumors. (A) Western blot analysis of tumors harvested from mice transplanted with AGTR1-overexpressing (AGTR1) T24 cells, with or without LOS treatment. Phosphorylation dynamics and total expression levels of ERK and Akt were evaluated. β-actin was used as a loading control. Representative results from three mice in each group are shown. (B) β-actin–normalized ERK levels (left) and p‑ERK/ERK ratios (right) in tumors from mice with or without LOS treatment. (C) β-actin–normalized Akt levels (left) and p‑Akt/Akt ratios (right) in tumors from mice with or without LOS treatment. Data are presented as mean ± standard error of the mean (SEM) (n = 3). *p < 0.05 and individual p-values determined by Welch’s t-test are indicated.
