# Supplementary material for: Angiotensin II type 1 receptor signaling promotes bladder cancer progression and its inhibition by Losartan
Source: Hypertens Res. 2026 Jan 19;49(4):1480–94. doi: 10.1038/s41440-025-02535-y (PMC13050642; doi:10.1038/s41440-025-02535-y)
Supplement: Supplementary file 13 — Supplementary legend [file 41440_2025_2535_MOESM13_ESM.docx]

**Supporting Information**

Supplementary Figure 1.

Effects of candesartan and telmisartan on invasive activity of bladder cancer cells in vitro. Transwell invasion assay of angiotensin II (AngII) receptor 1 overexpressing cells (AGTR1) and control (Ctrl) T24 cells treated with 10 μM AngII and varying concentrations of candesartan and telmisartan. Data are presented as mean ± SEM of percent invasion relative to AngII-stimulated control (*n* = 3). **p*< 0.05, compared with AngII-stimulated control by one-way ANOVA with Dunnett’s multiple comparison test.

Supplementary Figure 2.

Contribution of angiotensin II (AngII) type 1 receptor (AGTR1)/AngII signaling to bladder cancer cell migration and proliferation in vitro. (A, B) Transwell invasion assay comparing AGTR1-overexpressing (AGTR1) and control (Ctrl) T24 cells (A), and Ctrl cells with or without AngII treatment (B). Data are presented as mean ± standard error of the mean (SEM) of the triplicate measurement of healed distance relative to Ctrl (A) and without AngII (B). Representative results from two independent experiments are shown. (C) Proliferative responses of AGTR1 and Ctrl cells (left) and AGTR1 cells treated with or without 10 μM AngII in the presence or absence of 3 μM losartan (LOS) (right). Data are presented as mean ± SEM of quadruplicate measurements of absorbance at 450 nm. Representative results from three independent experiments are shown. ns, not significant.

Supplementary Figure 3.

Effects of candesartan and telmisartan on the proliferation of bladder cancer cells in vitro. Proliferative responses of angiotensin II (AngII) type 1 receptor overexpressing T24 cells were assessed after 72 h of treatment with varying concentrations of losartan (left), candesartan (middle), and telmisartan (right). Data are presented as mean ± SEM of percent proliferation relative to non-treated controls (*n* = 4). **p*< 0.05, compared with non-treated control by one-way ANOVA with Dunnett’s multiple comparison test.

Supplementary Figure 4.

Gene expression change associated with angiotensin II (Ang II) type 1 receptor (AGTR1) and modulation by AngII and losartan (LOS). (A) Volcano plots illustrating differentially expressed genes (DEGs) in T24 cells under three conditions: AGTR1-overexpressing cells (AGTR1) versus control cells (Ctrl) (AGTR1 vs. Ctrl), AngII versus no stimulation (AngII vs. No stimulation), and combined AngII and LOS treatment versus AngII alone (AngII + LOS vs. AngII), with a false discovery rate (FDR) *q* value < 0.05. The plot showing AGTR1 overexpression is indicated in the AGTR1 vs. Ctrl panel. (B) Bubble plots showing the top 20 hallmark gene sets enriched in AGTR1 vs. Ctrl. (C) Enrichment patterns for indicated gene sets comparing AGTR1 vs. Ctrl. The corresponding normalized enrichment scores (NES), nominal (NOM) *p* values, and FDR *q* values are also shown. TNF, Tumor necrosis factor; mTORC1, mechanistic target of rapamycin1.

Supplementary Figure 5.

Contribution of angiotensin II (AngII) signal on cellular metabolic activity in bladder cancer cells. (A) Time course of oxygen consumption rate (OCR, upper panel) and extracellular acidification rates (ECAR, lower panel) in wild-type T24 cells treated with (+) or without (-) 10 μM AngII. The timing of injecting oligomycin (Oligo), p-trifluoromethoxy carbonyl cyanide phenylhydrazone (FCCP), and rotenone is indicated. (B) Maximum (Max) and basal OCR (upper panels) and ECAR (lower panels) with or without AngII stimulation. Data are presented as mean ± standard error of the mean (SEM) of OCR and ECAR. **p*< 0.05, by Welch’s t-test. ns: not significant.

Supplementary Figure 6.

Gross morphology of angiotensin II (AngII) type 1 receptor (AGTR1)-overexpressing bladder cancer cell xenografts under losartan (LOS) treatment. (A) Photographs of the harvested tumors from all mice transplanted with AGTR1-overexpressing T24 tumors without LOS (n = 8) and from four of six LOS-treated mice that did not develop cystic change. Scale bar: 5 mm. (B) Representative photograph of a LOS-treated mouse showing a large cystic change immediately after euthanasia (left panel). The syringe containing serous fluid aspirated from the cystic lesion is also shown. The corresponding image displaying a large cyst wall involving the peritoneum and a solid tumor is presented in the right panel. Scale bar: 5 mm; arrow indicates solid tumor.

Supplementary Figure 7.

Losartan (LOS) affects extracellular signal-regulated kinase (ERK) and Akt signaling in angiotensin II (AngII) type 1 receptor (AGTR1)-overexpressing tumors. (A) Western blot analysis of tumors harvested from mice transplanted with AGTR1-overexpressing (AGTR1) T24 cells, with or without LOS treatment. Phosphorylation dynamics and total expression levels of ERK and Akt were evaluated. β-actin was used as a loading control. Representative results from three mice in each group are shown. (B) β-actin–normalized ERK levels (left) and p‑ERK/ERK ratios (right) in tumors from mice with or without LOS treatment. (C) β-actin–normalized Akt levels (left) and p‑Akt/Akt ratios (right) in tumors from mice with or without LOS treatment. Data are presented as mean ± standard error of the mean (SEM) (*n* = 3). **p* < 0.05 and individual *p*-values determined by Welch’s t-test are indicated.
