# Supplementary material for: Angiotensin II type 1 receptor signaling promotes bladder cancer progression and its inhibition by Losartan
Source: Hypertens Res. 2026 Jan 19;49(4):1480–94. doi: 10.1038/s41440-025-02535-y (PMC13050642; doi:10.1038/s41440-025-02535-y)
Supplement: Supplementary file 2 — Table S1 [file 41440_2025_2535_MOESM2_ESM.pdf]

Table S2. Genes commonly altered across two comparisons: Angiotensin II (AngII) versus no stimulation, and AngII plus losartan versus AngII alone.

Gene ID	Gene Symbol	Type	MSigDB H(hallmark) Term	MSigDB H(hallmark) Level1	MSigDB H(hallmark) Level2
10000	'AKT3'	mRNA	HALLMARK_APICAL_JUNCTION+++HALLMARK_PANCREAS_BETA_CELLS+++HALLMARK_UV_RESPONSE_DN	H	HALLMARK_APICAL_JUNCTION+++HALLMARK_PANCREAS_BETA_CELLS+++HALLMARK_UV_RESPONSE_DN
100101490	'MCTS2'	mRNA			
10014	'HDAC5'	mRNA	HALLMARK_MYOGENESIS+++HALLMARK_WNT_BETA_CATENIN_SIGNALING	H	HALLMARK_MYOGENESIS+++HALLMARK_WNT_BETA_CATENIN_SIGNALING
1003	'CDH5'	mRNA			
100302736	'TMED7-TICAM2'	mRNA			
10036	'CHAF1A'	mRNA	HALLMARK_G2M_CHECKPOINT	H	HALLMARK_G2M_CHECKPOINT
10040	'TOM1L1'	mRNA	HALLMARK_PROTEIN_SECRETION	H	HALLMARK_PROTEIN_SECRETION
10043	'TOM1'	mRNA			
100505573	'INAFM2'	mRNA			
100507436	'MICA'	mRNA			
100529241	'HSPE1-MOB4'	mRNA			
100533496	'TVP23C-CDRT4'	mRNA			
10055	'SAE1'	mRNA			
10056	'FARSB'	mRNA			
10060	'ABCC9'	mRNA			
10073	'SNUPN'	mRNA			
100820829	'MYZAP'	mRNA			
100996928	'FMC1-LUC7L2'	mRNA			
101	'ADAM8'	mRNA	HALLMARK_KRAS_SIGNALING_UP	H	HALLMARK_KRAS_SIGNALING_UP
10113	'PREB'	mRNA	HALLMARK_ADIPOGENESIS+++HALLMARK_UNFOLDED_PROTEIN_RESPONSE	H	HALLMARK_ADIPOGENESIS+++HALLMARK_UNFOLDED_PROTEIN_RESPONSE
1013	'CDH15'	mRNA	HALLMARK_APICAL_JUNCTION	H	HALLMARK_APICAL_JUNCTION
10130	'PDIA6'	mRNA	HALLMARK_UNFOLDED_PROTEIN_RESPONSE	H	HALLMARK_UNFOLDED_PROTEIN_RESPONSE
10133	'OPTN'	mRNA	HALLMARK_BILE_ACID_METABOLISM+++HALLMARK_HEME_METABOLISM	H	HALLMARK_BILE_ACID_METABOLISM+++HALLMARK_HEME_METABOLISM
10135	'NAMPT'	mRNA	HALLMARK_INFLAMMATORY_RESPONSE+++HALLMARK_INTERFERON_GAMMA_RESPONSE+++HALLMARK_MTORC1_SIGNALING+++HALLMARK_TNFA_SIGNALING_VIA_NFKB	H	HALLMARK_INFLAMMATORY_RESPONSE+++HALLMARK_INTERFERON_GAMMA_RESPONSE+++HALLMARK_MTORC1_SIGNALING+++HALLMARK_TNFA_SIGNALING_VIA_NFKB
10137	'RBM12'	mRNA			
10140	'TOB1'	mRNA	HALLMARK_ADIPOGENESIS+++HALLMARK_ESTROGEN_RESPONSE_EARLY+++HALLMARK_ESTROGEN_RESPONSE_LATE+++HALLMARK_P53_PATHWAY	H	HALLMARK_ADIPOGENESIS+++HALLMARK_ESTROGEN_RESPONSE_EARLY+++HALLMARK_ESTROGEN_RESPONSE_LATE+++HALLMARK_P53_PATHWAY
10150	'MBNL2'	mRNA			
10152	'ABI2'	mRNA			
10153	'CEBPZ'	mRNA			
10155	'TRIM28'	mRNA	HALLMARK_MYC_TARGETS_V1	H	HALLMARK_MYC_TARGETS_V1
10193	'RNF41'	mRNA			
10196	'PRMT3'	mRNA	HALLMARK_MYC_TARGETS_V2	H	HALLMARK_MYC_TARGETS_V2
10205	'MPZL2'	mRNA	HALLMARK_APICAL_JUNCTION+++HALLMARK_KRAS_SIGNALING_UP	H	HALLMARK_APICAL_JUNCTION+++HALLMARK_KRAS_SIGNALING_UP
10206	'TRIM13'	mRNA			
10209	'EIF1'	mRNA	HALLMARK_TNFA_SIGNALING_VIA_NFKB	H	HALLMARK_TNFA_SIGNALING_VIA_NFKB
10217	'CTDSPL'	mRNA			
1022	'CDK7'	mRNA	HALLMARK_PEROXISOME	H	HALLMARK_PEROXISOME
10221	'TRIB1'	mRNA	HALLMARK_KRAS_SIGNALING_UP+++HALLMARK_TNFA_SIGNALING_VIA_NFKB	H	HALLMARK_KRAS_SIGNALING_UP+++HALLMARK_TNFA_SIGNALING_VIA_NFKB
10237	'SLC35B1'	mRNA	HALLMARK_XENOBIOTIC_METABOLISM	H	HALLMARK_XENOBIOTIC_METABOLISM
10241	'CALCOCO2'	mRNA			
10243	'GPHN'	mRNA	HALLMARK_ADIPOGENESIS	H	HALLMARK_ADIPOGENESIS
10253	'SPRY2'	mRNA	HALLMARK_KRAS_SIGNALING_UP	H	HALLMARK_KRAS_SIGNALING_UP
1026	'CDKN1A'	mRNA	HALLMARK_APOPTOSIS+++HALLMARK_E2F_TARGETS+++HALLMARK_HYPOXIA+++HALLMARK_INFLAMMATORY_RESPONSE+++HALLMARK_INTERFERON_GAMMA_RESPONSE+++HALLMARK_MTORC1_SIGNALING+++HALLMARK_MYOGENESIS+++HALLMARK_P53_PATHWAY+++HALLMA	H	HALLMARK_APOPTOSIS+++HALLMARK_E2F_TARGETS+++HALLMARK_HYPOXIA+++HALLMARK_INFLAMMATORY_RESPONSE+++HALLMARK_INTERFERON_GAMMA_RESPONSE+++HALLMARK_MTORC1_SIGNALING+++HALLMARK_MYOGENESIS+++HALLMARK_P53_PATHWAY+++HALLMARK_PI3K_AKT_MTOR_SIGNALING+++HALLMARK_TNFA_SIGNALING_VIA_NFKB
10263	'CDK2AP2'	mRNA			
1027	'CDKN1B'	mRNA	HALLMARK_APOPTOSIS+++HALLMARK_E2F_TARGETS+++HALLMARK_G2M_CHECKPOINT+++HALLMARK_HYPOXIA+++HALLMARK_PI3K_AKT_MTOR_SIGNALING+++HALLMARK_UV_RESPONSE_DN	H	HALLMARK_APOPTOSIS+++HALLMARK_E2F_TARGETS+++HALLMARK_G2M_CHECKPOINT+++HALLMARK_HYPOXIA+++HALLMARK_PI3K_AKT_MTOR_SIGNALING+++HALLMARK_UV_RESPONSE_DN
10272	'FSTL3'	mRNA	HALLMARK_EPITHELIAL_MESENCHYMAL_TRANSITION	H	HALLMARK_EPITHELIAL_MESENCHYMAL_TRANSITION
102723996	'LOC102723996'	mRNA			
10280	'SIGMAR1'	mRNA	HALLMARK_UV_RESPONSE_UP	H	HALLMARK_UV_RESPONSE_UP
10282	'BET1'	mRNA	HALLMARK_PROTEIN_SECRETION	H	HALLMARK_PROTEIN_SECRETION
10291	'SF3A1'	mRNA	HALLMARK_MYC_TARGETS_V1	H	HALLMARK_MYC_TARGETS_V1
10309	'CCNO'	mRNA	HALLMARK_DNA_REPAIR	H	HALLMARK_DNA_REPAIR
10311	'VPS26C'	mRNA			
10318	'TNIP1'	mRNA	HALLMARK_TNFA_SIGNALING_VIA_NFKB	H	HALLMARK_TNFA_SIGNALING_VIA_NFKB
10342	'TFG'	mRNA			
10370	'CITED2'	mRNA	HALLMARK_GLYCOLYSIS+++HALLMARK_HYPOXIA+++HALLMARK_UV_RESPONSE_DN	H	HALLMARK_GLYCOLYSIS+++HALLMARK_HYPOXIA+++HALLMARK_UV_RESPONSE_DN
10371	'SEMA3A'	mRNA			
10376	'TUBA1B'	mRNA			
10381	'TUBB3'	mRNA			
10383	'TUBB4B'	mRNA			
10385	'BTN2A2'	mRNA			
1039	'CDR2'	mRNA	HALLMARK_HEME_METABOLISM	H	HALLMARK_HEME_METABOLISM
103910	'MYL12B'	mRNA	HALLMARK_APICAL_JUNCTION	H	HALLMARK_APICAL_JUNCTION
10397	'NDRG1'	mRNA	HALLMARK_ANDROGEN_RESPONSE+++HALLMARK_HYPOXIA+++HALLMARK_IL2_STAT5_SIGNALING+++HALLMARK_P53_PATHWAY	H	HALLMARK_ANDROGEN_RESPONSE+++HALLMARK_HYPOXIA+++HALLMARK_IL2_STAT5_SIGNALING+++HALLMARK_P53_PATHWAY
10398	'MYL9'	mRNA	HALLMARK_APICAL_JUNCTION+++HALLMARK_EPITHELIAL_MESENCHYMAL_TRANSITION	H	HALLMARK_APICAL_JUNCTION+++HALLMARK_EPITHELIAL_MESENCHYMAL_TRANSITION
104	'ADARB1'	mRNA			
10435	'CDC42EP2'	mRNA	HALLMARK_MITOTIC_SPINDLE	H	HALLMARK_MITOTIC_SPINDLE
10440	'TIMM17A'	mRNA	HALLMARK_OXIDATIVE_PHOSPHORYLATION	H	HALLMARK_OXIDATIVE_PHOSPHORYLATION
10447	'FAM3C'	mRNA			
10466	'COG5'	mRNA			
10468	'FST'	mRNA	HALLMARK_MYOGENESIS	H	HALLMARK_MYOGENESIS
10477	'UBE2E3'	mRNA			
10483	'SEC23B'	mRNA			
1051	'CEBPB'	mRNA	HALLMARK_COMPLEMENT+++HALLMARK_TNFA_SIGNALING_VIA_NFKB+++HALLMARK_UNFOLDED_PROTEIN_RESPONSE	H	HALLMARK_COMPLEMENT+++HALLMARK_TNFA_SIGNALING_VIA_NFKB+++HALLMARK_UNFOLDED_PROTEIN_RESPONSE
10512	'SEMA3C'	mRNA	HALLMARK_PEROXISOME	H	HALLMARK_PEROXISOME
10525	'HYOU1'	mRNA	HALLMARK_UNFOLDED_PROTEIN_RESPONSE	H	HALLMARK_UNFOLDED_PROTEIN_RESPONSE
10528	'NOP56'	mRNA	HALLMARK_E2F_TARGETS+++HALLMARK_MYC_TARGETS_V1+++HALLMARK_MYC_TARGETS_V2+++HALLMARK_UNFOLDED_PROTEIN_RESPONSE	H	HALLMARK_E2F_TARGETS+++HALLMARK_MYC_TARGETS_V1+++HALLMARK_MYC_TARGETS_V2+++HALLMARK_UNFOLDED_PROTEIN_RESPONSE
10535	'RNASEH2A'	mRNA	HALLMARK_E2F_TARGETS+++HALLMARK_ESTROGEN_RESPONSE_LATE	H	HALLMARK_E2F_TARGETS+++HALLMARK_ESTROGEN_RESPONSE_LATE
1054	'CEBPG'	mRNA	HALLMARK_UNFOLDED_PROTEIN_RESPONSE+++HALLMARK_UV_RESPONSE_UP	H	HALLMARK_UNFOLDED_PROTEIN_RESPONSE+++HALLMARK_UV_RESPONSE_UP
10560	'SLC19A2'	mRNA	HALLMARK_ESTROGEN_RESPONSE_EARLY+++HALLMARK_P53_PATHWAY	H	HALLMARK_ESTROGEN_RESPONSE_EARLY+++HALLMARK_P53_PATHWAY
10579	'TACC2'	mRNA			
1058	'CENPA'	mRNA	HALLMARK_G2M_CHECKPOINT+++HALLMARK_GLYCOLYSIS	H	HALLMARK_G2M_CHECKPOINT+++HALLMARK_GLYCOLYSIS
10580	'SORBS1'	mRNA	HALLMARK_ADIPOGENESIS+++HALLMARK_MYOGENESIS	H	HALLMARK_ADIPOGENESIS+++HALLMARK_MYOGENESIS
1059	'CENPB'	mRNA			
10592	'SMC2'	mRNA	HALLMARK_G2M_CHECKPOINT	H	HALLMARK_G2M_CHECKPOINT
10600	'USP16'	mRNA	HALLMARK_COMPLEMENT	H	HALLMARK_COMPLEMENT
10602	'CDC42EP3'	mRNA			
10606	'PAICS'	mRNA	HALLMARK_E2F_TARGETS	H	HALLMARK_E2F_TARGETS
10611	'PDLIM5'	mRNA	HALLMARK_ANDROGEN_RESPONSE+++HALLMARK_MITOTIC_SPINDLE+++HALLMARK_TNFA_SIGNALING_VIA_NFKB+++HALLMARK_UV_RESPONSE_DN+++HALLMARK_XENOBIOTIC_METABOLISM	H	HALLMARK_ANDROGEN_RESPONSE+++HALLMARK_MITOTIC_SPINDLE+++HALLMARK_TNFA_SIGNALING_VIA_NFKB+++HALLMARK_UV_RESPONSE_DN+++HALLMARK_XENOBIOTIC_METABOLISM
10616	'RBCK1'	mRNA	HALLMARK_GLYCOLYSIS+++HALLMARK_INTERFERON_GAMMA_RESPONSE	H	HALLMARK_GLYCOLYSIS+++HALLMARK_INTERFERON_GAMMA_RESPONSE
10620	'ARID3B'	mRNA			
10636	'RGS14'	mRNA			
10714	'POLD3'	mRNA	HALLMARK_DNA_REPAIR+++HALLMARK_E2F_TARGETS	H	HALLMARK_DNA_REPAIR+++HALLMARK_E2F_TARGETS
10723	'SLC12A7'	mRNA			
1075	'CTSC'	mRNA	HALLMARK_COMPLEMENT+++HALLMARK_MTORC1_SIGNALING+++HALLMARK_PROTEIN_SECRETION	H	HALLMARK_COMPLEMENT+++HALLMARK_MTORC1_SIGNALING+++HALLMARK_PROTEIN_SECRETION
10758	'TRAF3IP2'	mRNA			
10769	'PLK2'	mRNA	HALLMARK_P53_PATHWAY+++HALLMARK_TNFA_SIGNALING_VIA_NFKB	H	HALLMARK_P53_PATHWAY+++HALLMARK_TNFA_SIGNALING_VIA_NFKB
10797	'MTHFD2'	mRNA	HALLMARK_E2F_TARGETS+++HALLMARK_INTERFERON_GAMMA_RESPONSE+++HALLMARK_MTORC1_SIGNALING+++HALLMARK_UNFOLDED_PROTEIN_RESPONSE	H	HALLMARK_E2F_TARGETS+++HALLMARK_INTERFERON_GAMMA_RESPONSE+++HALLMARK_MTORC1_SIGNALING+++HALLMARK_UNFOLDED_PROTEIN_RESPONSE
10802	'SEC24A'	mRNA			
10808	'HSPH1'	mRNA	HALLMARK_FATTY_ACID_METABOLISM	H	HALLMARK_FATTY_ACID_METABOLISM
10825	'NEU3'	mRNA			
10846	'PDE10A'	mRNA			
10849	'POLR1G'	mRNA			
10885	'WDR3'	mRNA			
10919	'EHMT2'	mRNA			
10927	'SPIN1'	mRNA			
10938	'EHD1'	mRNA	HALLMARK_COMPLEMENT+++HALLMARK_TNFA_SIGNALING_VIA_NFKB	H	HALLMARK_COMPLEMENT+++HALLMARK_TNFA_SIGNALING_VIA_NFKB
10946	'SF3A3'	mRNA	HALLMARK_DNA_REPAIR	H	HALLMARK_DNA_REPAIR
10949	'HNRNPA0'	mRNA			
10951	'CBX1'	mRNA	HALLMARK_G2M_CHECKPOINT	H	HALLMARK_G2M_CHECKPOINT
10954	'PDIA5'	mRNA	HALLMARK_UNFOLDED_PROTEIN_RESPONSE	H	HALLMARK_UNFOLDED_PROTEIN_RESPONSE
10956	'OS9'	mRNA			
10957	'PNRC1'	mRNA	HALLMARK_CHOLESTEROL_HOMEOSTASIS+++HALLMARK_HYPOXIA+++HALLMARK_TNFA_SIGNALING_VIA_NFKB	H	HALLMARK_CHOLESTEROL_HOMEOSTASIS+++HALLMARK_HYPOXIA+++HALLMARK_TNFA_SIGNALING_VIA_NFKB
11007	'CCDC85B'	mRNA			
11010	'GLIPR1'	mRNA	HALLMARK_EPITHELIAL_MESENCHYMAL_TRANSITION	H	HALLMARK_EPITHELIAL_MESENCHYMAL_TRANSITION
11015	'KDELR3'	mRNA	HALLMARK_GLYCOLYSIS+++HALLMARK_HYPOXIA+++HALLMARK_UNFOLDED_PROTEIN_RESPONSE	H	HALLMARK_GLYCOLYSIS+++HALLMARK_HYPOXIA+++HALLMARK_UNFOLDED_PROTEIN_RESPONSE
11030	'RBPMS'	mRNA	HALLMARK_UV_RESPONSE_DN	H	HALLMARK_UV_RESPONSE_DN
1104	'RCC1'	mRNA			
11040	'PIM2'	mRNA			
11041	'B4GAT1'	mRNA			
11057	'ABHD2'	mRNA	HALLMARK_ANDROGEN_RESPONSE+++HALLMARK_ESTROGEN_RESPONSE_EARLY+++HALLMARK_ESTROGEN_RESPONSE_LATE	H	HALLMARK_ANDROGEN_RESPONSE+++HALLMARK_ESTROGEN_RESPONSE_EARLY+++HALLMARK_ESTROGEN_RESPONSE_LATE
11080	'DNAJB4'	mRNA	HALLMARK_TNFA_SIGNALING_VIA_NFKB	H	HALLMARK_TNFA_SIGNALING_VIA_NFKB
11082	'ESM1'	mRNA			
11091	'WDR5'	mRNA			
11098	'PRSS23'	mRNA	HALLMARK_COAGULATION+++HALLMARK_ESTROGEN_RESPONSE_EARLY+++HALLMARK_ESTROGEN_RESPONSE_LATE	H	HALLMARK_COAGULATION+++HALLMARK_ESTROGEN_RESPONSE_EARLY+++HALLMARK_ESTROGEN_RESPONSE_LATE
11120	'BTN2A1'	mRNA			
11130	'ZWINT'	mRNA	HALLMARK_DNA_REPAIR	H	HALLMARK_DNA_REPAIR
11138	'TBC1D8'	mRNA			
11153	'FICD'	mRNA			
11165	'NUDT3'	mRNA			
11167	'FSTL1'	mRNA	HALLMARK_ANGIOGENESIS+++HALLMARK_EPITHELIAL_MESENCHYMAL_TRANSITION	H	HALLMARK_ANGIOGENESIS+++HALLMARK_EPITHELIAL_MESENCHYMAL_TRANSITION
11168	'PSIP1'	mRNA	HALLMARK_E2F_TARGETS	H	HALLMARK_E2F_TARGETS
11169	'WDHD1'	mRNA			
11174	'ADAMTS6'	mRNA			
11182	'SLC2A6'	mRNA	HALLMARK_TNFA_SIGNALING_VIA_NFKB	H	HALLMARK_TNFA_SIGNALING_VIA_NFKB
11198	'SUPT16H'	mRNA			
11214	'AKAP13'	mRNA	HALLMARK_MITOTIC_SPINDLE	H	HALLMARK_MITOTIC_SPINDLE
11221	'DUSP10'	mRNA			
11226	'GALNT6'	mRNA			
11228	'RASSF8'	mRNA			
11231	'SEC63'	mRNA			
11245	'GPR176'	mRNA			
11252	'PACSIN2'	mRNA			
11260	'XPOT'	mRNA	HALLMARK_MYC_TARGETS_V1+++HALLMARK_UNFOLDED_PROTEIN_RESPONSE	H	HALLMARK_MYC_TARGETS_V1+++HALLMARK_UNFOLDED_PROTEIN_RESPONSE
112611	'RWDD2A'	mRNA			
11270	'NRM'	mRNA			
11273	'ATXN2L'	mRNA			
112936	'VPS26B'	mRNA			
112939	'NACC1'	mRNA			
112950	'MED8'	mRNA			
113146	'AHNAK2'	mRNA			
113174	'SAAL1'	mRNA			
113277	'TMEM106A'	mRNA			
11335	'CBX3'	mRNA	HALLMARK_MYC_TARGETS_V1+++HALLMARK_MYC_TARGETS_V2	H	HALLMARK_MYC_TARGETS_V1+++HALLMARK_MYC_TARGETS_V2
11338	'U2AF2'	mRNA			
11342	'RNF13'	mRNA			
11343	'MGLL'	mRNA	HALLMARK_ADIPOGENESIS+++HALLMARK_FATTY_ACID_METABOLISM+++HALLMARK_UV_RESPONSE_DN	H	HALLMARK_ADIPOGENESIS+++HALLMARK_FATTY_ACID_METABOLISM+++HALLMARK_UV_RESPONSE_DN
11346	'SYNPO'	mRNA	HALLMARK_KRAS_SIGNALING_DN+++HALLMARK_MITOTIC_SPINDLE	H	HALLMARK_KRAS_SIGNALING_DN+++HALLMARK_MITOTIC_SPINDLE
113829	'SLC35A4'	mRNA			
113878	'DTX2'	mRNA	HALLMARK_NOTCH_SIGNALING	H	HALLMARK_NOTCH_SIGNALING
114548	'NLRP3'	mRNA	HALLMARK_ALLOGRAFT_REJECTION+++HALLMARK_INFLAMMATORY_RESPONSE	H	HALLMARK_ALLOGRAFT_REJECTION+++HALLMARK_INFLAMMATORY_RESPONSE
114787	'GPRIN1'	mRNA			
114789	'SLC25A25'	mRNA			
114794	'ELFN2'	mRNA			
114841035	'LOC114841035'	mRNA			
114880	'OSBPL6'	mRNA			
114897	'C1QTNF1'	mRNA			
114904	'C1QTNF6'	mRNA			
114907	'FBXO32'	mRNA			
115106	'HAUS1'	mRNA			
115752	'DIS3L'	mRNA			
116028	'RMI2'	mRNA			
116068	'LYSMD3'	mRNA			
116150	'NUS1'	mRNA			
116151	'FAM210B'	mRNA			
116372	'LYPD1'	mRNA			
116442	'RAB39B'	mRNA			
116496	'NIBAN1'	mRNA	HALLMARK_CHOLESTEROL_HOMEOSTASIS+++HALLMARK_MTORC1_SIGNALING	H	HALLMARK_CHOLESTEROL_HOMEOSTASIS+++HALLMARK_MTORC1_SIGNALING
1182	'CLCN3'	mRNA	HALLMARK_HEME_METABOLISM+++HALLMARK_PROTEIN_SECRETION	H	HALLMARK_HEME_METABOLISM+++HALLMARK_PROTEIN_SECRETION
118738	'ZNF488'	mRNA			
12	'SERPINA3'	mRNA	HALLMARK_ESTROGEN_RESPONSE_LATE+++HALLMARK_KRAS_SIGNALING_UP	H	HALLMARK_ESTROGEN_RESPONSE_LATE+++HALLMARK_KRAS_SIGNALING_UP
121551	'ABTB3'	mRNA			
122553	'TRAPPC6B'	mRNA			
122953	'JDP2'	mRNA			
123096	'SLC25A29'	mRNA			
123606	'NIPA1'	mRNA			
124045	'SPATA33'	mRNA			
124222	'PAQR4'	mRNA			
124904930	'LOC124904930'	mRNA			
124976	'SPNS2'	mRNA			
126014	'OSCAR'	mRNA			
126133	'ALDH16A1'	mRNA			
1263	'PLK3'	mRNA	HALLMARK_P53_PATHWAY	H	HALLMARK_P53_PATHWAY
126321	'MFSD12'	mRNA			
1267	'CNP'	mRNA	HALLMARK_INTERFERON_ALPHA_RESPONSE+++HALLMARK_UV_RESPONSE_UP	H	HALLMARK_INTERFERON_ALPHA_RESPONSE+++HALLMARK_UV_RESPONSE_UP
127703	'C1orf216'	mRNA			
127829	'ARL8A'	mRNA	HALLMARK_MITOTIC_SPINDLE	H	HALLMARK_MITOTIC_SPINDLE
128125822	'LOC128125822'	mRNA			
128866	'CHMP4B'	mRNA			
128977	'C22orf39'	mRNA			
129607	'CMPK2'	mRNA	HALLMARK_DNA_REPAIR+++HALLMARK_INTERFERON_ALPHA_RESPONSE+++HALLMARK_INTERFERON_GAMMA_RESPONSE	H	HALLMARK_DNA_REPAIR+++HALLMARK_INTERFERON_ALPHA_RESPONSE+++HALLMARK_INTERFERON_GAMMA_RESPONSE
130367	'SGPP2'	mRNA			
130574	'LYPD6'	mRNA			
1314	'COPA'	mRNA	HALLMARK_EPITHELIAL_MESENCHYMAL_TRANSITION	H	HALLMARK_EPITHELIAL_MESENCHYMAL_TRANSITION
1315	'COPB1'	mRNA	HALLMARK_PROTEIN_SECRETION	H	HALLMARK_PROTEIN_SECRETION
131566	'DCBLD2'	mRNA	HALLMARK_APICAL_SURFACE+++HALLMARK_INFLAMMATORY_RESPONSE+++HALLMARK_KRAS_SIGNALING_UP	H	HALLMARK_APICAL_SURFACE+++HALLMARK_INFLAMMATORY_RESPONSE+++HALLMARK_KRAS_SIGNALING_UP
1317	'SLC31A1'	mRNA	HALLMARK_INFLAMMATORY_RESPONSE	H	HALLMARK_INFLAMMATORY_RESPONSE
133	'ADM'	mRNA	HALLMARK_HYPOXIA+++HALLMARK_INFLAMMATORY_RESPONSE	H	HALLMARK_HYPOXIA+++HALLMARK_INFLAMMATORY_RESPONSE
133619	'PRRC1'	mRNA			
133746	'JMY'	mRNA			
134147	'CMBL'	mRNA	HALLMARK_ADIPOGENESIS	H	HALLMARK_ADIPOGENESIS
134429	'STARD4'	mRNA	HALLMARK_CHOLESTEROL_HOMEOSTASIS+++HALLMARK_MTORC1_SIGNALING	H	HALLMARK_CHOLESTEROL_HOMEOSTASIS+++HALLMARK_MTORC1_SIGNALING
134553	'C5orf24'	mRNA			
135	'ADORA2A'	mRNA			
1355	'COX15'	mRNA	HALLMARK_OXIDATIVE_PHOSPHORYLATION	H	HALLMARK_OXIDATIVE_PHOSPHORYLATION
137492	'VPS37A'	mRNA			
1386	'ATF2'	mRNA			
138716	'RPP25L'	mRNA			
1390	'CREM'	mRNA			
140609	'NEK7'	mRNA	HALLMARK_HEME_METABOLISM+++HALLMARK_UV_RESPONSE_DN	H	HALLMARK_HEME_METABOLISM+++HALLMARK_UV_RESPONSE_DN
1407	'CRY1'	mRNA			
140707	'BRI3BP'	mRNA			
140735	'DYNLL2'	mRNA	HALLMARK_MITOTIC_SPINDLE	H	HALLMARK_MITOTIC_SPINDLE
140876	'RIPOR3'	mRNA			
143	'PARP4'	mRNA			
1434	'CSE1L'	mRNA	HALLMARK_E2F_TARGETS	H	HALLMARK_E2F_TARGETS
1435	'CSF1'	mRNA	HALLMARK_ALLOGRAFT_REJECTION+++HALLMARK_IL2_STAT5_SIGNALING+++HALLMARK_IL6_JAK_STAT3_SIGNALING+++HALLMARK_INFLAMMATORY_RESPONSE+++HALLMARK_INTERFERON_ALPHA_RESPONSE+++HALLMARK_TNFA_SIGNALING_VIA_NFKB	H	HALLMARK_ALLOGRAFT_REJECTION+++HALLMARK_IL2_STAT5_SIGNALING+++HALLMARK_IL6_JAK_STAT3_SIGNALING+++HALLMARK_INFLAMMATORY_RESPONSE+++HALLMARK_INTERFERON_ALPHA_RESPONSE+++HALLMARK_TNFA_SIGNALING_VIA_NFKB
143903	'LAYN'	mRNA	HALLMARK_APICAL_JUNCTION	H	HALLMARK_APICAL_JUNCTION
144100	'PLEKHA7'	mRNA			
144406	'CFAP251'	mRNA			
1454	'CSNK1E'	mRNA	HALLMARK_WNT_BETA_CATENIN_SIGNALING	H	HALLMARK_WNT_BETA_CATENIN_SIGNALING
146059	'CDAN1'	mRNA			
146223	'CMTM4'	mRNA			
1465	'CSRP1'	mRNA	HALLMARK_COAGULATION+++HALLMARK_COMPLEMENT	H	HALLMARK_COAGULATION+++HALLMARK_COMPLEMENT
1468	'SLC25A10'	mRNA	HALLMARK_ADIPOGENESIS+++HALLMARK_GLYCOLYSIS	H	HALLMARK_ADIPOGENESIS+++HALLMARK_GLYCOLYSIS
1488	'CTBP2'	mRNA			
148867	'SLC30A7'	mRNA			
1490	'CCN2'	mRNA	HALLMARK_EPITHELIAL_MESENCHYMAL_TRANSITION+++HALLMARK_HYPOXIA	H	HALLMARK_EPITHELIAL_MESENCHYMAL_TRANSITION+++HALLMARK_HYPOXIA
149041	'RC3H1'	mRNA			
1491	'CTH'	mRNA	HALLMARK_APOPTOSIS+++HALLMARK_GLYCOLYSIS+++HALLMARK_MTORC1_SIGNALING	H	HALLMARK_APOPTOSIS+++HALLMARK_GLYCOLYSIS+++HALLMARK_MTORC1_SIGNALING
149111	'CNIH3'	mRNA			
149175	'MANEAL'	mRNA			
151011	'SEPTIN10'	mRNA			
151742	'PPM1L'	mRNA			
151963	'MB21D2'	mRNA			
152002	'XXYLT1'	mRNA			
152518	'NFXL1'	mRNA			
152926	'PPM1K'	mRNA			
153222	'CREBRF'	mRNA			
153241	'CEP120'	mRNA			
153830	'RNF145'	mRNA			
1540	'CYLD'	mRNA	HALLMARK_APOPTOSIS	H	HALLMARK_APOPTOSIS
154214	'RNF217'	mRNA			
155185	'AMZ1'	mRNA			
157285	'PRAG1'	mRNA			
157574	'FBXO16'	mRNA			
157638	'LRATD2'	mRNA			
157769	'FAM91A1'	mRNA			
157922	'CAMSAP1'	mRNA			
158747	'MOSPD2'	mRNA			
16	'AARS1'	mRNA	HALLMARK_ALLOGRAFT_REJECTION	H	HALLMARK_ALLOGRAFT_REJECTION
1601	'DAB2'	mRNA	HALLMARK_EPITHELIAL_MESENCHYMAL_TRANSITION+++HALLMARK_UV_RESPONSE_DN	H	HALLMARK_EPITHELIAL_MESENCHYMAL_TRANSITION+++HALLMARK_UV_RESPONSE_DN
1604	'CD55'	mRNA	HALLMARK_COMPLEMENT+++HALLMARK_INFLAMMATORY_RESPONSE	H	HALLMARK_COMPLEMENT+++HALLMARK_INFLAMMATORY_RESPONSE
161176	'SYNE3'	mRNA			
161424	'NOP9'	mRNA			
162394	'SLFN5'	mRNA			
162514	'TRPV3'	mRNA			
1627	'DBN1'	mRNA			
162989	'DEDD2'	mRNA			
163882	'CNST'	mRNA			
1643	'DDB2'	mRNA	HALLMARK_DNA_REPAIR+++HALLMARK_P53_PATHWAY	H	HALLMARK_DNA_REPAIR+++HALLMARK_P53_PATHWAY
164312	'LRRN4'	mRNA			
164781	'DAW1'	mRNA			
1649	'DDIT3'	mRNA	HALLMARK_APOPTOSIS+++HALLMARK_HYPOXIA+++HALLMARK_MTORC1_SIGNALING+++HALLMARK_P53_PATHWAY+++HALLMARK_PI3K_AKT_MTOR_SIGNALING	H	HALLMARK_APOPTOSIS+++HALLMARK_HYPOXIA+++HALLMARK_MTORC1_SIGNALING+++HALLMARK_P53_PATHWAY+++HALLMARK_PI3K_AKT_MTOR_SIGNALING
165055	'CCDC138'	mRNA			
1656	'DDX6'	mRNA			
166647	'ADGRA3'	mRNA			
166929	'SGMS2'	mRNA	HALLMARK_INFLAMMATORY_RESPONSE	H	HALLMARK_INFLAMMATORY_RESPONSE
168451	'THAP5'	mRNA			
168620	'BHLHA15'	mRNA			
170463	'SSBP4'	mRNA			
170690	'ADAMTS16'	mRNA			
170954	'PPP1R18'	mRNA			
1719	'DHFR'	mRNA	HALLMARK_MTORC1_SIGNALING	H	HALLMARK_MTORC1_SIGNALING
1786	'DNMT1'	mRNA	HALLMARK_E2F_TARGETS	H	HALLMARK_E2F_TARGETS
1809	'DPYSL3'	mRNA	HALLMARK_EPITHELIAL_MESENCHYMAL_TRANSITION	H	HALLMARK_EPITHELIAL_MESENCHYMAL_TRANSITION
182	'JAG1'	mRNA	HALLMARK_ANGIOGENESIS+++HALLMARK_CHOLESTEROL_HOMEOSTASIS+++HALLMARK_NOTCH_SIGNALING+++HALLMARK_TNFA_SIGNALING_VIA_NFKB+++HALLMARK_WNT_BETA_CATENIN_SIGNALING	H	HALLMARK_ANGIOGENESIS+++HALLMARK_CHOLESTEROL_HOMEOSTASIS+++HALLMARK_NOTCH_SIGNALING+++HALLMARK_TNFA_SIGNALING_VIA_NFKB+++HALLMARK_WNT_BETA_CATENIN_SIGNALING
1827	'RCAN1'	mRNA	HALLMARK_TNFA_SIGNALING_VIA_NFKB	H	HALLMARK_TNFA_SIGNALING_VIA_NFKB
1846	'DUSP4'	mRNA	HALLMARK_TNFA_SIGNALING_VIA_NFKB	H	HALLMARK_TNFA_SIGNALING_VIA_NFKB
1869	'E2F1'	mRNA	HALLMARK_G2M_CHECKPOINT+++HALLMARK_PI3K_AKT_MTOR_SIGNALING	H	HALLMARK_G2M_CHECKPOINT+++HALLMARK_PI3K_AKT_MTOR_SIGNALING
1870	'E2F2'	mRNA	HALLMARK_G2M_CHECKPOINT+++HALLMARK_HEME_METABOLISM	H	HALLMARK_G2M_CHECKPOINT+++HALLMARK_HEME_METABOLISM
1889	'ECE1'	mRNA			
1901	'S1PR1'	mRNA			
1902	'LPAR1'	mRNA	HALLMARK_INFLAMMATORY_RESPONSE+++HALLMARK_UV_RESPONSE_DN	H	HALLMARK_INFLAMMATORY_RESPONSE+++HALLMARK_UV_RESPONSE_DN
1909	'EDNRA'	mRNA			
1942	'EFNA1'	mRNA	HALLMARK_HYPOXIA+++HALLMARK_TNFA_SIGNALING_VIA_NFKB	H	HALLMARK_HYPOXIA+++HALLMARK_TNFA_SIGNALING_VIA_NFKB
1948	'EFNB2'	mRNA			
1958	'EGR1'	mRNA	HALLMARK_TNFA_SIGNALING_VIA_NFKB	H	HALLMARK_TNFA_SIGNALING_VIA_NFKB
195828	'ZNF367'	mRNA			
196	'AHR'	mRNA	HALLMARK_IL2_STAT5_SIGNALING+++HALLMARK_INFLAMMATORY_RESPONSE	H	HALLMARK_IL2_STAT5_SIGNALING+++HALLMARK_INFLAMMATORY_RESPONSE
1960	'EGR3'	mRNA	HALLMARK_APOPTOSIS+++HALLMARK_ESTROGEN_RESPONSE_EARLY+++HALLMARK_ESTROGEN_RESPONSE_LATE+++HALLMARK_TNFA_SIGNALING_VIA_NFKB	H	HALLMARK_APOPTOSIS+++HALLMARK_ESTROGEN_RESPONSE_EARLY+++HALLMARK_ESTROGEN_RESPONSE_LATE+++HALLMARK_TNFA_SIGNALING_VIA_NFKB
196483	'EEF2KMT'	mRNA			
1969	'EPHA2'	mRNA	HALLMARK_P53_PATHWAY+++HALLMARK_XENOBIOTIC_METABOLISM	H	HALLMARK_P53_PATHWAY+++HALLMARK_XENOBIOTIC_METABOLISM
197259	'MLKL'	mRNA			
197320	'ZNF778'	mRNA			
1992	'SERPINB1'	mRNA			
200734	'SPRED2'	mRNA	HALLMARK_IL2_STAT5_SIGNALING	H	HALLMARK_IL2_STAT5_SIGNALING
201158	'TVP23C'	mRNA			
201305	'SPNS3'	mRNA			
201562	'HACD2'	mRNA			
2030	'SLC29A1'	mRNA	HALLMARK_BILE_ACID_METABOLISM+++HALLMARK_ESTROGEN_RESPONSE_LATE	H	HALLMARK_BILE_ACID_METABOLISM+++HALLMARK_ESTROGEN_RESPONSE_LATE
203068	'TUBB'	mRNA	HALLMARK_E2F_TARGETS	H	HALLMARK_E2F_TARGETS
2034	'EPAS1'	mRNA			
2058	'EPRS1'	mRNA	HALLMARK_MTORC1_SIGNALING+++HALLMARK_MYC_TARGETS_V1	H	HALLMARK_MTORC1_SIGNALING+++HALLMARK_MYC_TARGETS_V1
2069	'EREG'	mRNA	HALLMARK_ALLOGRAFT_REJECTION+++HALLMARK_APOPTOSIS+++HALLMARK_INFLAMMATORY_RESPONSE+++HALLMARK_KRAS_SIGNALING_UP	H	HALLMARK_ALLOGRAFT_REJECTION+++HALLMARK_APOPTOSIS+++HALLMARK_INFLAMMATORY_RESPONSE+++HALLMARK_KRAS_SIGNALING_UP
2081	'ERN1'	mRNA	HALLMARK_UNFOLDED_PROTEIN_RESPONSE	H	HALLMARK_UNFOLDED_PROTEIN_RESPONSE
2119	'ETV5'	mRNA	HALLMARK_KRAS_SIGNALING_UP	H	HALLMARK_KRAS_SIGNALING_UP
2131	'EXT1'	mRNA	HALLMARK_GLYCOLYSIS+++HALLMARK_HYPOXIA	H	HALLMARK_GLYCOLYSIS+++HALLMARK_HYPOXIA
2139	'EYA2'	mRNA			
214	'ALCAM'	mRNA	HALLMARK_CHOLESTEROL_HOMEOSTASIS+++HALLMARK_IL2_STAT5_SIGNALING	H	HALLMARK_CHOLESTEROL_HOMEOSTASIS+++HALLMARK_IL2_STAT5_SIGNALING
2149	'F2R'	mRNA	HALLMARK_ALLOGRAFT_REJECTION+++HALLMARK_APOPTOSIS+++HALLMARK_P53_PATHWAY	H	HALLMARK_ALLOGRAFT_REJECTION+++HALLMARK_APOPTOSIS+++HALLMARK_P53_PATHWAY
2150	'F2RL1'	mRNA	HALLMARK_KRAS_SIGNALING_UP+++HALLMARK_TNFA_SIGNALING_VIA_NFKB	H	HALLMARK_KRAS_SIGNALING_UP+++HALLMARK_TNFA_SIGNALING_VIA_NFKB
2152	'F3'	mRNA	HALLMARK_COAGULATION+++HALLMARK_COMPLEMENT+++HALLMARK_HYPOXIA+++HALLMARK_INFLAMMATORY_RESPONSE+++HALLMARK_TNFA_SIGNALING_VIA_NFKB+++HALLMARK_UV_RESPONSE_DN	H	HALLMARK_COAGULATION+++HALLMARK_COMPLEMENT+++HALLMARK_HYPOXIA+++HALLMARK_INFLAMMATORY_RESPONSE+++HALLMARK_TNFA_SIGNALING_VIA_NFKB+++HALLMARK_UV_RESPONSE_DN
2167	'FABP4'	mRNA	HALLMARK_ADIPOGENESIS	H	HALLMARK_ADIPOGENESIS
2171	'FABP5'	mRNA	HALLMARK_CHOLESTEROL_HOMEOSTASIS+++HALLMARK_ESTROGEN_RESPONSE_LATE	H	HALLMARK_CHOLESTEROL_HOMEOSTASIS+++HALLMARK_ESTROGEN_RESPONSE_LATE
2177	'FANCD2'	mRNA			
2181	'ACSL3'	mRNA	HALLMARK_ANDROGEN_RESPONSE+++HALLMARK_MTORC1_SIGNALING	H	HALLMARK_ANDROGEN_RESPONSE+++HALLMARK_MTORC1_SIGNALING
2182	'ACSL4'	mRNA	HALLMARK_FATTY_ACID_METABOLISM+++HALLMARK_PEROXISOME	H	HALLMARK_FATTY_ACID_METABOLISM+++HALLMARK_PEROXISOME
2188	'FANCF'	mRNA			
219	'ALDH1B1'	mRNA			
219333	'USP12'	mRNA	HALLMARK_KRAS_SIGNALING_UP	H	HALLMARK_KRAS_SIGNALING_UP
219654	'ZCCHC24'	mRNA			
219699	'UNC5B'	mRNA			
219855	'SLC37A2'	mRNA			
220	'ALDH1A3'	mRNA	HALLMARK_ANDROGEN_RESPONSE+++HALLMARK_KRAS_SIGNALING_UP	H	HALLMARK_ANDROGEN_RESPONSE+++HALLMARK_KRAS_SIGNALING_UP
220002	'CYB561A3'	mRNA			
220064	'LTO1'	mRNA			
220323	'OAF'	mRNA			
221079	'ARL5B'	mRNA			
221092	'HNRNPUL2'	mRNA			
221749	'PXDC1'	mRNA			
2222	'FDFT1'	mRNA	HALLMARK_CHOLESTEROL_HOMEOSTASIS+++HALLMARK_ESTROGEN_RESPONSE_EARLY+++HALLMARK_ESTROGEN_RESPONSE_LATE	H	HALLMARK_CHOLESTEROL_HOMEOSTASIS+++HALLMARK_ESTROGEN_RESPONSE_EARLY+++HALLMARK_ESTROGEN_RESPONSE_LATE
222658	'KCTD20'	mRNA			
2237	'FEN1'	mRNA	HALLMARK_DNA_REPAIR+++HALLMARK_UV_RESPONSE_UP	H	HALLMARK_DNA_REPAIR+++HALLMARK_UV_RESPONSE_UP
224	'ALDH3A2'	mRNA	HALLMARK_ESTROGEN_RESPONSE_LATE+++HALLMARK_FATTY_ACID_METABOLISM	H	HALLMARK_ESTROGEN_RESPONSE_LATE+++HALLMARK_FATTY_ACID_METABOLISM
2241	'FER'	mRNA			
2247	'FGF2'	mRNA	HALLMARK_EPITHELIAL_MESENCHYMAL_TRANSITION+++HALLMARK_MYOGENESIS	H	HALLMARK_EPITHELIAL_MESENCHYMAL_TRANSITION+++HALLMARK_MYOGENESIS
2274	'FHL2'	mRNA	HALLMARK_ESTROGEN_RESPONSE_EARLY+++HALLMARK_UV_RESPONSE_DN	H	HALLMARK_ESTROGEN_RESPONSE_EARLY+++HALLMARK_UV_RESPONSE_DN
22820	'COPG1'	mRNA			
22822	'PHLDA1'	mRNA	HALLMARK_IL2_STAT5_SIGNALING+++HALLMARK_TNFA_SIGNALING_VIA_NFKB	H	HALLMARK_IL2_STAT5_SIGNALING+++HALLMARK_TNFA_SIGNALING_VIA_NFKB
22848	'AAK1'	mRNA			
